# Evaluating the “Zindagi Mehfooz” Electronic Immunization Registry and Suite of Digital Health Interventions to Improve the Coverage and Timeliness of Immunization Services in Sindh, Pakistan: Mixed Methods Study

**DOI:** 10.2196/52792

**Published:** 2024-10-11

**Authors:** Patricia Mechael, Sara Gilani, Ahsan Ahmad, Amnesty LeFevre, Diwakar Mohan, Asra Memon, Mubarak Taighoon Shah, Danya Arif Siddiqi, Subhash Chandir, Riswana Soundardjee

**Affiliations:** 1 HealthEnabled Washington, DC United States; 2 Johns Hopkins Bloomberg School of Public Health Baltimore, MD United States; 3 Gallup Pakistan Karachi Pakistan; 4 Independent Consultant Islamabad Pakistan; 5 University of Cape Town Cape Town South Africa; 6 IRD Global Karachi Pakistan; 7 IRD Pakistan Karachi Pakistan; 8 Gavi, The Vaccine Alliance Geneva Switzerland

**Keywords:** electronic immunization registry, registry, Zindagi Mehfooz, vaccination, alert, reminder, dashboard, survey, cost, economic, digital health, immunization, children, pediatrics, equity, accessibility, text messages, SMS, zero dose

## Abstract

**Background:**

The Zindagi Mehfooz (safe life; ZM) electronic immunization registry (EIR) is a comprehensive suite of digital health interventions that aims to improve equitable access, timeliness, and coverage of child immunizations through a smartphone-based app for vaccinators, web-based dashboards for supervisors and managers, text message alerts and reminders for caregivers, and a call center. It has been implemented at scale in Sindh Province, Pakistan.

**Objective:**

This study aimed to present findings from an evaluation of the ZM-EIR suite of digital health interventions in order to improve data availability and use as a contribution, among other immunization program interventions, to enhanced immunization outcomes for children aged 12-23 months in Sindh Province.

**Methods:**

The mixed methods study included (1) analysis of ZM-EIR system data to identify high-, moderate-, and low-adoption and compliance sites; (2) in-depth interviews with caregivers, vaccinators, supervisors, and managers in the Expanded Program for Immunization (EPI); and (3) pre-post outcome evaluation using vaccine coverage from the Multiple Indicator Cluster Surveys (MICS) 2014 and 2018-2019. Key outcomes of interest were improved data availability, use and contribution to immunization outcomes, including receipt of individual antigens (Bacillus Calmette-Guérin [BCG], pentavalent [Penta] 1-3, measles), full immunization (all antigens), and zero-dose children defined as children aged 6-23 months who have not received the first dosage of the diphtheria-pertussis-tetanus 1/Penta vaccine.

**Results:**

By registering newborns, providing alerts and reminders, and tracking their immunization completion, the ZM-EIR improved data availability and use in the EPI. The ZM-EIR was well received by EPI administrators, supervisors, vaccinators, and caregivers. The key benefit highlighted by ZM-EIR users was a list of children who missed scheduled vaccines (defaulters). Through greater availability and use of data, the ZM-EIR implementation, as part of a broader package of immunization program–strengthening activities in Sindh Province, may have contributed to an increase in immunization coverage and timeliness for BCG vaccinations and a decrease in zero-dose children in 2018-2019 from 2014. Additional findings from the study included the dual burden of reporting on paper and gender-related considerations of female caregivers not wanting to provide their phone numbers to male vaccinators, creating barriers to greater uptake of the ZM-EIR.

**Conclusions:**

The ZM-EIR is a promising technology platform that has increased the availability and use of immunization data, which may have contributed, along with other intensive immunization program interventions, to improvements in immunization outcomes through systematic registration of children, alerts and reminders, and increased use of data for planning and monitoring by the EPI.

**Trial Registration:**

ISRCTN Registry ISRCTN23078223; https://doi.org/10.1186/ISRCTN23078223

## Introduction

The *Zindagi Mehfooz* (*safe life*; ZM) electronic immunization registry (EIR) is a comprehensive suite of digital health interventions that aims to improve equitable access, timeliness, and coverage of child immunizations through a smartphone-based app for vaccinators, web-based dashboards for supervisors and managers, text message alerts and reminders for caregivers, and a call center. It has been implemented at scale in Sindh Province, Pakistan.

Electronic immunization registries (EIRs) are digitized case-based immunization record–keeping systems designed to improve the collection, analysis, and use of immunization program data to improve the efficiency, equity, and coverage of immunization programs [1]. A 2015 systematic review [2] of aggregate immunization information systems in high-income settings highlighted the main pathways through which such systems improve immunization outcomes, such as coverage, although only 1 study in Australia measured such an association. The benefits of aggregate systems align with more recent reviews and studies of EIRs that record and track data for individual children and generate aggregate data for immunization program monitoring and planning [3]. These benefits include client reminders, client vaccination status, missed vaccinations, and vaccination program management and accountability [2,3]. Improved access to better data can support evidence-based decisions, planning, and operations for the immunization program, which, in turn, will lead to better delivery of immunization services [4]. When EIR data are triangulated with other primary health care services, recorded births, and population denominators, EIRs can enable population-level analysis to identify areas where the number of children vaccinated is lower than the target population [1,5]. In this way, EIRs can improve the identification and reach of zero-dose children (ie, children who have not received a diphtheria-pertussis-tetanus [DPT] 1 vaccine) and provide targeted service improvements and extended outreach services to those areas and communities. EIRs can also address demand-side challenges by sending automated reminders to caregivers, providing access to educational messages and service-related updates and improving the quality of services at vaccination centers [5].

Because EIRs are intended to support immunization programs with improved data-driven decision-making, the evaluation of data use is also important [6]. Drivers of EIR use include organizational factors (supervision, support, resources, facility type, location, and volume of clients), technical factors (infrastructure, electricity, and internet connectivity), and behavioral factors (training, capacity, and motivation of system users) [6]. A recent study [7] using EIR data to evaluate the timeliness of vaccination systematically found mixed results, with the conclusion that the overall reliance of the study on system-level data was insufficient to evaluate its impact on immunization outcomes. A review of the evidence for EIRs reveals a moderate number of process evaluations, few pilot studies, and no large-scale effectiveness studies of EIRs [3].

The ZM-EIR comprises complementary digital health interventions that satisfy and go beyond the conventional definition of an EIR. The ZM-EIR aims to strengthen Expanded Program for Immunization (EPI) service delivery and the broader health system by addressing both supply- and demand-side barriers that contribute to suboptimal use of immunization services. On the supply side, it is designed to enable health workers to track the immunization history for individual children and assess catch-up immunization schedules, support motivation among health workers by reducing the excessive burden of paperwork and demonstrating the value that quality data can contribute to their workflow [4], and reduce the occurrence of poor reporting, management, supervision, and monitoring of vaccinators. On the demand side, the ZM-EIR is designed to overcome challenges associated with low uptake of immunization services due to a lack of awareness and motivation among parents, as well as the inability to remember vaccine appointments.

Pakistan represents 1 of the 6 countries in the world with the highest concentration of zero-dose children. In 2012-2013, only about half (58.3%) of children aged 12-23 months in Pakistan received all basic vaccinations [8], according to the Pakistan Demographic and Health Survey 2012-2013, with marked geographic variation across the country. Sindh Province recorded full immunization coverage of 35% [9] in 2014, according to the Multiple Indicator Cluster Survey (MICS) conducted in 2014. Sindh Province is home to 47.9 million people and comprised 28 districts during the time frame covered by the evaluation, with around 1500 fixed immunization centers that conduct outreach activities, as well as over 200 private clinics [10]. The provincial capital, Karachi, is Pakistan’s largest capital city and economic hub representing a large urban population. Most childhood vaccines in the district are delivered through public sector immunization centers or outreach activities managed by the national EPI [10].

As of August 2022, the ZM-EIR was scaled to cover all of Sindh Province, including 2 new districts that were not covered during the evaluation period. The ZM-EIR has also been introduced in other areas of Pakistan, including the Federal Capital (Islamabad), Khyber Pakhtunkhwa Province, and the Gilgit-Baltistan region, and has been used by over 5000 vaccinators at public and private immunization clinics to enroll over 7 million children.

This paper aimed to summarize findings from a mixed methods evaluation of ZM-EIR implementation in Sindh Province. Retrospective evaluations of program implementation are difficult to design and carry out due to the lack of counterfactuals and recall biases. Habicht et al [11] proposed “adequacy” as the minimum level of evidence in support of a program’s impact part of an evaluation. We used a range of data sources to support the hypothesis that the ZM-EIR intervention may have contributed to the outcomes achieved by the immunization program. We showed that ZM intervention activities were implemented adequately, accepted (provider and caregiver perceptions of the ZM-EIR), and adopted (vaccinator use of the ZM-EIR) and may have contributed to improved coverage (changes in antigen coverage) and timeliness.

## Methods

### Intervention Description

The ZM-EIR includes the following functionalities:

- Web interface for data visualization
- Mobile-based data entry and access for vaccinators
- Geospatial data for vaccination events
- 2-way interactive SMS reminders for parents/caregivers
- Call center to answer queries of parents/caregivers
- Offline mode to work in areas of poor connectivity
- Child registry for enrolling newborns or never-vaccinated children
- Immunization decision support system (iDSS) to guide vaccinators on routine and catch-up immunizations
- Defaulter reports for vaccinators
- Gamified videos for training vaccinators
- Artificial intelligence (AI)-based predictive analytics to identify children highly likely to drop out
- AI-based chatbot to address caregiver queries and concerns in real time
- Global System for Mobile Communications (GSM)-based tracking of vaccinators during work hours
- Quick response (QR) code–based tracking for unique identification of children and women

**Figure 1:**
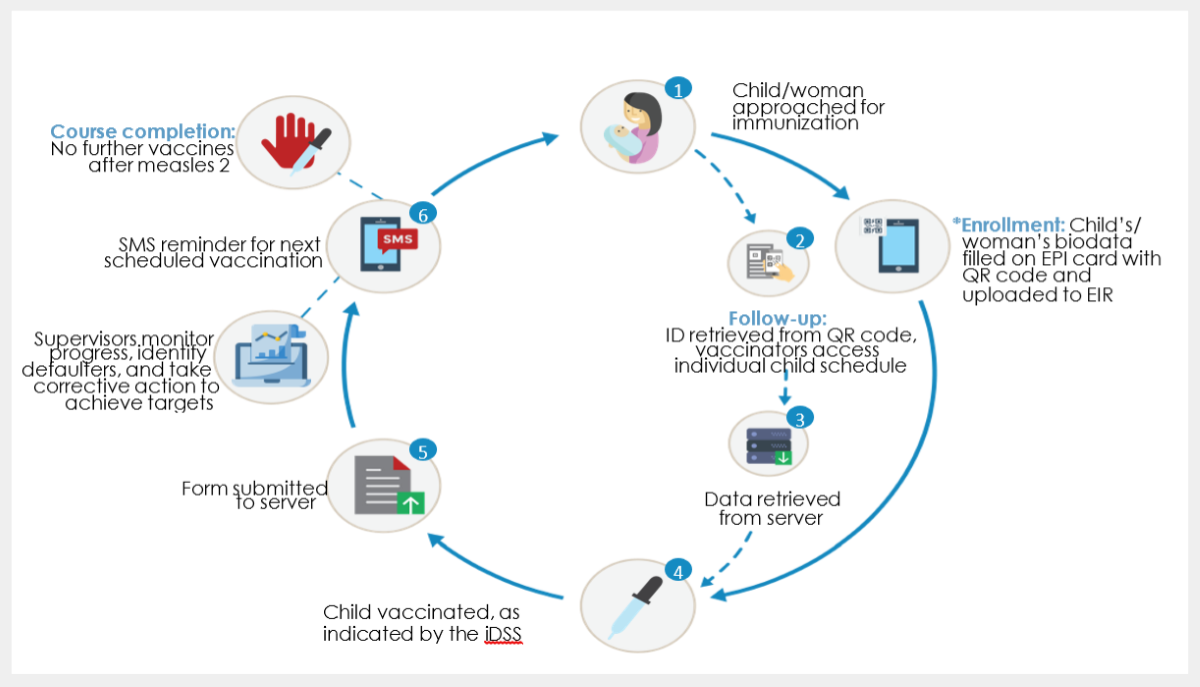
Overview of the ZM system. EIR: electronic immunization registry; EPI: Expanded Program for Immunization; iDSS: immunization decision support system; QR: quick response; ZM: Zindagi Mehfooz (safe life).

These functionalities center around a mobile phone–based registry with a suite of complementary interventions. More details and underlying research that have contributed to the development of the intervention over the past decade have been published elsewhere [12-18]. See Figure 1 for a graphical overview of the ZM-EIR intervention. Although the intervention can capture the immunization of pregnant women, this research focused on its use for routine immunization of children.

The ZM mobile Android app is used to capture data for each point of contact between the immunization service, caregivers, and their children. At the time of first enrollment, the child’s data are entered on the paper EPI card and into the ZM-EIR. A unique QR code is printed and affixed to the home-based paper record and linked to the electronic system. During follow-up visits, the child’s ID is retrieved by scanning the QR code, giving the vaccinator access to the child’s vaccine schedule. For each vaccination received, the data are entered into the ZM app and linked to the server. All data entered into the ZM app are collated on a central web-based dashboard, which supervisors can use to monitor progress, identify defaulters, and take corrective action to achieve targets. The decision support system (DSS) uses a built-in algorithm to automatically calculate which vaccine is to be administered based on the child’s date of birth and immunization history. This minimizes the chance of missed immunization and enables the vaccinators to provide age-appropriate and timely immunization. In addition to recording vaccination activities within the ZM intervention, vaccinators were required to document their work in paper-based registers that were then manually tabulated and submitted as part of monthly reporting activities.

To remind caregivers of when they need to next visit the clinic, up to 3 automatic personalized SMS reminders are sent 1 day before, on the day of, and 6 days after the scheduled immunization date (if the child did not make the scheduled appointment). A child is considered to have completed the full course of immunizations after the second dose of the measles vaccine.

In addition to the inclusion of standard EIR features (as illustrated in Table 1) [3], the ZM-EIR includes AI-based predictive analytics to identify children more likely to drop out; automated SMS reminders; a call center; and an AI chatbot for caregiver questions, concerns, and complaints. The suite of interventions also includes geospatial data for vaccination events, geospatial data to track each child’s immunization progress, real-time tracking of vaccinators with Geographic Information System (GIS) tracking to improve monitoring and accountability, simultaneous supervision of multiple teams, monitoring of attendance and mobility of field staff, and gamified videos and training for vaccinators.

**Table 1:** Comparison of ZM^a^ and ideal EIR^b^ characteristics and functionalities.

Feature	ZM-EIR	Ideal requirements of an EIR [19]
Registration and search	The ZM-EIR is a registry to *enroll all newborns* or never-vaccinated children with a *unique ID/QR*^c^ *code* assigned to each woman and child; a web interface *allows data visualization*, *mobile-based data entry*, and *access for vaccinators*.	Enrollment at birth, client management, unique and unequivocal ID
Patient records	Registration includes all relevant *demographic and location data of the patient* and facility with *geolocation*, *barcoded vaccine vials* linked with vaccine records, and an *offline mode* for areas with low mobile/data coverage (*not linked to other health areas*).	Individual demographic data, vaccine event data, nonroutine (campaign and outreach) vaccine events, and linkages to other health areas
Vaccination monitoring and follow-up	The *iDSS*^d^ guides vaccinators on routine and catch-up immunizations and provides *defaulter reports* for vaccinators and interactive *SMS reminders* for parents/caregivers.	Clinical decision support, identification of undervaccinated children/missed vaccination, and reminder and recall messages
Registry	*Vaccinators are registered* within the system and assigned to a *health facility registered* to a district management unit.	*Health facility registration and management*
Stock	The *vaccine stock is tracked* through the number and type of vaccinations given by vaccinator, facility, and district.	*Stock management*
Data and reporting	*Geospatial analysis* maps pockets of undervaccinated children with exact locations to enable follow-up with households refusing vaccination. There is a call center for complaints and adverse events following immunization *(AEFI) reporting.*	Data aggregation at different geographic and administrative levels, adverse event reporting
Other system requirements	The ZM-EIR follows industry standards for the *data security and privacy* platform *interoperable with* *District Health Information System 2 (DHIS2) and the government’s EPI*^e^ *management information system (MIS)* deployed at *full scale* in all districts of Sindh Province by over 3096 vaccinators at 1694 public and private sector immunization clinics to enroll 3.8 million children and 1.3 million women, with 31 million immunization visits recorded (as of 2021).	Data exchange and interoperability, offline capability, alignment with international standards, data privacy and security, scalability and capacity, usability

^a^ZM: *Zindagi Mehfooz* (*safe life*).

^b^EIR: electronic immunization registry.

^e^QR: quick response.

^d^iDSS: immunization decision support system.

^e^EPI: Expanded Program for Immunization.

### Study Design

The pre-post mixed methods study design measured changes in the proportion of fully immunized children aged 12-23 months from 2014 to 2019. Fully immunized children are those who have received the complete recommended schedule of 5 antigens by 23 months of age. The pentavalent (Penta) vaccine refers to a DPT-Hib-HepB (where Hib-HepB refers to the *Haemophilus* b–hepatitis B conjugate) combination in this context. Additional aims and objectives of the study were assessed through qualitative research (observation and in-depth interviews), secondary data analysis (MICS from 2014 and 2018-2019), and comparison between districts and health facilities with high, moderate, and low ZM-EIR compliance (based on data from the platform or app of log-ins and entries of child encounter information). The results are presented later based on the study aim and objective. Experimental or quasi-experimental study designs were not feasible as the ZM-EIR was implemented throughout Sindh Province and a suitable comparator was not available.

### Patient and Public Involvement

There was no patient or public involvement in the design or implementation of the study. Caregivers, health workers, and health administrators were included in the qualitative research component as key informants after they provided informed consent.

### Ethical Considerations

The study received Institutional Review Board (IRB) approval from the IRD (approval number IRD_IRB_2021_11_001) and was registered with the ISRCTN registry (#23078223). Informed consent was obtained from all key stakeholder groups prior to interviews.

### Data Sources and Analysis

#### Quantitative Data Collection

The 2 sources of quantitative data were the MICS conducted across the state of Sindh, Pakistan, by the United Nations Children’s Fund (UNICEF) from 2014 and 2018-2019 and the ZM registry database. Details of the MICS are provided on the UNICEF website. The indicators across the study aims and objectives are listed in Table 2.

**Table 2:** Indicators included in quantitative analyses.

Indicator	Definition	Numerator	Denominator	Data source
Compliance	The vaccinator updates/uploads 1 record per vaccination day.	Number of days the vaccinator logged in to the ZM^a^ app and uploaded/updated at least 1 child record	Number of vaccination days for that health center/vaccination center	ZM database
ZM registry coverage	N/A^b^	Total number of children receiving any immunization reported in the ZM	Estimated monthly target population of children in Sindh Province derived by the EPI^c^ in close collaboration with the ZM team	ZM child records database
ZM registry use	The proportion of vaccinators trained to use ZM upload details for at least 1 client per day in Sindh Province from 2017 to 2019.	Number of days the vaccinator logged in to the ZM app and uploaded/updated at least 1 child record	Number of vaccination days for that health center/vaccination center	ZM vaccination staff database
ZM dashboard use	This is the number of unique user (manager/supervisor) log-ins to the dashboard.	Number of log-ins per district per year	N/A	ZM management staff usage of dashboard derived from the ZM database
Fully immunized	If the child has received the BCG^d^ vaccine at birth and OPV^e^ 1-3, Penta^f^ 1-3 vaccines, PCV^g^ 1-3, and the measles vaccine 1 at 12-23 months, the child will be considered fully immunized.	Number of children 12-23 months old who received the BCG vaccine at birth, OPV 1-3, Penta 1-3 vaccines, PCV 1-3, and the measles vaccine 1	Sample of children 12-23 months old as part of the survey in Sindh Province	MICS^h^ 2014, 2018-2019
Zero doses	A child aged 6-23 months has not received the first dose of the DPT^i^ 1/Penta 1 vaccine.	Number of children who did not receive the DPT 1/Penta 1 vaccine	Sample of children 12-23 months old as part of the survey in Sindh Province	MICS 2014, 2018-2019
Timely immunization	A child has received the dose of antigen at the recommended age specified in the EPI schedule.	Number of children 12-23 months old who received the BCG vaccine at birth, OPV 1-3, Penta 1-3 vaccines, PCV 1-3, and the measles 1 vaccine as per the timeliness criteria of the Pakistan/Sindh Province EPI	Sample of children 12-23 months old as part of the survey in Sindh Province	MICS 2014, 2018-2019

^a^ZM: *Zindagi Mehfooz* (*safe life*).

^b^N/A: not applicable.

^c^EPI: Expanded Program for Immunization.

^d^BCG: Bacillus Calmette-Guérin.

^e^OPV: oral polio vaccine.

^f^Penta: pentavalent.

^g^PCV: pneumococcal conjugate vaccine.

^h^MICS: Multiple Indicator Cluster Survey.

^i^DPT: diphtheria-pertussis-tetanus.

The ZM platform uses multiple sources to register children, including birth cohort registration, the recording of pregnancies and births, the EPI’s estimated targets, and the date of enrollment. The analysis reflected the vaccine-wise coverage for the age group of 12-23 months. The registry-based coverage was calculated using the enrollment numbers for each calendar year birth cohort as the numerator and the annual targets provided by the EPI (ascertained population based on modeling using birth rates and recent census figures). Furthermore, the target population of children was derived based on the enrollment of children at the time of birth through a birth cohort registration and an EPI schedule–based continuum-of-care mechanism. The numerators were based on aggregate numbers for the different antigens and did not distinguish the age of the child for the receipt of vaccination, which is reflective of immunization data from administrative sources.

We analyzed MICS data using the design considerations for weighting, adjustment of clustering at the level of primary sample units, and strata for survey errors according to guidelines provided by the MICS. We presented survey design–adjusted estimates of vaccine coverage with 95% CIs.

Timeliness was calculated according to the following recommended age schedule. It is worth noting that timeliness for the Penta 3 vaccine does not correct for any minimal interval with previous doses, which makes being untimely almost normal if Penta 1 vaccination is already late (Table 3).

**Table 3:** Timeliness schedule of vaccination.

Antigen and dose	Minimum age	Recommended age	Timely schedule (days)
BCG^a^	Birth	0 weeks/0 days	0-28
Penta^b^ 1/OPV^c^ 1/PCV^d^ 1	6 weeks	6 weeks/42 days	39-70
Penta 3/OPV 3/PCV 3	14 weeks	14 weeks/98 days	95-126
Measles 1	6 months	9 months/274 days	180-302

^a^BCG: Bacillus Calmette-Guérin

^b^Penta: pentavalent.

^c^OPV: oral polio vaccine.

^d^PCV: pneumococcal conjugate vaccine.

#### Qualitative Data Collection

Qualitative data collection was conducted in 3 districts in Sindh Province, selected for the high, medium, and low immunization coverage rates according to the analysis of ZM-EIR system data. Direct observations of immunization services were conducted for 1 day per facility in 6 health facilities and 3 district offices. Within each district, 2 health facilities were selected based on high and low ZM-EIR compliance. At each health facility, 2 vaccinators and 2 caregivers (1 male and 1 female) who were attending the facility for their child’s immunization were interviewed. Selection was based on their availability and consent to participate during field visit days. For each district, the managing supervisor and the district field coordinators and EPI managers were also interviewed to capture views of the leadership, along with vaccinators and clients. A structured discussion guide was used to conduct the interviews, led by a researcher with experience in qualitative methods. Qualitative data were collected from caregivers of children <2 years old (n=12), vaccinators (n=12), supervisors (n=3), district-level EPI managers (n=7), provincial EPI managers (n=2), and ZM-EIR implementation and data teams (n=4). The interviews were audio-recorded and transcribed for qualitative analysis. Direct observations of immunization services were also conducted for 1 day per facility in the 6 health facilities and 3 district offices.

As guided by the Consolidated Criteria for Reporting Qualitative Research (COREQ) checklist, the research team comprised a Pakistani female principal investigator and health system researchers trained in qualitative methods, with master’s degrees in public health and over 10 years of experience. There was no previous relationship with the study participants. District field supervisors facilitated contact with health facilities, and health facilities facilitated contact with caregivers. The sampling was purposive to ensure that all key stakeholder groups engaged in using or having data captured within the ZM-EIR: EPI managers, district health officers, EPI supervisors, vaccinators, and caregivers were included in the sample. Interviews were conducted face to face either in offices/places of work or in health facilities. There were no refusals, and no nonparticipants were present at the time of the interviews. Content analysis was conducted using a framework developed through the content captured within the in-depth interviews. Interview guides were developed and tested prior to data collection. There were no repeat interviews. Saturation was achieved through the structured sampling and interviews conducted. Interviews were approximately 30 minutes in length. They were audio-recorded and field notes captured. Microsoft Excel was used to organize data by theme for ease in the synthesis of findings and reporting. Thematic analysis was conducted using grounded theory through a 2-step process: (1) iterative debriefs conducted concurrently with data collection and (2) coding of transcripts and generation of subthemes and themes. A theoretical framework based on these themes and subthemes was then developed in line with the research study objectives and used to analyze and present findings.

## Results

### ZM-EIR Reach and Use by Providers

Vaccination staff training on the ZM-EIR started in quarter 3 of 2017 as part of a district-wide planning with the Sindh Provincial Department of Health. Data on the proportion of vaccination staff trained on the system from the total were not available. However, all the 1307 staff members nominated for training were trained across 52 batches between September and December 2017. Training batch sizes ranged from 12-40 participants per training session.

The Sindh Provincial EPI defines compliance with the ZM-EIR system by vaccinators as 1 upload or update for at least 1 client per vaccination day. Compliance estimates include instances in which a vaccinator may be present at the health facility but no child is brought in for vaccination. Findings suggested that across 27 districts that were active during the time frame covered by the study, compliance rates ranged from 46% to 85%. The average compliance increased from 40% in 2018 to 57% in 2019. Data are available in Multimedia Appendix 1.

Trends in compliance were assessed based on demographic characteristics of vaccinators. Compliance and usage during 2017-2019 were slightly higher among urban vaccination staff compared to their rural counterparts. Compliance among female vaccinators was higher compared to males, even though the proportion of females in the vaccination staff was only 15%. There was a gradual increase in compliance in all age-groups during 2017 to 2019. Younger age groups (less than 40 years old) showed improvement from 40% in 2017 to 49% in 2019, with the highest proportional increase observed for those aged 26-30 years (49%) and those aged 18-25 years (47%). A more modest increase was found for vaccinators in higher age groups, with the 51-55-year group showing only a 28% increase.

### Stakeholder Perceptions of the ZM-EIR

Stakeholder perceptions of the ZM-EIR were assessed for each set of functions within the ZM-EIR suite of digital health interventions, including the perceived benefits and challenges of use to improve immunization service delivery (Table 4). Overall, all stakeholder groups interviewed had a favorable impression of the ZM-EIR and the associated suite of interventions. Provincial managers felt that the real-time access to vaccination data facilitates longitudinal monitoring of vaccination coverage over time and across geographic areas. They further noted that expansion of the ZM-EIR would allow this to occur across the country as it would enable the provinces to see and evaluate each other’s coverage. The ZM-EIR was thought to provide a blueprint of what a national repository of EPI records could look like and further be a mechanism for improving supply monitoring and fraud prevention. As the primary end users of the ZM-EIR, vaccinators expressed their satisfaction with ZM-EIR features, specifically the use of automatic lists generated of those who may have missed a scheduled vaccine dose or may not have received any vaccines (defaulter and zero-dose lists).

**Table 4:** Key informant general perceptions of the ZM-EIR^a^.

Key informant	Example quotations
Caregiver	“...It is very helpful. Otherwise, we are busy with our work and forget about it. When we receive a message, then we know we have to get the child vaccinated soon.” “The caregivers who used the call center, called it, ‘good in every way’. According to them, these have been proved useful in gaining information about the availability of the vaccination teams, discussing any symptoms caused by the vaccination, and asking for a second dose.”
Vaccinator supervision	“...Wherever he [vaccinator] goes, we monitor it through Google Maps. When we visit a vaccinator, it is a surprise visit. We do not call them. We just follow them on mobile and visit them on field.” “These graphs depict which vaccinators have complied [based on required vaccinations for the day], and which are still noncompliant.”
Manual (paper-based) data capture	“Our people in the field write in the book, on the card, and all that writing is very difficult. It would be better if we just use ZM-EIR, and only 1 thing stays.” “If they had owned it properly, it would end. The vaccinators who work honestly in the field ask us to put an end to the manual data capture work so they can continue working with ZM only.” “All their registers will cease to be used, and all their data will be done on ZM. That will be their daily register and stock register. Work is being done on this, and I think in 1 or 2 months, they may become paper free.”

^a^ZM-EIR: *Zindagi Mehfooz* (*safe life*) electronic immunization registry.

### Registry Coverage

From 2017 to 2019, the ZM-EIR immunization registry enrolled nearly 5.4 million children and 1.9 million married women of reproductive age (15-49 years).

#### Gender-Related Barriers to Registry Uptake

Gender was identified to play a significant role in ZM-EIR uptake by caregivers owing to its embeddedness within the local, rural contexts. There is often a clear distinction between a caregiver’s interaction with male vaccinators and that with female vaccinators. One key informant emphasized the need for female health workers who can bond with female caregivers (who are usually more responsible for taking care of their children than male caregivers are) to resolve any resistance or anxieties regarding vaccinations to further expand immunization efforts. A female caregiver shared:

The female health worker can come inside the house, and we [caregiver] know her, so it is good that a female health worker comes. If a man comes, then it is quite difficult.

#### ZM-EIR Data Use

The provincial managers shared that the ZM-EIR intervention provides defaulter and zero-dose lists, which helps them meet their target and improve performance. It enables those who supervise to encourage the vaccinators to bridge the gaps in the coverage of immunization targets. The data are viewed as reliable due to the feature allowing the system to operate in both offline and online modalities, and the use of the platform is not dependent on the availability of internet services. These data are then communicated to other responsible representatives at the district and provincial levels. Among the stakeholders who can access the data reports and dashboards, the role of supervisors has been seen to be relatively limited compared to others. The supervisor’s overall responsibilities include monitoring and providing support to vaccinators, checking the vaccination stock, and planning and conducting field visits. For their limited use of the ZM-EIR app to monitor the activities of vaccinators in their respective district areas, it was stated that newer and younger supervisors can use the app comfortably, while older supervisors find the app difficult and less engaging. Coordinators and supervisors also suggested that the latter be given access to a separate app to monitor vaccinators so that they do not have to rely on district managers for the data.

#### Observation of Data Entry and Use

Among the health facilities observed, half were clean, organized, and relatively well maintained. The other half were seen as unclean, “cluttered,” and “with broken furniture.” The number of rooms in a health facility ranges from 2 to 7. All the health facilities provide vaccination and other health services. Most of the health facilities have 2 vaccinators present. Of these, 1 (50%) in each facility uses the ZM-EIR for registration and defaulter lists. In all 3 (50%) facilities, it was reported by vaccinators that when the caregivers would bring their children for vaccination, they did not use the ZM-EIR for registration in real time, but they recorded information in the paper-based register system and later entered it into the electronic app. Only 4 (67%) of the 6 facilities had electricity, 1 (25%) being backed by a solar power system. Network connectivity was limited in all facilities, which also hindered the use of the ZM-EIR in real time. All district offices observed had 1 supervisor on-site with access to a computer/laptop in the office, which enabled access to web-based ZM-EIR dashboards. The supervisors reported engaging in daily monitoring of the vaccinators, whereby they tracked the vaccinators’ attendance, location, and registration. No other use of the ZM-EIR was reported or observed (Table 5).

**Table 5:** Key informant views of ZM-EIR^a^ data use by key stakeholder group.

Key stakeholder group	Example quotations
District field coordinators and managers	“When we have their attendance in the morning, the supervisors call right then to ask why this vaccinator isn’t marked present, tell their timing, and all. So, we talk to them daily.” “I can also see everything about defaulter children and why we can’t cover them.”
Supervisors	“[Supervisors] do monitoring by checking their vaccines, by checking their temperature in the monitoring. We check the graph and record to check whether they are taking records or not. We also check whether they [vaccinators] use ZM or not. This is our work.” “We can’t tell if it is useful for the supervisors. Because we don’t know where the vaccinators are. We should be able to check their work through it, even as a guest or monitors.” “All supervisors are old aged. If we make them use it, they won’t be able to. They can’t even see the dashboard, because those poor people aren’t used to it since the start.”
Vaccinators	“The vaccinators are scared because when their defaulters increase, their UCa goes into categorization B or C. So, they are working on the coverage of defaulters, because with ZM, we are getting proper data now. Since the defaulters are being covered to keep their UCs up, that means vaccinations are happening timely.”

^a^ZM-EIR: *Zindagi Mehfooz* (*safe life*) electronic immunization registry.

^b^UC: Union Council.

### Vaccination Coverage and Timeliness

The vaccine-wise coverage across this evaluation’s time frame of consideration (2017-2019) reflected that since its inception for scale-up in the province and phase-wise implementation in all districts, increases were observed for all vaccines (the EPI in Sindh Province had measles 1 as the last vaccine as per the child vaccination schedule till 2018. Subsequently, measles 2 was added as part of the EPI schedule; however, given the evaluation’s considered time frame of 2017-2019, the quantitative analyses focused on measles 1). Findings suggested that improvements in coverage were achieved for BCG (+10.1%), Penta 1 (+8.8%), Penta 3 (+7.4%), and measles 1 (+5.5%) vaccines. Vaccination timeliness was assessed by antigen by comparing the proportion of children 12-23 months old immunized across MICSs in 2014 and 2018-2019. Findings suggested that improvements in timeliness were achieved for BCG (+10.0%), Penta 1 (+3.1%), Penta 3 (+2.0%), and measles 1 (+0.7%) vaccines. However, only the improvement in timeliness for the BCG vaccine was statistically significant (Table 6).

**Table 6:** Vaccination coverage and timeliness in Sindh Province between 2017-2018 and 2014 (MICS^a^).

Vaccination	Prevalence (95% CI)			Increase (%)
	2018-2019 (%)	2014 (%)		
BCG^b^ received	80.2 (78.4-81.9)	70.1 (67.2-72.9)	10.1	
Penta^c^ 1 received	72.5 (70.3-74.6)	63.7 (60.7-66.7)	8.8	
Penta 3 received	50.6 (48.2-53.1)	43.2 (40.0-46.4)	7.4	
Measles 1 received	54.4 (52.1-56.8)	48.9 (45.9-51.9)	5.5	
BCG timely	33.7 (31.6-35.7)	23.7 (21.0-26.4)	10.0	
Penta 1 timely	22.7 (20.8-24.5)	19.5 (17.0-22.1)	3.1	
Penta 3 timely	12.3 (10.9-13.8)	10.4 (8.4-12.3)	2.0	
Measles 1 timely	11.7 (10.3-13.1)	11.0 (9.1-12.9)	0.7	

^a^MICS: Multiple Indicator Cluster Survey.

^b^BCG: Bacillus Calmette-Guérin.

^c^Penta: pentavalent.

Additional analyses were conducted to compare increases in immunization coverage in Sindh Province, along with Punjab Province, which implemented a different EIR as part of its EPI. The analyses indicated increases in immunization coverage, making the increases in immunization coverage and timeliness difficult to attribute to the ZM intervention (Tables 7 and 8).

**Table 7:** Vaccination coverage in Sindh and Punjab Provinces between 2017-2018 and 2014 (MICS^a^).

Vaccination	Sindh Province				Punjab Province		
	2014 (%)	2018-2019 (%)	Increase (%)	2014 (%)		2017-2018 (%)	Increase (%)
BCG^c^	77.4	82.4	5.0	93.1		94.8	1.7
Penta^c^ 1	70.7	75.1	4.4	85.7		92.7	7.0
Penta 3	55.3	57.6	2.3	73.3		87.3	14.0
Measles 1	58.6	62.7	4.1	77.8		82.7	4.9

^a^MICS: Multiple Indicator Cluster Survey.

^b^BCG: Bacillus Calmette-Guérin.

^c^Penta: pentavalent.

**Table 8:** Key informant views of the relationship of the ZM-EIR^a^ to immunization coverage and equity.

Key informant	Example quotation
Immunization program performance	‘…In what [performance] category are [2] UCs^b^, that is, whether [they] are in red, green, blue, or yellow…every UC-wise, every vaccinator-wise, every town-wise, every district-wise, we are getting all the data.”
Immunization coverage	“Because of ZM, the EPI^c^ office has found out that we have 114 vaccinators. If only 67 vaccinators out of them are working, they ask your district where the rest of your vaccinators are. They ask us to enroll them, assign them a field, and send them to work. So now, I have over a hundred vaccinators enrolled, and they work themselves after returning. Coverage will thus ultimately increase.”
Immunization equity	“In outreach, more female children are being vaccinated because they find it easy that they can get the immunization service at their doorstep…we can see [in defaulters list] that more females might be lagging behind; as a result of this, there is catch-up, and we do see better coverage among the female population.”
Immunization timeliness	“…Three SMS reminders are sent so it is not that you only get one SMS reminder for the child’s appointment day…there are these three different time points as a constant nudge to the caregiver to bring the child for immunization at the time that he is required to show up.”

^a^ZM-EIR: *Zindagi Mehfooz* (*safe life*) electronic immunization registry.

^b^UC: Union Council.

^c^EPI: Expanded Program for Immunization.

From a ground-level perspective, vaccinators shared that the selection of local people for authority positions in rural governments has increased the trust of the people in the government, leading local caregivers to trust health authorities and their immunization efforts. In general, the vaccinators also believe that there has been an increase in awareness, coupled with an increase in diseases, which has had a concurrent effect on improved vaccination rates among rural communities.

Managers shared that the timeliness of immunization may be due to the DSS built within the app. Although SMS reminders as a ZM-EIR feature are more caregiver oriented, the DSS within the app integrated as an explicit function is more vaccinator oriented. In instances where vaccinators have not had substantive training, they are likely to get confused about which vaccine to give a child at a specific time.

### Zero Doses

The prevalence of zero-dose children aged 6-23 months, defined as not receiving at least 1 dose of the Penta vaccine, went down from 36.3% to 28.5% (at 95% CI) between 2014 and 2019. Relative decreases in Punjab Province make attribution to the ZM intervention difficult to ascertain.

## Discussion

### Principal Findings

This external evaluation of the ZM-EIR and the associated suite of digital health interventions for childhood immunization in Sindh Province, Pakistan, highlights important insights and recommendations across stakeholder groups related to the benefits of an EIR, namely the availability and use of data through alerts and reminders to caregivers, identification of defaulters, supervision of vaccinators, and overall management of vaccination program activities [2].

Qualitative findings support this assertion; indicating that overall, the stakeholders interviewed had a favorable impression of the ZM-EIR suite of interventions and the perceived benefits of ZM defaulter lists on improved immunization coverage, as well as decreased percentage of zero-dose children. Several suggestions and areas for improvement were made by key informants to address some of the challenges experienced by users of the ZM-EIR.

First, gender dynamics, gender segregation (male vaccinators/female caregivers), education, literacy, and cultural norms impacted access and use by vaccinators and the ability to make direct contact with caregivers. Although their numbers are lower, female vaccinators have higher compliance with the ZM-EIR system than their male counterparts. Power, trust, and relationships were noted to present challenges between vaccinators and clients of the opposite gender. The implementation of the ZM-EIR brings to the fore the deep-rooted gender inequities in society that need to be addressed through relevant policy measures to leverage the full potential of the intervention. In addition, gender analyses at the start of future ZM-EIR intervention planning may also help identify strategies to help overcome this gap. These deeply embedded gendered preferences related to vaccination experiences reinforce the need to incorporate cultural and gender considerations when designing digital health interventions.

Second, we noted reliance on double record keeping, which respondents recommended phasing out to reduce the data entry burden (double requirement to complete both paper and mobile data entry) for field vaccinators and facility staff. Paper-based reporting is a requirement for health program audits. This has led to prioritization of paper reporting over the consistent use of the ZM-EIR for reporting. Our study found the perception that ZM-EIR uptake is hindered by physical record keeping, with a recommendation to transition to paperless reporting.

Although changes in immunization coverage across all antigens, timeliness for the BCG vaccine, and the reduction in the number of zero-dose children cannot be attributed wholly to the ZM-EIR program, the study findings are a promising indication that EIR data availability and use can support improvements in child health outcomes, such as vaccination, possibly through better tracking of clients and availability of data. Small pilot studies in countries such as Vietnam and Bangladesh with immunization programs rolling out EIRs report improvements in key immunization program milestones, such as timeliness of immunization (vaccinations delivered on time according to established schedule recommendations), a reduction in vaccination schedule dropouts, and improved ability for vaccinators to identify and follow-up with defaulters [3,19-22]. Similar to this evaluation, health workers using EIR systems report that they use the data to identify defaulters, coverage disparities, and vaccine stock levels and that they have more confidence to take action based on these data analyses [3]. However, this is the first known study of this degree of comprehensiveness, scale, and rigor to evaluate the availability and use of data of an EIR and the potential for associated effects on immunization outcomes.

### Limitations of the Study

The study was a retrospective one, with a small sample of qualitative interviews, and information bias due to recall cannot be excluded. Stakeholders may also have expressed a positive view of the program due to reasons of social desirability. The MICS estimates show similar changes in Pakistan’s Punjab Province, which was not a target of the ZM intervention. But since immunization has been at the forefront of public health issues in Pakistan, similar programs were likely in place in Punjab Province. These create challenges in the attribution of effects to programs such as ZM. ZM did not act in isolation but would have been greatly affected by the presence of other health system–strengthening activities, including improvements in supply chain logistics, training and supervision of providers, and demand generation activities targeting immunizations. The increase in immunization coverage may also be attributed to more structural factors (eg, public trust in the government).

### Conclusion

This study contributes to the evidence base of the added value of increased data availability and use through the implementation of EIRs in low- and middle-income countries as part of broader EPI interventions. It aligns with key benefits identified in other studies of immunization information systems and EIRs, namely alerts and reminders, missed vaccinations, supervision and oversight of vaccinators, and management and accountability. Study methods included a retrospective analysis of system-generated data, qualitative stakeholder interviews, and assessment of potential contributions to improvements in immunization coverage and timeliness and reduction in the number of zero-dose children. Findings suggest that the ZM-EIR is a promising technology platform that may have contributed to improvements in the coverage and timeliness of BCG vaccination, as well as the reduction in the number of zero-dose children as part of a broader package of immunization interventions in Sindh Province, Pakistan, from 2017 to 2019.

## Appendix

Multimedia Appendix 1 Zindagi Mehfooz Evaluation Comprehensive Report.

## Abbreviations

AI: artificial intelligence
BCG: Bacillus Calmette-Guérin
DPT: diphtheria-pertussis-tetanus
DSS: decision support system
EIR: electronic immunization registry
EPI: Expanded Program for Immunization
iDSS: immunization decision support system
MICS: Multiple Indicator Cluster Survey
OPV: oral polio vaccine
Penta: pentavalent
QR: quick response
UC: Union Council
ZM: Zindagi Mehfooz (safe life)

## Data Availability

A comprehensive report of all quantitative and qualitative data analyses conducted as part of the study has been included as Multimedia Appendix 1.
