# Supplementary material for: Evaluating the “Zindagi Mehfooz” Electronic Immunization Registry and Suite of Digital Health Interventions to Improve the Coverage and Timeliness of Immunization Services in Sindh, Pakistan: Mixed Methods Study
Source: J Med Internet Res. 2024 Oct 11;26:e52792. doi: 10.2196/52792 (PMC11512122; doi:10.2196/52792)

January 2023

Evaluating the effectiveness of Zindagi Mehfooz Electronic Immunization Registry: A suite of digital health interventions, to improve the coverage, timeliness, and completeness of immunization services in Pakistan

Draft report prepared by Patricia Mechael, Sara Gilani, Ahsan Ahmad, Amnesty LeFevre, Diwakar Mohan, Asra Memon, and Michal Waga

With inputs from Subhash Chandir, Vijay Kumar Dharma, Bilal Ghani, Sundus Iftikhar, Mubarak Taighoon Shah, Danya Arif Siddiqi, Riswana Soundardjee

Table of contents

**1 EXECUTIVE SUMMARY**	**4**
**2 BACKGROUND**	**5**
**3 ZINDAGI MEHFOOZ SYSTEM OVERVIEW**	**6**
**4 ZINDAGI MEHFOOZ-EIR THEORY OF CHANGE**	**7**
**5 EVALUATION OVERVIEW**	**8**
5.1 Evaluation Setting	8
5.2 Data Sources and Analyses	8
**6 RESULTS BY STUDY AIM AND OBJECTIVES**	**11**
6.1.1 Vaccinator Use by Area, Sex, Age, and Education	11
6.1.1.1 Contextual considerations of the ZM intervention	14
6.1.1.2 Profile of ZM-EIR Users	14
6.1.1.3 General perceptions of ZM intervention	14
6.1.1.4 Vaccinator profile	14
6.1.1.5 Perceived Utility of ZM	14
6.1.1.6 Implementation model	15
6.1.1.7 Perceived barriers to successful implementation of ZM-EIR	15
6.1.1.8 Caregiver Experiences with ZM-EIR: Awareness and Experiences	16
6.1.1.9 Women's access and use of mobile phones	16
6.1.1.10 Recommendations from Caregivers	16
6.1.1.11 Perceptions related to the impact of ZM-EIR on immunization coverage	18
6.1.1.12 Perceptions related to the impact of ZM-EIR on immunization equity	18
6.1.1.13 Data use for immunization service delivery	19
6.1.1.14 Data use for supervision	20
6.1.1.15 Data use for immunization system performance assessment	20
6.1.1.16 Recommendations to improve data use	20
6.1.1.17 ZM-EIR expansion to other regions	21
6.1.2 Hib- H. influenzae type b; HepB-Hepatitis B	27
**7 DISCUSSION**	**29**
7.1 Overall Perceptions & Experiences with ZM-EIR by Stakeholder Group	29
7.1.1 Caregiver Perceptions & Experiences with ZM-EIR	29
7.1.2 Vaccinator Perceptions & Experiences with ZM-EIR	29
7.1.3 Management Perceptions & Experiences with ZM-EIR	29
7.2 ZM-EIR as More than an EIR	30
7.2.1 State of EIR Evaluations	30
**8 RECOMMENDATIONS**	**32**
**9 CONCLUSION**	**33**
**10 REFERENCES**	**34**
**11 ANNEXES**	**35**
11.1 Annex A: Study Aim and Objectives	35
11.2 Annex B. Immunization schedule in Pakistan and definition of full basic immunization	36
11.3 Annex C: Definitions for examining Routine Immunization Timeliness	37
11.4 Annex D. District Selection for Qualitative study	39
11.5 Annex E. Training of vaccination staff on ZM-EIR application – Sept-Dec 2017	40
11.6 Annex E: DISTRICT LEVEL ANALYSES	42

List of figures

Figure 1. Overview of ZM System 6

Figure 2. Map of Sindh province and Karachi, by district 9

Figure 3. Provincial level compliance of ZM usage of the vaccination staff stratified by Urban -Rural and Sex (2017-2019) 12

Figure 4. Provincial compliance of vaccination staff according to age group for 2017-2019 12

Figure 5. Provincial compliance vaccination staff according to education status for 2017-2019 13

Figure 6. Number and percentage of HR logins stratified among supervisory and management staff cadres of EPI programs 19

Figure 7. Vaccination coverage among children 12-24 months of ages in Sindh from 2014 MICS -2019 MICS 21

Figure 8. Proportion of children 12-23 months in Sindh who received timely immunization by antigen in 2014 and 2019 MICS survey 22

Figure 9. Proportion of lives saved by antigen in 2018 26

List of Tables

Table 1. Data Sources for Each Study Aim 8

Table 2. Qualitative Research Study Population and Sample 9

Table 3. Summary Terminology and Measurement 10

Table 4. Proportion of vaccinators by district and year who upload at least one child record per vaccination day [October 2017-December 2019] 11

Table 5. Proportion of children 12-23 months contained within the ZM Registry from 2018-2019 17

Table 6. Prevalence of full immunization among children 12-23 months across two rounds of MICS by socio-demographic characteristics 23

Table 7. Prevalence of zero dose among children 12-23 months across two rounds of MICS by socio-demographic characteristics 24

Table 8. Unadjusted Start-up and On-going implementation support costs from October 2017 to December 2018 in 2019 USD39 25

Table 9. Incremental changes in coverage by antigen 2014 MICS-5 and 2019 MICS-6 26

Table 10. Incremental lives saved from 2017-2019 among children 12-23 months in Sindh 27

Table 11. Annualised program costs (2019 USD) and incremental lives saved for 10 months (March 1, 2018 to December 31, 2018) of implementation across Sindh 27

Table 12. League table comparing cost per life saved with alternative resource uses 28

Table 13. Comparison of ZM and ideal EIR characteristics and functionalities 30

1. Executive summary

There is growing interest and investment in Electronic Immunization Registries (EIRs) with the increasing digitalization of health systems worldwide. The Zindagi Mehfooz (Safe Life) Electronic Immunization Registry (ZM-EIR) in Sindh Province, Pakistan is a comprehensive suite of digital health interventions which aims to improve equitable access, timeliness, and coverage of child immunizations through a smartphone-based application (app) for vaccinators, web-based dashboards for supervisors and managers, and a text message reminder system and call centre for caregivers. It is a registry that enrols women and all new-borns or never vaccinated children with a unique ID/QR code assigned to each woman and child. Although originally implemented as an EIR, over the years ZM has evolved as a suite of digital health interventions that goes beyond an EIR when assessed against standard features of EIRs as documented by PATH (2021) and PAHO (2017). In 2012-13, only about half (58.3%) of children (12-23 months) received all basic vaccinations (PDHS 2012-13) in Pakistan, with marked geographic variation across the country; Sindh province had a full immunization coverage of 35% (MICs, 2014). This report documents the impact of ZM-EIR on increasing the proportion of children 12-23 months fully immunized in Sindh province through the increased availability and use of data and provides further evidence on the impact and cost-effectiveness of ZM-EIR, which serves to increase the knowledge of the benefits of EIRs as part of a suite of digital health interventions, further contributing to the evidence base related to digital health. ZM-EIR aims to strengthen the EPI service delivery and health system through addressing the problem of low and delayed coverage that leaves children susceptible to vaccine-preventable diseases.

SUMMARY OF RESULTS

- Average compliance among vaccinators using ZM-EIR increased year on year from 40% in 2017 to 57% in 2019 with highest compliance at 85% and lowest at 37% (attributed to province-wide vaccinator strikes).
- ZM-EIR implementation in Sindh Province contributed to a 10-percentage point increase in the prevalence of fully immunized children, from 5% before implementation to 15.1% of 12-to-23-month children fully immunized after 3 years of implementation.
- The rate of on-time vaccination (timeliness) was achieved for BCG (+8.3%), Penta I (+3.2%) and Measles II (+1.3) vaccines.
- The prevalence of zero-dose children (defined as child aged 6 to 23 months that has not received the first dosage of DPT1/Penta) saw a decrease from 38.7% pre-implementation to 24.7% after implementation.
- Better coverage in higher wealth quintiles, better educated and older mothers and for male children.
- ZM-EIR implementation in Sindh Province from 2017 to 2019 is estimated to have saved over 3,000 lives among children 12 to 23 months through the more efficient delivery of timely and complete childhood vaccines.
- The estimated cost to implement this suite of tools is $1,362 USD per life saved and $50.45 per DALY averted.

OVERALL PERCEPTIONS

All stakeholder groups interviewed had a favourable impression of ZM-EIR and the associated suite of interventions.

- Provincial managers stated that the real time access to vaccination data facilitated longitudinal monitoring of vaccination coverage over time and across geographic areas.
- District managers highlighted importance of vaccinator tracking and attendance monitoring, coverage tracking, zero-dose, and defaulters as well as daily and monthly reports used to facilitate interaction with vaccinators and other EPI stakeholders.
- Vaccinators expressed their satisfaction with ZM features, specifically for defaulter and zero-dose lists. Vaccinators use the app daily beginning with marking their attendance as one of the first tasks.
- Caregivers found the reminders and the ability to call the helpline to ask questions useful. However, they highlighted some gender-related barriers of female caregivers not wanting to give their phone numbers to male vaccinators.

KEY TAKE-AWAYS AND RECOMMENDATIONS

- Gender dynamics, gender segregation (male vaccinators/ female caregivers), education, literacy and cultural norms impact access and use by vaccinators & provision of phone numbers by caregivers.
- Power, trust and relationship challenges between vaccinators and clients of the opposite gender. Limited female vaccinators have higher compliance than male counterparts. Recommendation to engage more female vaccinators.
- Increased record-keeping burden for vaccinators (paper + mobile app). Recommendation to move towards paperless system.
- Key gap in use is among supervisors who are older and less able to use the computer-based system. Recommendation to co-design supervisor app for mobile phone and/or tablet.
- Limited internet connectivity, electricity and mobile network coverage at health facilities and outreach locations. Recommendation to engage with Ministry of ICT and MNOs to improve connectivity in poor performing districts.

Overall, ZM is a good demonstration of a platform that addresses zero-dose and under-immunized children with an integrated digital health intervention approach to immunization that combines demand generation, geo-spatial data, stock management, effective sub-national data use by Provincial and District Managers and decision support and case management for service delivery by vaccinators. The learning curve, experimentation, and start up investments

in ZM will likely lead to more cost-effective implementation of ZM and similar platforms in the future in Pakistan and elsewhere. Enablers such as capacity, connectivity and electricity should be prioritized in digital health intervention

scale up. Gender-related effects identified in the use of ZM and in its uptake by caregivers should be addressed more systematically through gender analyses and planning at the start of all future digital health intervention planning. The results of this study will be presented to the Ministry of Health of Sindh and the national Ministry of Health in Pakistan to inform decisions on investment in ZM and EIRs in Pakistan and elsewhere. They will also further inform planning for implementation of systems supporting large-scale immunization programs by Gavi, the Vaccine Alliance, globally.

2. Background

In the last two decades, digital health interventions have proliferated rapidly and are currently being used by frontline health workers (FLHWs) in a range of settings to support service delivery, including decision-support, workflow planning, and data capture (Singh et al., 2021). Limited, but emerging evidence suggests that digitally enabling FLHWs may help to improve the content, quality, and timeliness of health services for maternal and child health. In the context of immunization services, direct to beneficiary services which send alerts and reminders to caregivers have additionally been shown to improve immunization coverage and timeliness (Gibson et al., 2017). Despite their immense potential, few digital health interventions have successfully scaled, and among those that have, the pathway to ensuring long-term sustained implementation remains uncertain (LeFevre et al., 2021; Singh et al., 2021).

There is a paucity of available evidence on the impact of digital health interventions often due to limited time and resources. The rapid pace with which technology can evolve is often seen in conflict with the one-to-three-year timelines of impact evaluations. For many, preference is given to rapidly promoting scale-up and limiting the importance placed on evidence given that many digital solutions are bolstering access to public health interventions with known efficacy. However, increasingly, the absence of evidence is seen as barrier to securing long-term funding from governments and transitioning from reliance on donor support (Swartz et al., 2021). Particularly in the context of suites of digital interventions implemented across heterogenous populations of beneficiaries, health workers, and health systems, evidence on the impact of the individual parts as well as the overall program can inform understanding of what works and what may require optimization. In Pakistan digital health interventions have been used to bolster a range of supply and demand side health interventions. Direct to beneficiary solutions have sought to improve medication compliance in patients with non-communicable diseases, and several telemedicine initiatives have sought to enable female doctors to practice remotely (Kazi et al., 2020). Among FLHWs examples of digital solutions are emerging, however, few solutions have scaled widely (Kazi et al., 2020).

The implementation of Zindagi Mehfooz (Safe Life) Electronic Immunization Registry (ZM-EIR) in Sindh Province, Pakistan is one exception. ZM-EIR is a comprehensive suite of digital health interventions which aims to improve equitable access, timeliness, and coverage of child immunizations through a smartphone-based application (app) for vaccinators, web-based dashboards for supervisors and managers, text message alerts and reminders for caregivers, and a call center.

ZM-EIR aimed to strengthen the EPI service delivery and health system through addressing the problem of low and delayed coverage that leaves children susceptible to vaccine-preventable diseases. In 2012-13, only about half (58.3%) of children (12-23 months) received all basic vaccinations (PDHS 2012-13) in Pakistan, with marked geographic variation across the country; Sindh province had a full immunization coverage of 35% (MICs, 2014) and lagged behind Punjab (56%) in improving routine immunization coverage and timeliness. Against this backdrop, ZM-EIR was designed to address supply and demand side barriers that led to underutilization or sub-optimal utilization of immunization services. On the supply side, there were problems associated with the inability to track immunization history of children as well as the inability to assess catch-up immunization schedules, lack of motivation among health workers, the excessive burden of paperwork and poor reporting, management, supervision, and monitoring of vaccinators. On the demand side challenges were associated with low uptake of immunization services due to a lack of awareness and motivation among parents as well as inability to remember vaccine appointments.

Since its inception in 2012, ZM has been scaled across 30 districts of Sindh Province (as of August 31, 2022) and has been used by over 5,071 vaccinators at 1,533 public and 251 private sector immunization clinics to enroll 7,000,837 million children, and 2,482,355 million women with 79,323,041 million (75,408,160 children & 3,914,881 women) immunization visits recorded. It has also been deployed in Gigit (July 2019), Islamabad (August 2022), and KP Swabi district (June 2022).

This report summarizes findings from an external evaluation of ZM-EIR funded by Gavi, The Vaccine Alliance and led by HealthEnabled in collaboration with a team of local and global researchers with the lead research group Gallup Pakistan, who supported the primary data collection for this study.

The goal of the evaluation is to document the impact of ZM-EIR on increasing the proportion of children 12-23 months fully immunized in Sindh province through the increased availability and use of data. This report presents preliminary findings by evaluation aim and objectives (listed in Annex 1) and will be complemented by a peer-reviewed publication.

3. Zindagi Mehfooz System Overview

ZM-EIR is a suite of digital health interventions, centered around a mobile phone-based registry. ZM-EIR is comprised of complementary interventions including:

- Web-interface for data visualization, mobile based data entry and access for vaccinators
- Geo-spatial data for vaccination events
- 2-way interactive SMS reminders for parents/caregivers
- Call center to answer queries of parents/caregivers
- Offline mode to work in areas of poor connectivity
- Child registry for enrolling newborns or never-vaccinated children
- Immunization decision support system (iDSS) to guide vaccinators on routine and catch-up immunizations
- Defaulter reports for vaccinators
- Gamified videos for training vaccinators
- AI-based predictive analytics to identify children highly likely to drop out
- AI-based chatbot to address caregiver queries and concerns in real-time
- GSM-based tracking of vaccinators during work hours
- QR code-based tracking for unique identification of children and women

The android application is used to capture data when beneficiaries arrive at primary health care facilities for immunization services. The child's biodata, including the name, address, and date of birth are filled on the Expanded Program for Immunization (EPI) card and entered into the application at the time of enrolment linked to a QR code[1]. During follow-up visits, the child's ID can be retrieved by scanning the QR code, data are retrieved from the server giving the vaccinator access to individual child's vaccine schedule. The child is then vaccinated and their data on services received inputted into the ZM app which is submitted to the server. To remind caregivers of when they need to next visit the clinic, text message reminders are sent. Up to 3 automatic personalized SMS reminders are sent with the following schedule: a day before, on the day of, and six days after the scheduled immunization date (if the child failed to show up for his/her appointment). A child is considered to have completed the full course of immunizations after the second dose of measles. All data inputted into the ZM app are collated on a central web-based dashboard which supervisors can use to monitor progress, identify defaulters, and take corrective action to achieve targets.

FIGURE 1. OVERVIEW OF ZM SYSTEM

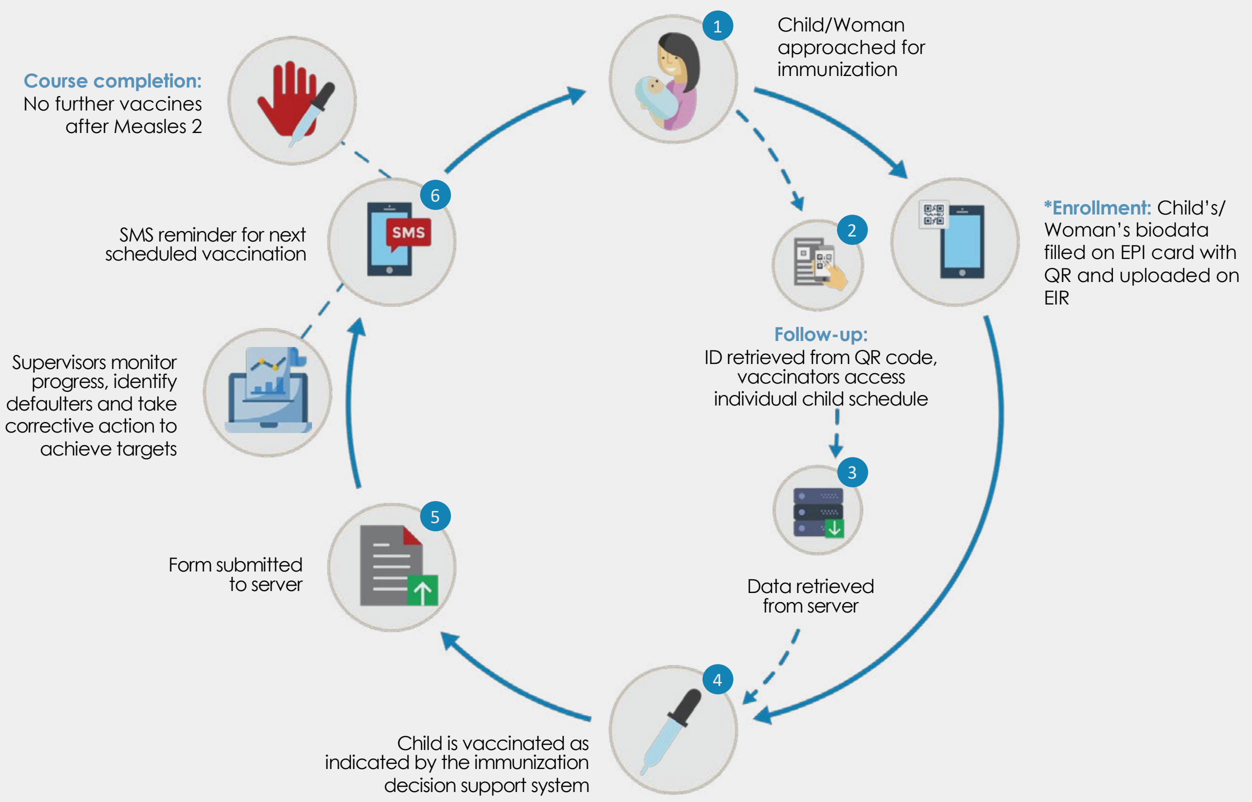

1 QR Code is a two-dimensional version of the barcode, typically made up of black and white pixel patterns

4. Zindagi Mehfooz-EIR Theory of Change

The following theory of change for ZM-EIR depicts program activities in green by level of the health system from beneficiaries, providers, managers, implementation support from the program, and district/ provincial level. Program activities lead to outputs, outcomes, and ultimately impact – depicted in varying shades of pink to red. Assumptions underpinning the movement between boxes are highlighted in yellow.

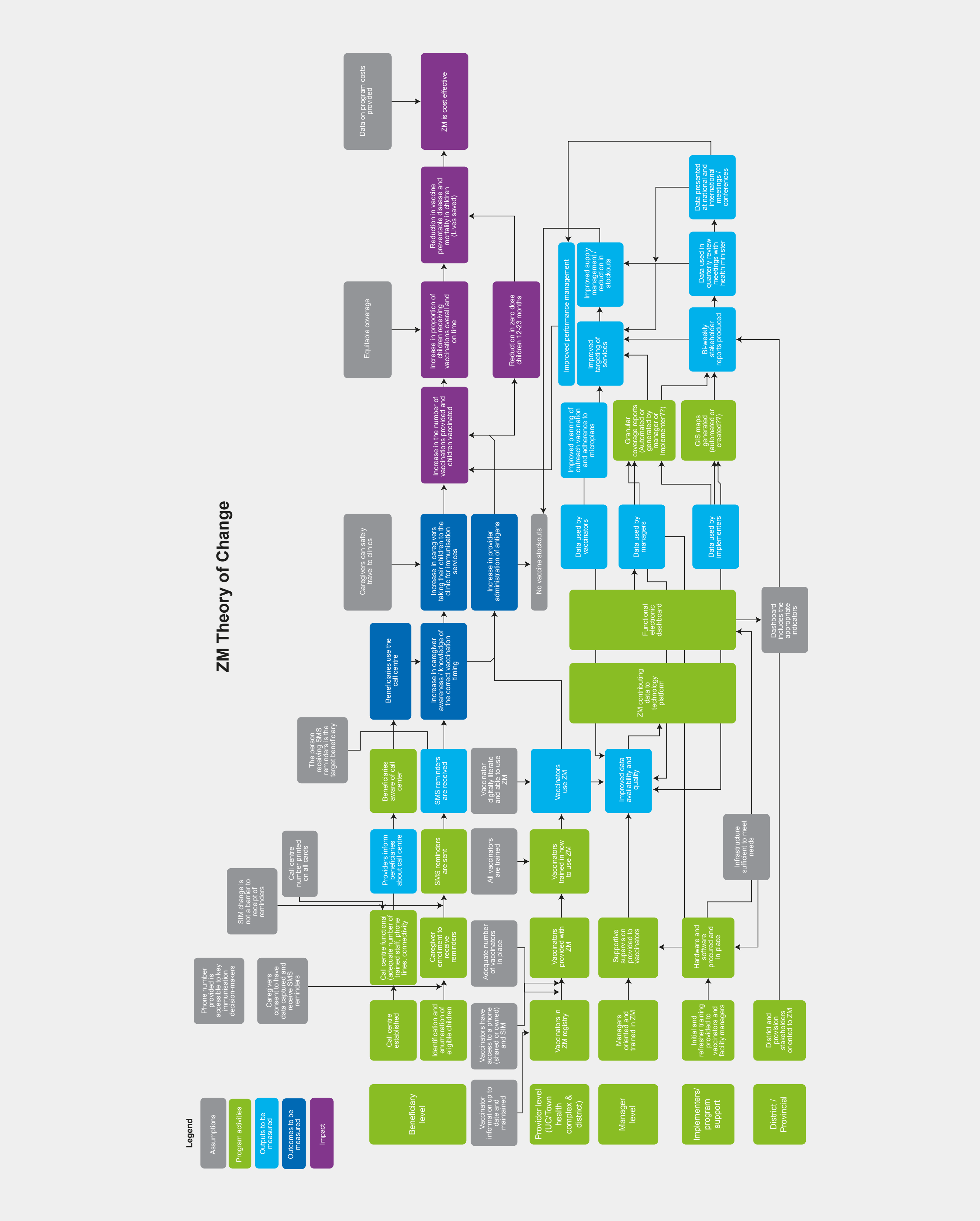

5. Evaluation Overview

Program evaluation activities sought to draw on qualitative and quantitative research methods. As part of quantitative research activities, data on immunization coverage were drawn from nationally representative cross-sectional surveys collected at two time points (prior to the start of the intervention and during the intervention) and used to estimate changes in immunization outcomes. Experimental or quasi-experimental study designs were not feasible given that implementation had occurred throughout Sindh province and a comparator was not available. The study received IRB approval from the IRD Review Board with number – IRD_IRB_2021_11_001 and is registered with ISRCTN #23078223.

5.1 EVALUATION SETTING

Home to over 221 million people, Pakistan is the fifth most populous country globally. Pakistan is a federation of four provinces, the second most populous of which is Sindh. The ZM-EIR program is currently being implemented across all of Sindh's 29 districts[1]. Sindh is located in south-east Pakistan. The provincial capital, Karachi (population 18 million), contains two of the countries busiest seaports and is Pakistan's largest capital city and economic hub. Sindh's population, like much of Pakistan, is characterized by wide gender (male/female), (urban/rural) geographic and socioeconomic disparities. These considerations have been accounted for as part of the evaluation design and included in the presentation of findings.

The administrative set-up for immunization is structured in such a way that the vaccinators engage with caregivers to provide vaccination services. Supervisors are responsible for managing and leading the vaccinators and are based at the District Health Office (DHO). District EPI Managers provide overall oversight for the EPI Program and engage in the use of ZM data for EPI performance monitoring, problem identification and corrective action and use this data to engage with the Provincial EPI Managers. The structure also includes District Field Coordinators who, as ZM-EIR staff, troubleshoot issues with ZM-EIR that arise for or with supervisors and vaccinators within that district.

5.2 DATA SOURCES AND ANALYSES

Data sources have been mapped to each study aim of the evaluation and included in Table 1. Primary qualitative data collection was conducted with the sample as detailed in Table 2. In addition, the secondary quantitative analyses have been outlined in Table 3. A more detailed description of the data and analyses is provided below.

Primary data from **qualitative research** were used across multiple study objectives. Thematic analysis was conducted using Grounded Theory through a two-step process: 1) iterative debriefs conducted concurrently with data collection and 2) through coding of transcripts and generating sub-themes and themes. Translated and transcribed interview notes were recorded in Excel and organized by questionnaire domains/ headings with participant responses depicted in columns. A codebook was then developed wherein codes were entered for the participant responses. Coding was carried out independently by two researchers on all transcripts. It was then mutually agreed upon through reviews and discussion. Subsequently, themes and sub-themes were deduced from the codes generated for the transcripts. A theoretical framework based on these themes and sub-themes was then developed in line with the research study objectives. This has been used to analyze and present the findings.

TABLE 1 DATA SOURCES FOR EACH STUDY AIM

	Study aims	Data source
1	ZM-EIR Initiation: barriers and facilitators	In-depth interviews with vaccinators, clients, and supervisors<br><br>System generated data: Provider profile, app use<br><br>Program records on training
2	Registry coverage	System generated data: ZM-EIR<br><br>In-depth interviews with vaccinators, clients, and supervisors
3	Data use	System generated data: ZM-EIR reports and dashboards<br><br>In-depth interviews with key stakeholders: Union Council, district, and provincial levels, ZM-EIR staff<br><br>Direct observations of immunization services in selected facilities
4	Impact on immunization	MICS-5 2014 and MICS-6 2018-2019
5	Equity	MICS-5 2014 and MICS-6 2018-2019
6	Cost effectiveness analysis	ZM-EIR financial data<br><br>MICS coverage data inputted into the Lives Saved Tool

Programmatic reach (Study aim 1) encompassed dimensions of program reach, provider use of ZM-EIR, and stakeholder perceptions of the intervention. Program reach was assessed based on program training records and defined in terms of the number of vaccinators trained by the program. System generated data on provider engagement with the ZM-EIR app and manager use of the data visualization dashboards were used to assess provider use of ZM-EIR system and data.

In-depth interviews (n=40) with vaccinators, caregivers, supervisors, and managers were conducted to document perceptions about the benefits and challenges of ZM-EIR and perceived effects on immunization service delivery (decision-support algorithm, registry, SMS alerts and reminders). Sampling for the qualitative interviews drew from system generated data to identify districts with high use of ZM-EIR and high immunization coverage, low use of ZM-EIR and better performing and poorly performing districts was based on a combination of ZM-EIR database analysis of "compliance" and Measles I coverage and timeliness for immunization program performance. Compliance is defined by EPI Program as vaccinator uploads/updates at-least one child record in the ZM-EIR application per eligible vaccination day.

The **immunization program performance** criteria for the year 2018 (the mid-year for evaluation's duration of interest) were used to rank compliance with ZM-EIR across all districts. Based on this ranking, the list of districts was categorized into equidistant high, middle, and low compliance segments through ZM-EIR data-based categories rather than arbitrary cut-off points. Overall, these compliance levels were also associated with high, moderate, and low immunization coverage. District selection took into consider high/mid/low immunization coverage and registry coverage with the latter serving as a barometer of vaccinator ZM-EIR app use. Rankings from the criteria were compared to identify two common districts in the high compliance, moderate

1 In 2018-2019 Sindh was administratively apportioned into 29 districts (an increase from 27).

FIGURE 2. MAP OF SINDH PROVINCE AND KARACHI, BY DISTRICT

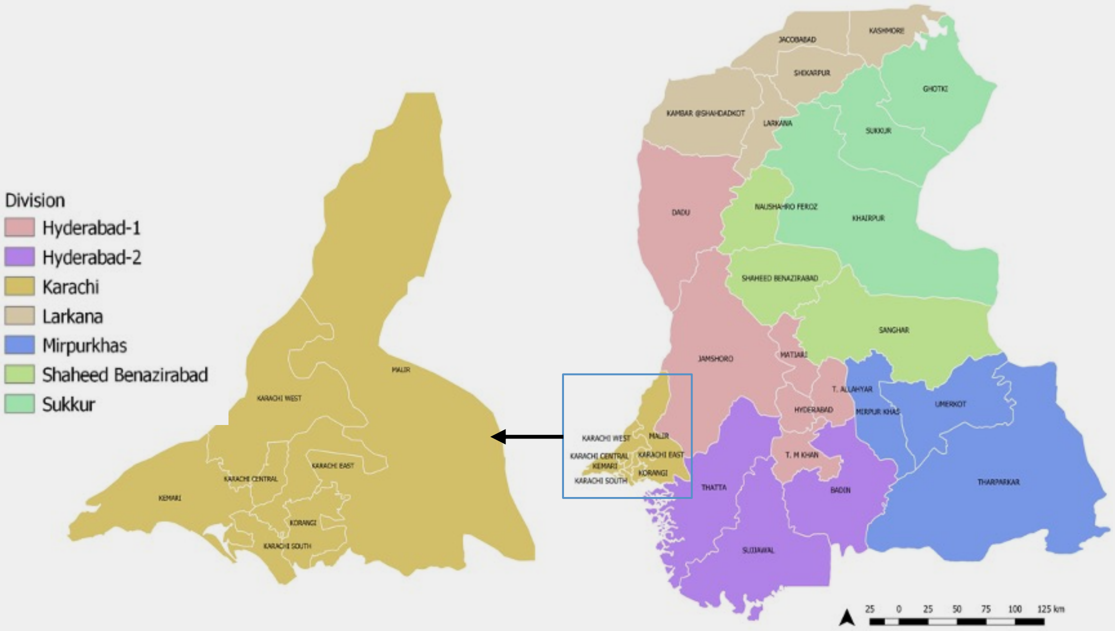

compliance, and low compliance category across all criteria. These districts were recommended for qualitative data collection (See Annex). In consultation with IRD, the following three districts were selected for the qualitative research: Jacobabad (Lower third), Shikarpur (Middle third), and Noshero-Feroz (Upper third). This provided a good cross section of contexts to explore overall user satisfaction and experiences with ZM to identify both positive experiences as well as challenges.

Two facilities per district were randomly selected: one from a high performing Union Council (UC) and one from the low performing UC. Within each health facility, two vaccinators were randomly selected for in-depth interviews. Two caregivers/ clients who had brought their children for immunization were also randomly selected for interview (one male and one female) at each facility. Most interviews were conducted in-person and ranged from 60 to 90 minutes in length. All interviews were conducted in Urdu and/or Sindhi language (depending on the preference of the respondent), audio recorded, and translated into English and transcribed. No remuneration was offered to the participants. All interviews were conducted after obtaining informed consent. Table 3 summarizes the final sampling by key stakeholder group.

ZM-EIR coverage was used to determine the proportion of eligible children at a population level contained with the ZM registry. System generated data on the number of children who have biodata entered into the ZM app served as the estimated number of children reached by vaccinators and the program. Univariate logistic regression models were used to predict the registry's coverage by considering age of the child, gender of the child, residence, ethnicity, and mother's education as independent variables. Crude odds ratio along with 95% confidence interval were estimated. The independent variables which were significant at $p<0.25$ were moved to the multivariate model. For multivariate logistic model, a level of significance was set at 0.05. Adjusted odds ratio along with 95% confidence interval were then estimated.

ZM-EIR data use (Study Aim 3) was assessed by analyzing

TABLE 2 QUALITATIVE RESEARCH STUDY POPULATION AND SAMPLE

Study population	Description	In-depth Interviews & Observations
Caregivers of children < 2 years	Individuals who come into facilities to have their children vaccinated	2 per facility<br>N=12 caregivers (6 male and 6 female)
Vaccinators	FLHWs whose job description includes vaccination and who have been trained to use ZM as part of immunization service delivery activities	N=12 vaccinators (6 from high performing UCs (defined as xxx) and 6 from low performing UCs) (defined as xxx)<br>6 selected health facilities were observed
Supervisors	Supervisors of vaccinators who are working in selected facilities	N=3 supervisors. The 3 selected supervisors were observed at their district offices
District level EPI Managers/ personnel	District managers in selected districts responsible for the EPI program and EPI-related data management and use	N=7 district managers<br>(4 District Focal Persons EPI and 3 District Field Coordinators
Provincial EPI and/ or Data focal person	Provincial leadership responsible for EPI Program and/or EPI-related data use	N= 2 Provincial representatives of EPI
ZM-EIR implementation and data teams	ZM-EIR leadership and staff familiar with start-up and implementation of ZM-EIR and use of ZM-EIR data	N=4 ZM-EIR representatives, managers, implementers, trainers, data team representatives

system generated data on the ZM-EIR registry and dashboards from 2017 to 2019. System use by vaccination staff was measured as a function of any (≥1) log-in attempts to enter a child record per vaccination activity day across a calendar year. Data use by management staff was defined based on the number of log-ins to the ZM-EIR Dashboard. It was derived on a yearly basis of each district for the years 2017, 2018 and 2019 to ascertain the patterns of data usage by the supervisory and management staff cadres of the immunization program and the district and provincial health department personnel of Sindh province.

Impact of the program on **vaccination coverage** was assessed using the estimates from MICS survey before and after the implementation (2014 & 2019). Crude estimates were derived after accounting for the survey design elements such as clustering and stratification with the primary sampling unit (PSU) being the enumeration area as defined by the survey documentation. To account for changes over in the distribution of baseline characteristics associated with probability of vaccination, we used multivariable logistic regression models. The variables accounted for in the models included the wealth quintile of household, education of the mother and father, urban-rural residence, and number of children in the household. The intervention effect estimate was the coefficient for the survey round variable (from equation below) with dummy values 0 for 2014 and 1 for 2019.

$$\text{Logit}(Y_{ij}) = b_0 + b_1 * \text{intervention} + b_{2...n} * \text{baseline variables}$$

Where Y_{ij} is probability of vaccination for the ith child in the jth PSU, b_1 is the intervention estimate (program effect) and $b_{2...n}$ coefficients for other control variables.

All analyses are presented with 95% Confidence intervals. The analyses were performed using Stata version 17 (statacorp).

To assess changes in equity, we estimated the prevalence of fully immunized and zero-dose children across a range of socio-demographic variables like wealth, urban/rural residence, education of mother and head of household, mobile phone access and sex of the child. Survey design adjusted proportions were calculated for each round of the MICS survey (2014 & 2019) with Huber-White standard errors to account for design effect. All estimates are presented with 95% confidence intervals.

Cost-effectiveness analysis: Incremental costs were divided by incremental health effects to generate a deterministic estimate of the incremental cost-effectiveness ratio (ICER), expressed as a cost per life saved. To test for uncertainty, we conducted one-way and probabilistic sensitivity analyses performed in Microsoft Excel using a Monte Carlo simulation with 1000 iterations per analysis. The resulting mean point estimate was obtained by dividing mean costs by mean effects. The 95% CI for the ICER was presented based on percentiles. A cost-effectiveness plane and cost-effectiveness acceptability curve were used to calculate the probability that the intervention would be cost-effective for each of the several standard thresholds of cost-effectiveness. A GNI threshold of $1,620 was used to assess cost-effectiveness, in keeping with established standards set by the Commission for Macroeconomics and health and World Health Organization to determine cost-effectiveness.

TABLE 3 QUALITATIVE RESEARCH STUDY POPULATION AND SAMPLE

Indicator	Definition	Numerator	Denominator	Data source
**Compliance**	Vaccinator updates/uploads 1 record per vaccination day	Number of days the vaccinator logged in the ZM app and uploaded/updated at-least one child record	Number of vaccination days for that health center/vaccination center	ZM database
**ZM registry coverage**		The total number of children 12-23 months receiving any immunization reported in the ZM	Estimated monthly target population of children of Sindh Province that is derived by the EPI program in close working/collaboration with the ZM team	ZM child records database
**ZM registry use**	The proportion of vaccinators trained to use ZM who upload details for at least 1 client per day in Sindh from 2017 to 2019	Number of days vaccinator logged in the ZM app and uploaded/updated at-least one child record	Number of vaccination days for that health center/vaccination center	ZM vaccination staff database
**ZM dashboard use**	The number of unique user (Manager/ Supervisor) log-ins to the dashboard	Number of log-ins per district per year		ZM management staff usage dashboard derived from ZM database
**Fully immunized**	Child has received BCG at birth, OPV 1, 2, and 3, Penta 1, 2, and 3, Pneumococcal 1, 2, and 3, and Measles 1 in 12-23 months old children, then child will be considered as fully immunized	Number of children 12-23 months who received BCG at birth, OPV 1, 2, and 3, Penta 1, 2, and 3, Pneumococcal 1, 2, and 3, and Measles 1	Target number of children (as per EPI program) per district and per year for 2017-2019	MICS 2014, 2019
**Zero-dose**	Child aged 6 to 23 months that has not received the first dosage of DPT1/Penta 1	Number of children who received DPTI/Penta I per district and per year for 2017-2019	Target number of children according to age eligible for DPTI/Penta I (as per EPI program target) per district and per year for 2017-2019	
**Vaccine-wise Timely immunization**	Child has received the dose of antigen at the recommended age specified in the EPI schedule	Number of children 12-23 months who received BCG at birth, OPV 1, 2, and 3, Penta 1, 2, and 3, Pneumococcal 1, 2, and 3, and Measles 1 as per the timeliness criteria of Sindh province	Target number of children 12-23 months (as per EPI program targets) who received BCG at birth, OPV 1, 2, and 3, Penta 1, 2, and 3, Pneumococcal 1, 2, and 3, and Measles 1 as per the timeliness criteria of Sindh province	

6. Results by study aim and objectives

The following is a presentation of results by study aim and objective.

STUDY AIM 1 (REACH). Determine facilitators and barriers to program initiation

Objective 1.1 (Program reach) Determine the proportion of eligible vaccinators trained by the program and registered within ZM-EIR from 2017 to 2019

To assess the facilitators and barriers to program initiation, the study focused on the extent to which eligible staff were trained in the use of ZM-EIR. The implementation of vaccination staff training started in the Quarter 3 of 2017. A district-wise plan of trainings was consultatively developed with the Sindh Provincial Department of Health. Nominations for the vaccination staff from districts were acquired with support from the provincial EPI program. Data on the proportion of vaccination staff out of the total were not available. However, of the 1,307 staff members nominated for training, all (100%) were trained across 52 batches between September and December of 2017. Training batch sizes ranged from 12-40 participants per training. In districts where the nominations were more than 40, the proposed participants were apportioned across multiple batches.

Overview of ZM -EIRTraining Inputs

The initial ZM-EIR training spanned for 3 days and included an overview of the ZM-EIR application including enrolment, data entry and follow up procedures as well as hands-on practice of using the Registry.

In addition to initial training, refresher training sessions were held once the ZM-EIR app was rolled out across districts. Refreshers included on-spot training conducted by District Field Coordinators at health facilities to address issues identified during their monitoring visits. Refresher training also included town/district level training for vaccinators conducted by the Program team to address both vaccinator challenges as well as orient them on any new features incorporated in ZM-EIR overtime.

Objective 1.2 (Provider use) Determine what proportion of vaccinators trained to use ZM-EIR upload details for at least 1 client in Sindh

Sindh Province EPI defines effective use of ZM-EIR by vaccinators as one upload or update for at least 1 client per vaccination day. Provider use based on the compliance criteria, drew on ZM-EIR registry system generated data and was assessed as a proportion of vaccinators who completed and uploaded at-least one child record per vaccination day activity from ZM-EIR introduction in late 2017 through 2019. Findings suggest that across 28 districts, the average compliance increased from 40% in 2018 to 57% in 2019. The top five performing districts were Naushero Feroz (85% compliance), Shaheed Benazirabad (77%), Karachi central (75%), Hyderabad (71%), and Badin (69%). While the poorest performers were Matiari (34%), Tharparkar (35%), Shikarpur (39%), Jamshoro (44%), Jacobabad (46%), and Ghotki (46%). These compliance numbers include instances in which a vaccinator may be present at the health facility, but no child is brought in for vaccination. In the future it would be helpful to include a mechanism within ZM-EIR for a vaccinator to indicate that no child has come in for vaccination to address this.

6.1.1 VACCINATOR USE BY AREA, SEX, AGE, AND EDUCATION

Stratified analysis of usage with reference to urban vs rural areas as defined by the Government of Sindh and designated within the ZM-EIR system along with sex, age-group, and educational qualification of vaccination staff was conducted to understand variances in the compliance across the above characteristics. These derivations were conducted for the district and provincial levels.

It was derived that usage across the urban vs rural areas during 2017-2019 was slightly higher in the urban segment vaccination staff compared to their rural counterparts. Compliance of usage was documented to be higher among female vaccinators as compared to males in all the considered years of 2017-2019 (Figure 3). The average proportion of female to male vaccinators was 15% female to 85% male vaccinators over the three-year study period. A similar stratified analysis for the compliance of ZM-EIR usage was conducted for the age-group

TABLE 4. PROPORTION OF VACCINATORS BY DISTRICT AND YEAR WHO UPLOAD AT LEAST ONE CHILD RECORD PER VACCINATION DAY [OCTOBER 2017-DECEMBER 2019]

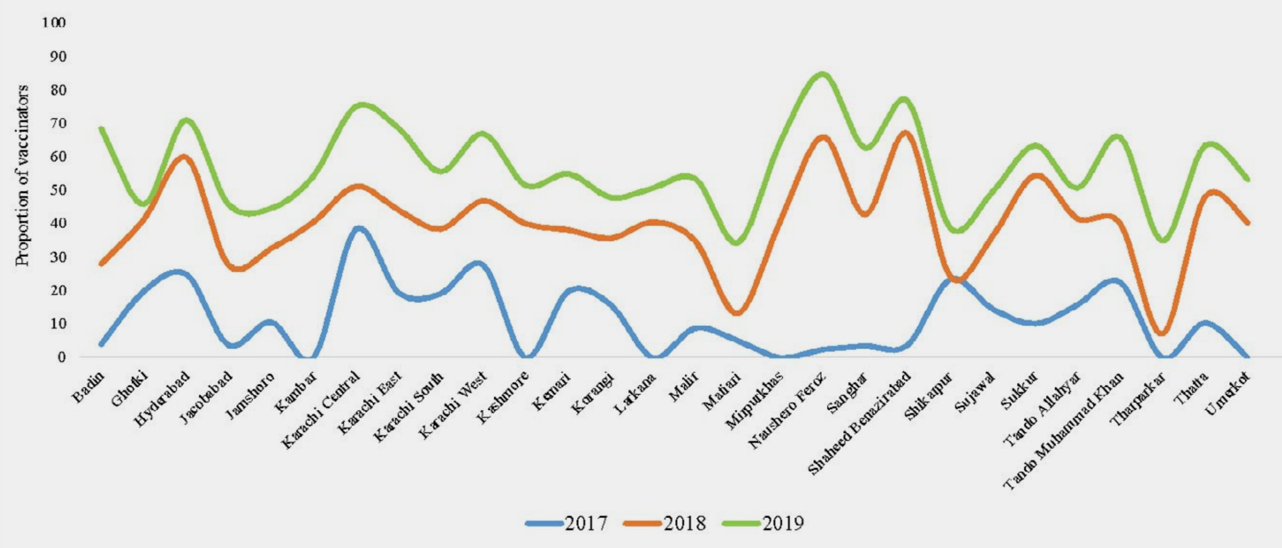

FIGURE 3. PROVINCIAL LEVEL COMPLIANCE OF ZM USAGE OF THE VACCINATION STAFF STRATIFIED BY URBAN -RURAL AND SEX (2017-2019)

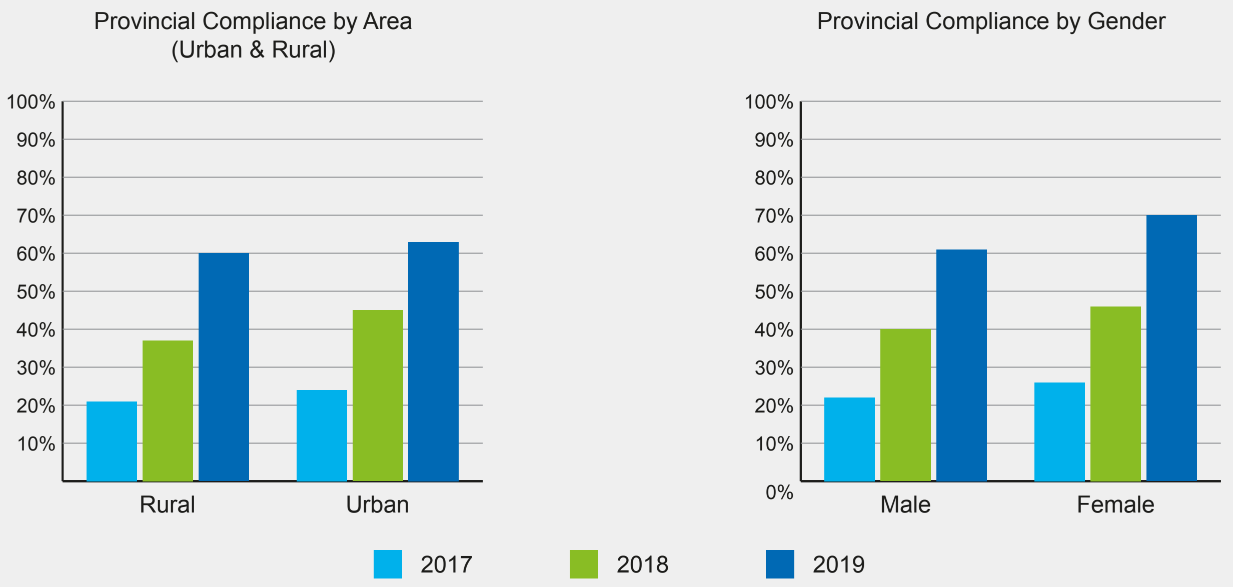

and qualifications of the vaccination staff.

It was observed that there was a gradual increase of compliance in all age-groups during 2017 to 2019. This improvement ranged between an increase from 2017-2019 of 40%-49% in the younger age groups (those less than 40 years of age), with highest proportional increase observed for those aged 26-30 years (49%), and those aged 18-25 years (47%). Lesser proportional increase in compliance was observed for the higher age-groups, with the least improvement across these years documented for the age group of 51-55 years (28% increase) and those above 55 years (Figure 3).

Comparison of compliance among the vaccination staff by their educational status/qualification reflected that increase of compliance during 2017-2019 was observed for all levels of qualifications among these staff members. The highest proportional increase was among those with Masters level and above (47% increase) and for those with Matric and Intermediate qualifications (42% increase respectively). Lesser improvement in this regard was documented for those having lower-level qualifications of Primary (30% increase) and middle level (27% increase). The findings indicate that better educational qualification among staff reflected higher improvement in the compliance rate of ZM-EIR usage, while lower qualification carrying staff had lesser improvement (Figure 3). In summary, it was observed that compliance of usage among vaccination staff reflected higher compliance among females and urban areas with better usage patterns observed for younger age groups and those with higher educational qualifications. District-wise analyses are available in the Annex.

FIGURE 4. PROVINCIAL COMPLIANCE OF VACCINATION STAFF ACCORDING TO AGE GROUP FOR 2017-2019

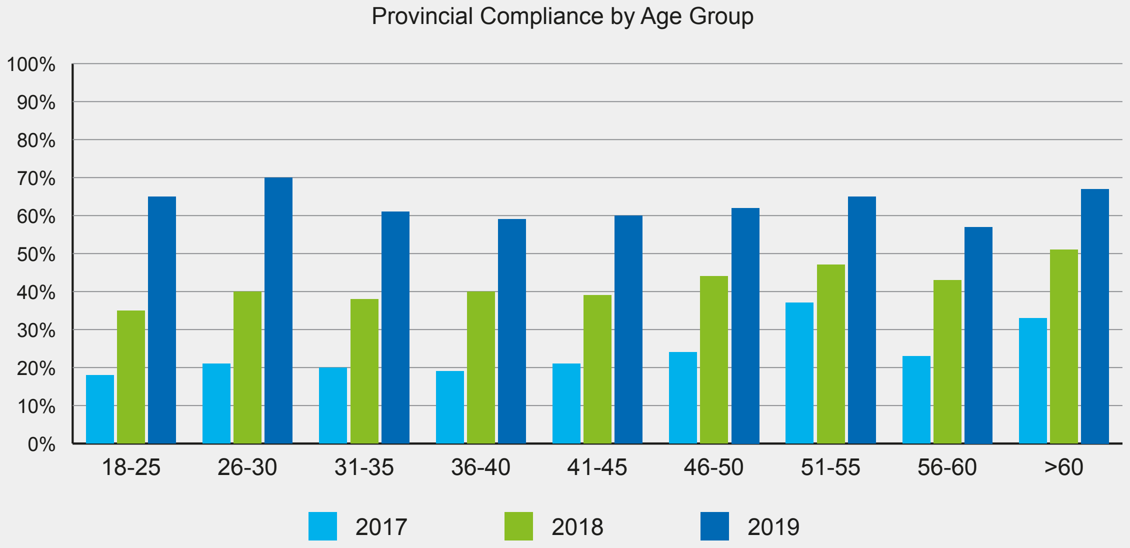

FIGURE 5. PROVINCIAL COMPLIANCE VACCINATION STAFF ACCORDING TO EDUCATION STATUS FOR 2017-2019

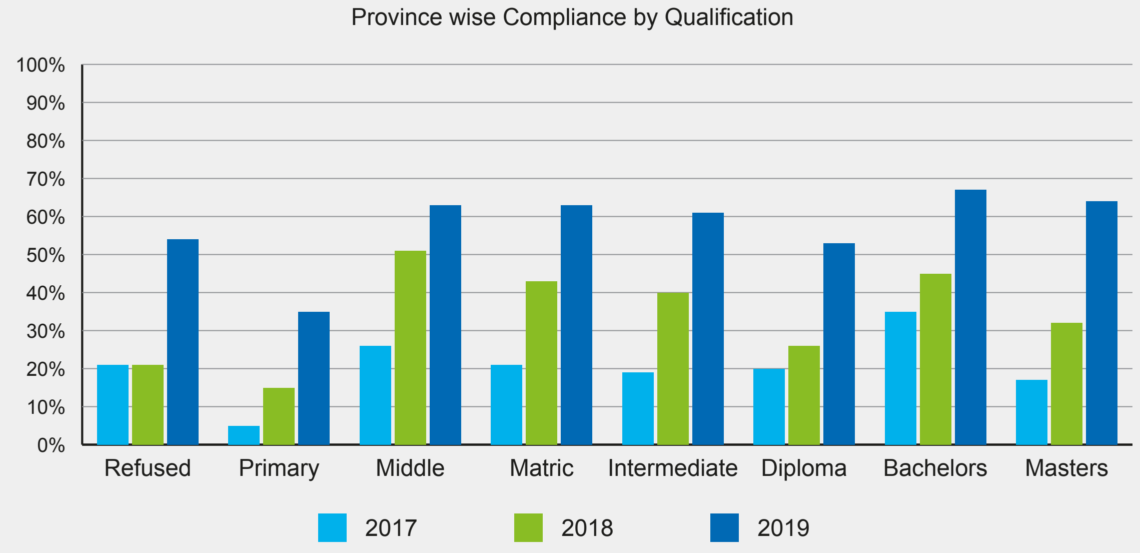

The Government of Sindh set the compliances targets in January 2019. Since that time, there has been a rise in compliance maintaining a level of 80% or higher with decreases in compliance due to time shocks like vaccinator strike, Covid, flood etc. While out of scope for the timeframe of the analysis for this study based on the protocol, the following illustration has been included at the request of IRD.

Additional Information: Monthly ZM-EIR Compliance Targets January 2019-August 2022.

Objective 1.3 (Stakeholder perceptions) Understand stakeholder perceptions about the benefits of ZM-EIR and effects on immunization service delivery (decision-support algorithm, registry, SMS alerts and reminders)

To understand how key stakeholders perceived the facilitators and barriers of ZM-EIR on immunization service delivery, in-depth interviews were conducted with five stakeholder groups, namely caregivers of children 12-23 months (n=12), vaccinators (n=12), supervisors (n=3), district field coordinators (n=3), district and provincial managers (n=6), and ZM-EIR program

MONTHLY VACCINATOR COMPLIANCE TRENDS AS PER EPI SINDH TARGETS

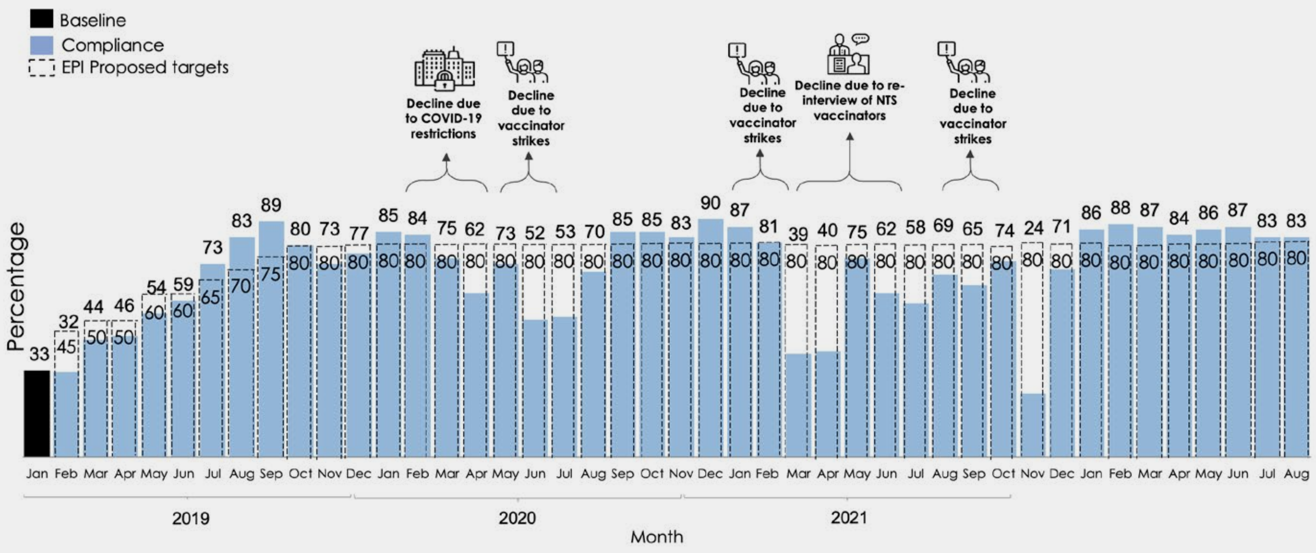

*Vaccinator compliance is calculated by the division of total number of days a vaccinator has used ZM with the total number of working days in the period

support staff (n=4) across the three districts of Jacobabad (poor performing), Shikarpur (moderate performing), and Naushahro Feroze (high performing). For each set of functions within the ZM suite of digital health interventions, key themes emerged in the qualitative data analyses, which relate to the perceived benefits and challenges in using ZM to improve immunization service delivery.

District selection took into consider high/mid/low immunization coverage and registry coverage with the latter serving as a barometer of vaccinator ZM-EIR app use. Findings suggest vaccinator profile, digital health solution design, implementation model of ZM-EIR, and aspects of the health system environment are major facets of ZM-EIR system that play a part in determining the stakeholders' interaction with it.

6.1.1.1 CONTEXTUAL CONSIDERATIONS OF THE ZM INTERVENTION

The structural aspects of the health system that relate to the use of ZM-EIR app signify the resources that facilitate its use. A total of 9 immunization facilities were observed for the purposes of the research study, out of which 6 are health facilities and 3 are district offices (spread across the districts of Jacobabad, Shikarpur and Naushahro Feroze). The number of rooms in a health facility ranged from two to seven. All the health facilities provide vaccination and other medical services. Most of the health facilities had two vaccinators present at the facility out of which one in each facility used the ZM-EIR app for registration and defaulter lists. It was observed that the internet connection was not strong enough at the facilities. The issue of server/network issues impacted the record-keeping both in the field and in the offices. Only four out of six facilities had electricity. The absence or poor condition of utilities at the health facilities is a significant factor that hindered the use of ZM-EIR and its impact on immunization service delivery. Overall, the geographic differences are not palpably different across the three districts.

6.1.1.2 PROFILE OF ZM-EIR USERS

The vaccinators and supervisors who are termed as providers in the stakeholder system of ZM-EIR in the qualitative research were found to have certain differences with regards to their age and education. While the age of the supervisors who were interviewed ranged between early 50s to late 50s, the average age range of the vaccinators was early 30s. In terms of education, the supervisors were all graduates. The vaccinators' education experience was observed to be ranging between intermediate/matriculation to college education with one holding a Masters degree. It was revealed during the interview with a district manager that because of their limited digital literacy, the supervisors did not feel fully confident in using ZM-EIR. It is likely that this is related to their age as well wherein they feel more productive and at ease using physical registries than ZM-EIR. This aligns with trends analysis of ZM-EIR compliance among supervisors who are generally older users.

6.1.1.3 GENERAL PERCEPTIONS OF ZM INTERVENTION

Overall, all stakeholder groups interviewed had a favorable impression of ZM-EIR and the associated suite of interventions. Provincial managers felt that the real time access to vaccination data facilitated longitudinal monitoring of vaccination coverage over time and across geographic areas. They further noted that expansion of ZM-EIR would allow this to occur across the country as it would allow the provinces to see and evaluate each other's coverage. ZM-EIR was thought to provide a blueprint of what a national repository of EPI records could look like and further be a mechanism for improving supply monitoring and fraud prevention. As the primary end-users of ZM-EIR, vaccinators expressed their satisfaction with ZM-EIR features, specifically for defaulter and zero dose lists. These records ensure data safety that is accessible in real-time: 'The child's vaccination record would be safe if we wrote (in the application). If the record gets lost, there is a QR code for the child that we can get through the app' (Vaccinator, Jacobabad, Male, Age N/A).

6.1.1.4 VACCINATOR PROFILE

Findings from the in-depth interviews reveal that the digital skills of the vaccinators affected their ZM-EIR app use. It was noted that in comparison to other stakeholders that interacted with the app including supervisors and managers, vaccinators were relatively more digitally literate. The average age of the vaccinators is early 30s with some educated through matriculation level. The confidence in using ZM-EIR and recording data was enhanced by the training that the vaccinators received and self-learning for some. However, in some instances, they still showed hesitance in using the app.

While perceived insufficiency of training and overall limited digital literacy for using the app might be considered valid reasons for why the vaccinators display hesitance, finding the right champions for this paramount role of immunization service delivery precedes any technological or skill-level obstacle. In order to successfully integrate the usage of the app, the ZM-EIR support staff explains, it is crucial to be '. . . engaging with the end-user, spending sufficient time training them so that the initial learning curve is not that steep and then the lesson that we have from Sindh is finding the right champions from the government who are able to realize and value the importance of the information coming in through the registry and are able to create that positive culture of usage among the end-users' (ZM Staff, Female, Age N/A).

6.1.1.5 PERCEIVED UTILITY OF ZM

Regardless of their digital abilities, the vaccinators showed understanding of the usefulness of ZM-EIR and its data. It was observed by the managers that the vaccinators used the ZM-EIR app and government-provided mobile phones very carefully. An interesting response about the use of the ZM-EIR app was that 'these boys (vaccinators) are scared to use them (ZM-EIR app) because they think if they tamper with them a little, they will lose all their data. That's why they don't touch that mobile. When they leave the field, they fold it up in a handkerchief and leave it' (District Manager, Naushahro Feroze, Male, Age N/A).

ZM-EIR allows the vaccinators to have a daily plan in terms of immunization service delivery and helps them direct their effort to where it is required. Given the lists of zero-dose children and defaulters, the vaccinators keep track of those who have never been vaccinated, those who require a second dose, and those who have had their doses in different places. It is perceived that the ZM-EIR app has enabled vaccinators to carry out their immunization work easily and honestly, increasing work accountability.

The app provides real-time data on '…how many vaccines have [been] used and how many syringes have we used' (Vaccinator, Shikarpur, Male, Age 38) for vaccine stock management. Coordinators and supervisors access vaccinator attendance on the app wherein they can track the vaccinator effectively. A district field coordinator said 'wherever he goes, we monitor it through google map. When we visit a vaccinator, it is a surprise visit. We do not call them. We just follow them on mobile and visit them on field' (Coordinator, Jacobabad, Male, Age 38). The managers emphasized how the attendance data is available with 'just one click'. Additionally, managers conveyed that monitoring of immunization activities is carried out daily. They share a compliance graph on communication groups with other stakeholders. These graphs depict 'which vaccinators

have complied [based on required vaccinations for the day], and which are still non-compliant' (Coordinator, Naushahro Feroze, Male, Age 29). For the coordinators and supervisors, it is perceived to be more cost-effective than what they had been doing prior to the ZM-EIR app since they do not need to physically go to the field to know what is happening.

6.1.1.6 IMPLEMENTATION MODEL

Key informants shared their perceptions of a range of aspects related to the implementation model of ZM-EIR, including those related to provision of mobile phones, trainings provided, double data entry (ZM-EIR app and paper-based records), and health system environment.

Among the informants, there were two vaccinators and two supervisors who regarded the lack of adequate phone distribution for ZM-EIR use as an issue. A supervisor explained that the vaccinators have not received the mobile phones and that '[the vaccinators] do the entry using mobile from someone else (Supervisor, Jacobabad, Male, 56). Among the vaccinators who were interviewed, two of them reported lack of mobile data package (internet connectivity) as an obstacle and expense when using the ZM-EIR app. One of them suggested increasing the amount of mobile data provided since 'out of 30 days, we have to use our personal data for 20 days (Vaccinator, Shikarpur, Male, 28). Four respondents shared that they often have had to use personal money for ZM-EIR use. One respondent shared that '[he] spends almost 1000-1200 on this personally because network is not there (Vaccinator, Naushahro Feroze, Male, 30).

IRD Phone Distribution Process

Phones are distributed to all vaccinators through the District Health Office (DHO) in line with the official vaccinator list available with the DHO. Vaccinators are informed that the phones are the property of the Health Department, Government of Sindh. If the phone is lost or broken, it is the vaccinator's responsibility to replace it. A written application needs to be submitted to the DHO in case of loss or damage to the phone.

For the internet packages, when the program started in 2017, a package of 2GB per month was provided to vaccinators based on their maximum requirements to upload data. This was increased to 2-5GB in June 2021 depending on the mobile network packages available in each region. In case the internet package is exhausted within the month, the Provincial and District Health Office require that vaccinators use their own resources for internet connectivity to make sure that all required data is captured within ZM-EIR.

The vaccinators confirmed that they had received training from the staff at Interactive Research and Development (IRD). This training has enabled them to make the most out of this app especially in utilizing the app to communicate with parents; *'the addresses and the mobile numbers help us. If someone loses their card, we search their name, and through that we can find out if the child needs to get an injection'* (Vaccinator, Shikarpur, Male, Age N/A).

In terms of record-keeping, there are mixed opinions regarding how impactful ZM-EIR has been in terms of workload for the vaccinators. While some believe that by sparing them from making entries on paper registers, ZM-EIR is reducing their workload to a great extent. However, some have had different experiences. They believe that only one method of recording entries should be followed. One vaccinator shared their experience; *'our people in the field write in the book, on the card, and all that writing is very difficult. It would be better if we just use ZM-EIR, and only one thing stays'* (Vaccinator, Shikarpur, Male, Age N/A). This has led to resistance from the vaccinators and unions as the expectation of dual record-keeping remains. One coordinator proposed that the district health officers and focal persons are responsible for the resistance. According to him, *if they had owned it properly, it would end. The vaccinators who work honestly in the field ask us to put an end to the manual work so they can continue working with ZM'* (Coordinator, Shikarpur, Male, 38). It is perceived that the use of ZM-EIR cannot be fully maximized unless physical record-keeping is phased out as it impacts the potential impact the ZM-EIR app can have on immunization service delivery.

Despite the administrative issues, the district field coordinator in Shikarpur seemed very confident about the use of ZM-EIR. He believes that ZM-EIR use is promising for the district. He said, *'all their registers will cease to be used, and all their data will be done on ZM. That will be their daily register and stock register. Work is being done on this, and I think in one or two months they may become paper-free'*.

6.1.1.7 PERCEIVED BARRIERS TO SUCCESSFUL IMPLEMENTATION OF ZM-EIR

Vaccinators across the three districts of Jacobabad, Shikarpur, and Naushahro Feroze informed the interviewers that they use the app daily and responsibly and have had received training for its use. The two main perceived barriers that are faced by vaccinators in terms of using ZM-EIR app across the three districts include workload due to dual burden of paper-based reporting and ZM-EIR data entry alongside resistance from caregivers.

It was reported that when the caregivers would bring their child for vaccination, the vaccinators did not use the ZM-EIR app for registration. The success of implementing the use of ZM-EIR app was heavily affected by the volume of vaccination cases and general negative public perception that the phone is being used for non-clinical activities, e.g., playing games. In the interactions between the caregiver and vaccinators, public perceptions especially of those caregivers who remain unaware of the ZM-EIR app are often skewed. A vaccinator shared their experience; *'what can we do when we have a lot of children (to attend to)? We can't use (the app immediately). Here, no one has the time. It takes time to copy the information to ZM. Whoever comes here (during the rush hours), they will not realize that we are using ZM. They would say "oh look, we are waiting, and he is using the mobile, he's playing games'* (Vaccinator, Jacobabad, Male, Age N/A). The negative public perceptions may waver a vaccinator's confidence in using the ZM-EIR app and utilizing its data leading him to completely rely on traditional record-keeping.

In Jacobabad, vaccinators shared that caregivers often complain when they take pictures for record-keeping. As one of the vaccinators shared, *'we take photos as well, as they are important for our daily coverage, and for sending in our WhatsApp groups. So, they complain that why are you taking our pictures'*. This can be related to public ignorance in the words of a supervisor from Jacobabad; *'Our area is backward. They do not know what we are doing and what we are not doing'*.

In Shikarpur, vaccinators faced great difficulty in soliciting contact information from the parents because in their experience, women and older children are the ones who often bring younger children for vaccinations. Women and older children neither own phones nor have contact information to share with the vaccinators making the app use quite limited.

6.1.1.8 CAREGIVER EXPERIENCES WITH ZM-EIR: AWARENESS AND EXPERIENCES

Based on the interviews conducted with the caregivers, two out of 12 caregivers were familiar with ZM-EIR as an application that is used to record immunization data at the health facilities, however, caregivers demonstrate better awareness about the other components of ZM-EIR. Caregivers understand the importance of recording the data which is fulfilled through scanning the QR code to check if [the child] is vaccinated or not. The caregivers believe that apart from playing a role in recording information, the reminders for vaccinations is very important. When asked how they feel about the reminders, they expressed their satisfaction, *'...it is very helpful. Otherwise, we are busy with our work and forget about it. When we receive a message then we know we have to get the child vaccinated soon'* (Caregiver, Shikarpur, Female, Age N/A).

The call center helpline service has been developed to open a direct communication channel for the caregivers to address their concerns. The helpline answers different concerns and questions such as the location of the nearest immunization center. The call center feature serves as a blueprint for redesigning communication strategies in accordance with the data. *'For us as researchers', a ZM-EIR support staff member said, 'we developed our chatbot feature keeping this in mind. Since the chatbot is AI-powered, it is a very automated thing and because the queries were so simple, we could have automatic responses to them since it is all linked to ZM so it would know that this child is living in this locality'*.

The call center enables the caregivers to communicate with the vaccinators. Two out of 12 caregivers reported using the call center service once to inquire about vaccinations and another time to express concerns and ask about fever caused after the vaccinations. Coordinators suggest that the helpline has proven to be useful in lodging any complaints about the vaccination process or vaccine itself. A coordinator from Jacobabad describes the use of the helpline; *'it's for complaints . . . if a parent wants to complain that a vaccinator didn't visit them, for instance. We have gotten many complaints like this in Jacobabad or about vaccine side effects. So, some educated people here do complain through the ZM call center'*. The comment from the coordinator suggests a likelihood for educated people in the districts availing the service more than uneducated ones. The caregivers who used the call center, called it, *'good in every way'*. According to them, these have been proved useful in gaining information about the availability of the vaccination teams, discussing any symptoms caused by the vaccination, and asking for a second dose.

6.1.1.9 WOMEN'S ACCESS AND USE OF MOBILE PHONES

Given the cultural norms of limited physical mobility for women and male-female segregation that increasingly persist in rural contexts of the districts under study, women's involvement in direct communication for their children's vaccination purposes is limited. Because of the norms of shame and honor, women resist interacting with male vaccinators and refuse to share their mobile phone numbers for SMS reminders. To mitigate this challenge, one of the vaccinators suggested that recruiting female vaccinators is likely to help gain the trust of female caregivers and ease the immunization process.

6.1.1.10 RECOMMENDATIONS FROM CAREGIVERS

The text message reminders were perceived as beneficial for most of the caregivers interviewed. For some, the reminder mechanism requires minor changes. This is because in the local contexts of the district, the literacy levels of the caregivers vary. While some caregivers are comfortable in reading text messages in Urdu (national language), others accentuated the need for text messages in Sindhi (local language). An alternative of *'a call to alert [them]'* was also suggested. Moreover, the effectiveness of a SMS reminder and its intended use to encourage the caregivers to bring their child/children for vaccination is also often dependent on what the caregivers themselves prioritize more. One of the managers from Naushahro Feroze explains this phenomenon; *'when the uneducated ones get a message, they don't even know who it's from. There aren't any educated people here, and we don't have a lot of outreach among the ones in the city . . . when it's from Benazir Support Program, they go and make the whole village read it saying, "the money's here"'*. The supervisors attributed that the lack of literacy and motivation inhibits some caregivers from fully utilizing the potential of ZM SMS reminders for their child's vaccinations. When caregivers/parents do show up for scheduled vaccination appointments, they often do not know about the app or its use. Those who do know appreciate the app since they can receive SMS reminders for vaccinations that are due.

Adding a verbal communication component within the reminder mechanism might expand the immunization coverage. As mentioned above, caregivers often find it difficult to fully rely on SMS as a reminder for them to get their children vaccinated. More often than not, an SMS reminder doesn't achieve what it is intended to because parents are not literate enough to read the SMS reminder. In those situations, the managers think it might be more useful to call the parents to remind them.

STUDY AIM 2 (REGISTRY COVERAGE). Determine proportion of children amongst those eligible at a population level contained within the ZM-EIR registry

Objective 2.1 Determine the number of unique children contained within ZM-EIR vaccination registry

Registry based coverage was calculated using the enrolment numbers for each calendar year birth cohort as numerator and the annual targets provided by the EPI (ascertained population based on modelling using birth rates and recent census figures). The target population of children is derived based on the enrolment of children at the time of birth through a birth cohort registration and an EPI schedule-based continuum of care mechanism.

From 2017 to 2019, the ZM-EIR immunization registry enrolled EPI records of nearly 5.4 million children and 1.9 million married women of reproductive age group (MWRA Aged 15-49 years). One possible explanation provided by IRD for the negative change in coverage between 2018-2019 in certain districts is selection-bias i.e. the increased number of children getting vaccinated in 2018 reflect a backlog of children from previous years who would otherwise not have been vaccinated but are getting vaccinated at a later age with the implementation of ZM-EIR.

TABLE 5 PROPORTION OF CHILDREN 12-23 MONTHS CONTAINED WITHIN ZM-EIR FROM 2018-2019

Name		ZM launch date	2018			2019			2018-2019
			Target	Actual	Registry coverage	Target	Actual	Registry coverage	Change in coverage
1	Karachi South	Sep 6, 2017	-	36 862	-	69 181	45 890	66%	-
2	Karachi Central	Sep 23, 2017	-	83 794	-	124 840	87 613	70%	-
3	Karachi East	Oct 6, 2017	-	52 609	-	89 148	72 527	81%	-
4	Badin	Oct 10, 2017	54 403	31 375	58%	65 188	68 041	104%	47%
5	Matiari	Oct 31, 2017	23 010	6 618	29%	23 779	14 470	61%	32%
6	Karachi West	Nov 21, 2017	-	62 418	-	112 103	112 122	100%	-
7	Hyderabad	Nov 23, 2017	69 596	71 288	102%	72 741	63 442	87%	-15%
8	Tando Allahyar	Nov 28, 2017	22 977	25 272	110%	30 118	24 999	83%	-27%
9	Tando Mohd Khan	Nov 29, 2017	19 035	20 757	109%	24 780	33 697	136%	27%
10	Jamshoro	Nov 29, 2017	29 178	24 796	85%	34 385	12 449	36%	-49%
11	Malir	Dec 4, 2017	-	33 404	-	49 101	51 555	105%	-
12	Sujawal	Dec 9, 2017	24 746	17 268	70%	28 502	15 072	53%	-17%
13	Sukkur	Dec 11, 2017	46 773	55 497	119%	52 145	51 934	100%	-19%
14	Thatta	Dec 12, 2017	28 563	28 436	100%	36 114	33 009	91%	-8%
15	Ghotki	Dec 16, 2017	52 235	35 104	67%	60 802	43 641	72%	5%
16	Shaheed Benazirabad	Jan 4, 2018	47 646	88 785	186%	58 109	56 523	97%	-89%
17	Sanghar	Jan 4, 2018	73 605	59 322	81%	75 440	69 443	92%	11%
18	Naushehro Feroze	Jan 5, 2018	48 336	80 130	166%	58 821	63 411	108%	-58%
19	Mirpurkhas	Jan 5, 2018	45 571	34 467	76%	53 684	54 887	102%	27%
20	Umerkot	Jan 27, 2018	35 771	31 095	87%	39 370	37 823	96%	9%
21	Jacobabad	Jan 29, 2018	34 622	18 587	54%	31 453	24 416	78%	24%
22	Kashmore	Jan 29, 2018	31 904	40 656	127%	38 223	38 962	102%	-25%
23	Larkana	Jan 30, 2018	53 221	40 140	75%	47 074	50 119	106%	31%
24	Tharparkar	Feb 10, 2018	48 469	5 919	12%	32 934	25 492	77%	65%
25	Kambar	Feb 19, 2018	49 056	38 198	78%	37 519	39 011	104%	26%
26	Korangi*	Jan 1, 2007	-	60 923	-	62 191	59 821	96%	
27	Shikarpur*	Oct 15, 2015	42 460	38 682	91%	37 144	46 736	126%	35%
Total			881 175	1 122 402	127%	1 444 892	1 297 105	90%	

*These districts were part of the pilot implementation and ZM-EIR was launched in these districts prior to provincial scale-up.

Objective 2.2 Conduct in-depth interviews and observations with vaccinators, clients, and supervisors (and other key stakeholders) to understand their views on differences in registry coverage observed and effects on uptake of immunization services

6.1.1.11 PERCEPTIONS RELATED TO THE IMPACT OF ZM-EIR ON IMMUNIZATION COVERAGE

Findings suggest that ZM-EIR features like defaulter and zero dose lists and SMS reminders have led to improvements in coverage of immunization services. The defaulter and zero dose lists help uncover areas and populations within regions that are either not completely immunized or are only partially immunized. According to the managers, the defaulter and zero dose lists on ZM-EIR app identify which specific regions require a stronger effort in terms of coverage. A provincial manager shared that these lists show *'…in what [performance] category are [our] UCs, i.e., whether [they] are in red, green, blue, or yellow . . . every UC-wise, every vaccinator-wise, every town-wise, every district-wise, we are getting all the data'*.

The timeliness and overall efficiency of immunization coverage is also perceived to be improved through SMS reminders targeted at caregivers. A female ZM-EIR support staff mentioned the specifics; *' . . . three SMS reminders are sent so it's not that you only get one SMS reminder for the child's appointment day . . . there are these three different time points as a constant nudge to the caregiver to bring the child for immunization at the time that he is required to show up'*.

Attendance reports through ZM-EIR provide data on immunization assignments for the vaccinators which helps in ensuring accountability. One district field coordinator from Jacobabad exemplified the accountability in immunization coverage through ZM in the following way, *'Because of ZM, the EPI office has found out that we have 114 vaccinators in Jacobabad. If only 67 vaccinators out of them are working, they ask your district where the rest of your vaccinators are. They ask us to enroll them, assign them a field, and send them to work. So now I have over a hundred vaccinators enrolled, and they work themselves after returning. Coverage will thus ultimately increase'*.

The increase in immunization coverage is also related to more structural factors e.g. public trust in government. From a ground-level perspective, vaccinators who are the closest to the clients i.e., children and their caregivers shared that the selection of local people for authority positions in rural governments has increased the trust of people in government, leading local caregivers to trust health authorities and their immunization efforts. In general, the vaccinators also believe that there has been an increase in awareness coupled with an increase in diseases which has had a concurrent effect on improved vaccination rates among rural communities.

Gender plays a significant role in immunization coverage owing to its embeddedness within the local, rural contexts. There is often a clear distinction between caregiver's interaction with male vaccinators and that with female vaccinators. One key informant emphasized the need for lady health workers who can bond with female caregivers (who are usually more responsible for taking care of their children than male caregivers) to resolve any resistance or anxieties regarding vaccinations in an attempt to further expand immunization efforts. A female caregiver from Naushahro Feroze, in fact, shared that *'the female health worker can come inside the house, and we [caregiver] know her so it's good that a female health worker comes. If a man comes then it is quite difficult'*. These deeply embedded gendered preferences related to vaccination experiences reveal that it might be essential to incorporate cultural elements when designing from an end-user lens.

6.1.1.12 PERCEPTIONS RELATED TO THE IMPACT OF ZM-EIR ON IMMUNIZATION EQUITY

Equity in immunization refers to availability, provision, and timeliness of vaccination services for all children (rich/poor, male/female, and urban/rural). From the lens of the caregivers, the number of vaccinations received has increased among the poorer, less educated, and rural social stratum. However, the reasons are not directly attributed to the role that the ZM-EIR app is playing. According to them, in recent years, more vaccination centers have been established and a change in public attitude towards vaccinations where they believe that it is a public health necessity that proves beneficial for the health of residents. This increase in awareness has also in turn led to receptivity to home visits by healthcare workers who were often not openly welcomed by the rural communities in the past.

The question of whether the vaccination has increased in specific socio-economic areas of the province is associated with how the data is reported and increased participation of private health facilities over time. According to the ZM-EIR staff, in the initial days of ZM, there was greater focus on strengthening electronic immunization registry in public health facilities over private health facilities; *'in 2015 when ZM was scaled up, the bulk of it was focused on public health facilities. Those were the priority, scaling up the public health facilities. And at that time, there was a very small proportion of private health facilities . . . since we are headed by the Government of Sindh department, the focus was on public health facilities, to concentrate on them and make the EIR stable across those and then you gradually add more private clinics and GPs . . .the bulk of the registry is lower- and lower-middle-income level because they are utilizing public facilities more so generally they would form a disproportionate part of the data when analyzing'*. It is important to consider that the changing trends of immunization activity after the emergence and spread of the use of ZM-EIR may have been initially more related to an increase or decrease in the record-keeping in some instances and less to an actual increase or decrease in coverage. Since ZM-EIR app operates under the mandate of leaving no one behind in the immunization processes, the use of ZM-EIR now increasingly pervades both private and public health facilities. Since ZM-EIR is an intervention mandated and supported by the government, private facilities that use vaccines supplied by the government are obligated to use the app. As ZM-EIR support staff corroborates, *'we have made a common rule with the health minister, those who collect vaccines from District EPI store has to submit a consumption report through ZM-EIR . . . penetration/access (of resources) has increased and so would the utilization. Though earlier there was access but there was no data sharing mechanism from the higher socio-economic status people so that's why'*.

In terms of gender equity, there are often strong, temporal patterns underlying the disparities between female and male child vaccinations. These include the dynamics of cultural sensitivity, better education, and overall awareness with regard to diseases. For example, if education facilities have been developed and improved, that seems to have naturally fed into a trend of more female children getting immunized. But additionally, ZM-EIR features have also led to an increase in female children getting vaccinated. The ZM-EIR staff pointed out that *'in outreach, more female children are being vaccinated because they find it easy that they can get the immunization service at their doorstep . . . we can see [in defaulters list] that more females might be lagging behind as a result of this, there is catch-up and we do see better coverage among the female population'*. This adds validity to the usefulness of the defaulter lists as a feature of ZM-EIR. Moreover, the supervisors acknowledged the role of lady health workers who pay field visits and motivate parents to get their children inoculated. The role of personal communication and trust-building can play a big role in increasing vaccination rates overall. The district

managers are of the view that access to ZM-EIR app should be expanded to the lady health workers.

To further strengthen coverage and equity in immunization efforts through ZM-EIR app, the managers support the view that all health facilities in rural areas should have EPI centers. They also suggested that the goal should be to offer training for ZM-EIR first and introduce it to them beforehand after which no child would be left out.

STUDY AIM 3 (DATA USE). Determine the availability and use of ZM-EIR data across key stakeholder groups

Objective 3.1 Conduct secondary analyses of system generated data, reports, and dashboards to determine frequency of ZM-EIR data use by supervisors, managers, and Government staff at all levels

Human Resource Log-in (HR Log in) is the database feature, that facilitates the ZM-EIR to document and analyze the usage of ZM-EIR Dashboard by supervisory and management staff members of the EPI program, that include district level and provincial level users. Similar to the approach for compliance data, HR log-in based summary statistics are generated and analyzed on a regular basis, given the flexibility of ascertaining management and supervisory staff usage at the granular level of the vaccination center/health facility. In charges/managers, district EPI and other members of the district and provincial health department, as well as the features of time-wise summaries generation for daily-weekly-monthly-yearly basis.

In 2017, there were 18 managerial staff, and 5 supervisory staff. This increased to 354 managers and 199 supervisors respectively in 2018 and 452 managers and 373 supervisors in 2019. The evaluation conducted overall usage which showed that there was a gradual and consistent increase in the number of logins by supervisory staff during 2017-2019, while a slight dip was seen in the usage by managerial staff cadres from 2018-2019, after having a marked increase between 2017-18. Keeping view of the increase in overall volume of usage among both managerial and supervisory staff categories, when compared across the overall denominator of all user logins, a gradually increasing proportional representation during 2017-2019 was observed for supervisory staff as compared to the management staff. A review of these findings, reflect that an increase in usage by both managerial and supervisory staff was observed for the number of logins, however, as the volume of overall usage increased between 2017-2019, the proportional representation of supervisory staff reflected an increasing trend as compared to managerial cadres. In this regard, district-wise generated graphs of usage are added as annexures.

Objective 3.2 Understand stakeholder perceptions on the differences in newly available data (disaggregated gender data, geographical data, GIS analysis, vaccinator monitoring data), data use patterns, perceptions on link between data use and immunization coverage, completion, and timeliness

To understand differences in data and data use patterns, in-depth interviews were carried out with key stakeholders, including vaccinators, supervisors, district, and provincial managers and ZM-EIR staff.

6.1.1.13 DATA USE FOR IMMUNIZATION SERVICE DELIVERY

Vaccinators use the app daily beginning with marking their attendance as one of the first tasks. The data reports on ZM-EIR are shared with District Health Officers on a day-to-day and monthly basis. These reports help them undertake UC categorization. This data is then presented in meetings hosted by Commissioners. Because the data reporting involves multiple stakeholders, communication is paramount. The District Field Coordinators meet the vaccinators based on a field plan every month. If there are any issues related to ZM-EIR, the coordinators contact them themselves the same day. If there is an issue, they would visit them and resolve the issue. The coordinators are also in frequent contact with district managers and supervisors. They speak to them 'daily' to verify vaccinators' attendance. One coordinator said, *'when we have their attendance in the morning, the supervisors call right then to ask why this vaccinator isn't marked present, tell their timing and all. So, we talk to them daily'*.

FIGURE 6. NUMBER AND PERCENTAGE OF HR LOGINS STRATIFIED AMONG SUPERVISORY AND MANAGEMENT STAFF CADRES OF EPI PROGRAMS

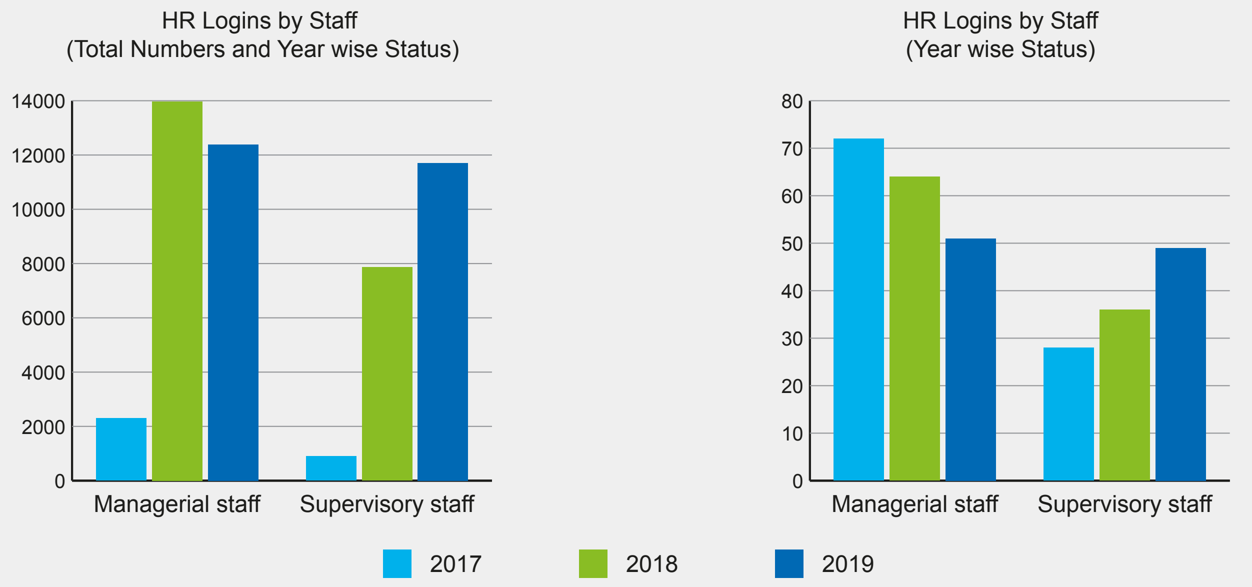

The defaulter list has been a key focus of the government in terms of EPI. These lists are automatically generated on the app. Managers and vaccinators reported using the app to investigate the presence of zero dose children. For the latter one manager noted: *'I can also see everything about defaulter children and why we can't cover them* (District Manager, Naushahro Feroze, Male, Age N/A). Managers noted feeling pressure to identify defaulters and valued the ability to send alerts, reminders and directly call to remind the caregivers about appointments.

6.1.1.14 DATA USE FOR SUPERVISION

Among the stakeholders who can access the data reports and dashboards, the role of supervisors has been seen to be relatively limited compared to others. The supervisor's overall responsibilities include monitoring and providing support to vaccinators, checking vaccination stock, and planning and conducting field visits. For their limited use of ZM-EIR app to monitor the activities of vaccinators in their respective district areas, it was stated that newer and younger supervisors are able to use the app comfortably while older supervisors have found the app difficult and less engaging. Regardless of the unease, most of the supervisors interviewed agreed that *'they should get entered as a guest so [they] can supervise… [supervisors] should have a dashboard. If they have that, they can do everything'* (Supervisor, Lakhi, Male, Age 58). Coordinators and supervisors also suggested that the latter should be given access to a separate app to monitor the vaccinators so that they do not have to rely on the district managers for the data.

With regards to the specific roles and responsibility of the supervisors, *'[supervisors] do monitoring by checking their vaccines, by checking their temperature in the monitoring. We check the graph and record to check whether they are taking records or not. We also check whether they [vaccinators] use ZM or not. This is our work'* (Supervisor, Naushahro Feroze, Male, Age 53). However, the usefulness of ZM data is limited for them due to their limited use of the app. They often rely on district focal persons or coordinators to monitor vaccinator attendance. As one of the supervisors said, *'we can't tell if it is useful for the supervisors. Because we don't know where the vaccinators are. We should be able to check their work through it, even as a guest or monitors. When I ask these things, sometimes [district focal person] is busy, and sometimes he does help. If this app was for our use as well, then it would improve'*. The district managers believe that giving the supervisors access to their own version of the ZM-EIR app can help distribute the responsibility of monitoring the vaccinators, However, effective training would be required because *'all supervisors are old-aged. If we make them use it, they won't be able to. They can't even see the dashboard, because those poor people aren't used to it since the start'* (District Manager, Jacobabad, Male, 35).

The use and impact of ZM-EIR app data is dependent on the acceptability of its value among all stakeholders. In the experiences of the supervisors, they believe that the caregivers are not fully convinced of the value of ZM-EIR. A supervisor from Jacobabad shares with the interviewer, *'[ZM] is of value to us. But the parents do not know the honor and the value of ZM'*. The resistance from a caregiver's end discourages the vaccinators to record data accurately, affecting the validity of the data.

6.1.1.15 DATA USE FOR IMMUNIZATION SYSTEM PERFORMANCE ASSESSMENT

The provincial managers shared that the ZM-EIR app provides defaulter and zero dose lists which help them meet their target and improve UC performance. It enables those who are supervising to encourage the vaccinators to bridge the gaps in the coverage of immunization targets. The data is reliable because it is not dependent on the availability of internet services. This implies that the data is recorded and updated as and when the vaccinators inoculate the children that are assigned to them. This data is then communicated to other responsible representatives at the district and provincial levels. As a district manager from Naushahro Feroze shared, *'on our last date, we take monthly reports from all the boys. We receive them by the fourth of the month. Our monthly report goes ahead from here directly'*.

The managers shared that timeliness of immunization is being improved through the decision-support system built within the app. While the SMS reminders as a ZM-EIR feature are more caregiver-oriented, the decision-support system within the app integrated as an explicit function is more vaccinator-oriented. In the instances where vaccinators haven't had substantive training, the vaccinator is likely to get confused about which vaccine to give the child at a specific time. The decision-support system calculates for them. It has a built-in algorithm that automatically calculates which vaccine is to be given based on the date of birth of the child and the immunization history. This minimizes the chance of missed immunization and enables the vaccinators to provide age-appropriate and timely immunization.

Data on ZM-EIR enables an assessment of cross-facility trends and facilitates monitoring of UC performance. The district field coordinators suggested that ZM-EIR has enabled them to undertake UC categorization which acts as a form of accountability measure and performance monitoring tool. A coordinator from Jacobabad shared that given the feature of the defaulter list which has automated real-time data, *'the vaccinators are also scared because when their defaulters increase, their UC goes into categorization B or C. So they are working on the coverage of defaulters because with ZM we are getting proper data now. Since the defaulters are being covered to keep their UCs up, that means vaccinations are happening timely'*. In the eyes of the field coordinators, the credibility of ZM-EIR as an electronic immunization record app is high because the data is valid and authentic.

6.1.1.16 RECOMMENDATIONS TO IMPROVE DATA USE

It was highlighted that an attitude of greater receptivity and openness to digital health technology is as important as hours of training for a better use of ZM-EIR data. These concerns from an end-user perspective and their embeddedness within a larger digital health system indicate the need for a few major improvements which are revealed from the responses of ZM-EIR staff members and provincial managers.

First, according to ZM-EIR staff, the application needs to be more user-friendly and accessible to ensure the fulfilment of its objectives. According to the respondents, an adequate provision of mobile phones and mobile data, better server and internet connectivity services, SMS reminders in local languages (Sindhi), availability of an offline mode for app, and activation of stock management features can help make the ZM-EIR app more user-friendly. Second, the reluctance on the part of external auditing organizations to accept use of ZM-EIR electronic data instead of paper-based immunization records despite the availability of ZM-EIR's real-time data limits the use of and potential impact of ZM-EIR.

In addition, provincial managers recommend two major changes that would ease supervision and monitoring through ZM-EIR. First, ZM-EIR coordinators should be facilitated more in terms of financial incentives and additional support. In a district with 21 UCs, it is extremely difficult for one supervisor to be overlooking a huge number of EPI activities across a wide region. Second, ZM-EIR is a repository of crucial data. A lot

can be extracted based on what is required for assessment or supervision. A statistician or research team would be a useful addition to help in organizing the data for proper decision-making with regards to EPI activities.

Other improvements to promote ZM-EIR app use and expand immunization coverage include the need for a supervisor app, an adequate provision of mobile phones and mobile data, better server and internet connectivity services, activation of stock management feature, expansion of ZM-EIR app to other provinces especially Balochistan (children from Balochistan often are vaccinated in the districts of Sindh but the lags in records make these entries appear under defaulter lists). At a more advanced level, the senior ZM-EIR staff placed an emphasis on a greater use of data. They suggested the use of ZM-EIR predictive models to predict children that might default to redirect focus on greater efforts for immunization incentives accordingly.

6.1.1.17 ZM-EIR EXPANSION TO OTHER REGIONS

In terms of the expansion of ZM-EIR, managers were convinced it would be useful for the provinces to see and evaluate the coverage in their respective regions. Additionally, they were of the view that *'when this immunization history will be added through passport ID and CNIC'*, it is important that there should be a unified repository' Provincial Manager, Male, Age N/A). ZM can provide a blueprint for what a national repository of EPI records can look like across the country, beyond the province of Sindh. This is a crucial suggestion in the view of the provincial managers because in their experience, fake records of vaccinations exist. With a robust tracking system that ZM-EIR app provides, fraud and errors can be tracked. The interaction of a manager with a resident of Khyber Pakhtunkhwa (KPK) exemplifies the need of ZM-EIR expansion with its fraud prevention benefits. The resident said, *'I think it should be all over Pakistan, and the example of KPK is right in front of you, you know that [polio] cases are appearing after 15-16 months. Now I . . . met a Pathan (person from KPK) from the area where the cases emerged, he said why are you providing drops to our children, we only do finger marking. So, they (vaccinators) don't provide drops according to him, this application (ZM) tells us everything'* (Provincial Manager, Male, Age N/A).

Objective 3.3 Conduct direct observations of immunization services to understand the broader context within which implementation is occurring and to identify barriers to data entry and use

Across the districts of Jacobabad, Shikarpur and Noshero-Feroz direct observations of immunization services were conducted for one day per facility in six health facilities and three district offices.

Among the health facilities observed, half of the facilities observed were clean, organised, and relatively well-maintained. The other half were seen as unclean, 'cluttered', and with 'broken furniture'. The number of rooms in a health facility ranged from two to seven. All the health facilities provide vaccination and other health services. Most of the health facilities had two vaccinators present at the facility. Of these one in each facility used ZM-EIR for registration and defaulter lists. In all six facilities, it was reported that when the caregivers would bring their child for vaccination, the vaccinators did not use ZM-EIR for registration in real-time. Only four out of six facilities had electricity, one being backed by a solar power system. Network connectivity was limited in all facilities which also hindered the use of ZM-EIR in real-time.

All district offices observed had one supervisor on-site with access to a computer / laptop in the office which enables access to web-based ZM-EIR dashboards. The supervisors reported to engage in daily monitoring of the vaccinators whereby they track the vaccinators' attendance, location, and registration. No other use of ZM-EIR was reported or observed.

STUDY AIM 4 (IMPACT): To determine whether ZM-EIR usage (dose response high use, moderate use, and low use) in 27 districts of Sindh province is associated with significant differences in the proportion and timeliness of children 12-23 months fully immunized (BCG, DTP3, PCV/OPV3, Measles1) from 2014 to 2019.

Objective 4.1 Compare the proportion of children 12-23 months whose caregivers report receiving (both reported and from card) BCG, DTP3, PCV/OPV3, and Measles1 immunization in MICS-6 2018-19 to those who reportedly received BCG, DTP3, PCV/OPV3, Measles1 in MICS-5 2014 (full immunization)

The vaccine-wise coverage across this evaluation's time frame of consideration (2017-2019) reflects that since its inception for scale up in the province and phase-wise implementation in all districts; increases were observed for Penta III and Measles I vaccines (Figure 7), with the highest increments (12%) observed in Measles I coverage (the last EPI schedule-based vaccine)1. Detailed Vaccine-wise and district wise results for BCG-Penta III and Measles I are provided in annexures. The prevalence of full immunization among children 12-23 months, defined as being vaccinated for BCG, 3 doses of Pentavalent, pneumococcal and polio, and measles vaccine, went up from 5% to 15.1% between 2014 and 2019 on average by district. See details below in equity analyses.

Objective 4.2 Compare the proportion of children 12-23 months who did not receive DPT 1 between MICS-6 2018-19 and MICS-5 2014 (zero dose vaccination)

The prevalence of zero dose children 6-23 months, defined as not receiving at least one dose of Pentavalent, went down from 38.7% to 24.7% between 2014 and 2019. See details below in equity analyses.

FIGURE 7. VACCINATION COVERAGE AMONG CHILDREN 12-24 MONTHS OF AGES IN SINDH FROM 2014 MICS -2019 MICS

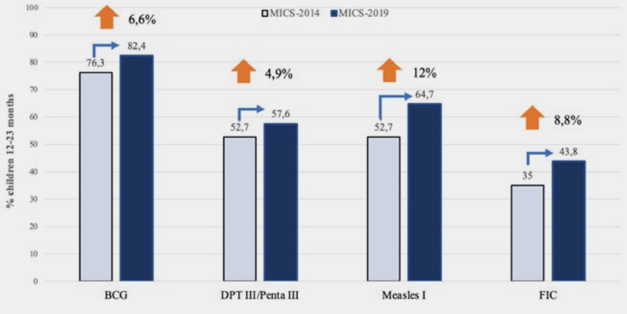

1 The EPI program in Sindh had Measles I as the last vaccine as per the child vaccination schedule till 2018. Subsequently Measles II has been added as made part of the EPI schedule, however, given the evaluation's considered time frame of 2017-2019, the quantitative analyses focused on Measles I.

FIGURE 8 PROPORTION OF CHILDREN 12-23 MONTHS IN SINDH WHO RECEIVED TIMELY IMMUNIZATION BY ANTIGEN IN 2014 AND 2019 MICS SURVEY

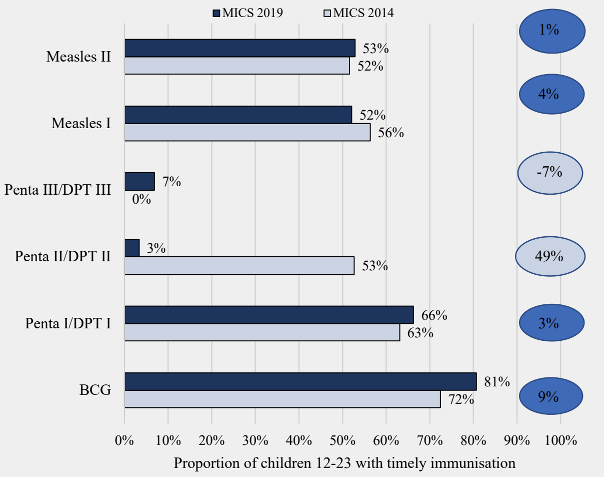

Objective 4.3 Compare the proportion of children 12-23 months whose caregivers report receiving on time (from vaccination card) BCG, DTP3, PCV/OPV3, and Measles1 immunization in MICS-6 2018-2019 to those who reportedly received BCG, DTP3, PCV/OPV3, Measles1 in MICS-5 2014 (timeliness)

Vaccination timeliness was assessed by antigen by comparing the proportion of children 12-23 months immunized across MICS surveys in 2014 and 2019 (Figure 9). Findings suggest that improvements in timeliness were achieved for BCG (+8.3%), Penta I (+3.2%) and Measles II (+1.3) vaccines. Reductions were seen for Penta II/DPT II (-49%) and Measles I (-4%). DPTII / Penta II is a challenging indicator for timeliness as it has the most stringent of conditions - needs to be exactly midway between I and III.

STUDY AIM 5 (IMPACT). To determine whether ZM-EIR implementation in 27 districts of Sindh province is associated with significant differences in the proportion of children 12-23 months in the poorest and poorer socioeconomic strata (and other dimensions of equity) fully immunized (BCG, DTP3, PCV/OPV3, Measles1) from 2014 to 2019.

As reported above, prevalence of full immunization among children 12-23 months, defined as being vaccinated for BCG, 3 doses of Pentavalent, pneumococcal and polio, and measles vaccine, went up from 5% to 15.1% between 2014 and 2019. From an equity perspective, increases were similar across the board in terms of wealth and educational statuses and urban rural residence.

Objective 5.1 Compare the estimates of zero dose immunization, full immunization, and timeliness of immunizations MICS- 6 2018-2019 and MICS 5 2014 across the wealth quintiles, by the child's gender, levels of mother's / father's education, and other sociodemographic characteristics.

The prevalence of zero dose children 6-23 months, defined as not receiving at least one dose of Pentavalent, went down from 38.7% to 24.7% between 2014 and 2019. Similarly, the changes were similar across the board in terms of sociodemographic characteristics.

Timeliness of the different antigens was assessed using regression models (logit) with the outcome variable of receiving an antigen within the appropriate timeframe. These models are adjusted for wealth, education, residence, and number of children in households and 95% confidence intervals adjusted for clustering at the community level (PSU) are presented. Timeliness increased by 1.5-2 times for BCG, Penta1, Penta2, Measles1 and Measles2, while the models show a decrease for Penta3 and Penta3. 1. PCV was not available widely in 2014 and hence the dramatic increase in 2019.

TABLE XX INTERVENTION EFFECT ON TIMELINESS OF VACCINES FROM THE MICS SURVEYS (N=5848)

	Odds ratios	P value	95% CI	
**BCG**	2.29***	<0.001	1.91***	2.74***
**Penta1**	1.55***	<0.001	1.28***	1.88***
**Penta2**	1.56***	<0.001	1.29***	1.90***
**Penta3**	0.11***	<0.001	0.07***	0.17***
**PCV1**	5.93***	<0.001	4.62***	7.60***
**PCV2**	5.43***	<0.001	4.18***	7.05***
**PCV3**	5.58***	<0.001	4.10***	7.60***
**Measles1**	1.37**	0.01**	1.08**	1.74**

TABLE 6 PREVALENCE OF ZERO DOSE AMONG CHILDREN 12-23 MONTHS ACROSS TWO ROUNDS OF MICS BY SOCIO-DEMOGRAPHIC CHARACTERISTICS

	Prevalence 2014	95% Ci		Prevalence 2019	95% CI	
**Overall**	5.0%	3.9%	6.1%	15.1%	13.5%	16.7%
**Wealth categories**						
**Poorest**	2.6%	1.4%	3.8%	8.5%	5.8%	11.1%
**Second**	3.1%	1.8%	4.3%	13.8%	10.7%	17.0%
**Middle**	6.8%	4.5%	9.2%	13.9%	10.8%	16.9%
**Fourth**	5.5%	2.9%	8.2%	19.7%	15.8%	23.6%
**Richest**	8.6%	4.0%	13.3%	22.6%	18.4%	26.8%
**Mobile access**						
**No mobiles**	3.3%	1.2%	5.5%	11.7%	9.9%	13.5%
**Mobile access present**	5.3%	4.1%	6.5%	20.0%	17.4%	22.7%
**Education - head of household**						
**None/Pre-primary**	5.0%	3.1%	6.9%	12.5%	10.3%	14.7%
**Primary**	3.2%	1.8%	4.6%	15.3%	11.3%	19.3%
**Middle**	3.7%	1.2%	6.1%	16.6%	10.7%	22.4%
**Secondary**	7.7%	4.0%	11.4%	18.8%	14.4%	23.1%
**Higher/HigherSec**	6.3%	3.5%	9.1%	19.0%	15.2%	22.9%
**Maternal education**						
**None/Pre-primary**	4.0%	2.8%	5.2%	12.0%	10.1%	13.9%
**Primary**	4.4%	2.3%	6.6%	22.2%	17.6%	26.8%
**Middle**	5.5%	0.9%	10.1%	18.0%	11.2%	24.9%
**Secondary**	5.7%	2.4%	9.0%	17.9%	12.8%	23.0%
**Higher/HigherSec**	9.7%	5.3%	14.1%			
**Residence**						
**Urban**	7.0%	5.0%	9.1%	18.2%	15.7%	20.7%
**Rural**	3.2%	2.3%	4.1%	12.5%	10.4%	14.5%
**Sex of child**						
**Male**	5.8%	4.1%	7.4%	15.3%	13.2%	17.3%
**Female**	4.2%	3.0%	5.4%	15.0%	12.7%	17.2%

TABLE 7 PREVALENCE OF ZERO DOSE AMONG CHILDREN 12-23 MONTHS ACROSS TWO ROUNDS OF MICS BY SOCIO-DEMOGRAPHIC CHARACTERISTICS

	Prevalence 2014	95% Ci		Prevalence 2019	95% CI	
**Overall**	38.7%	36.3%	41.1%	24.7%	23.1%	26.3%
**Wealth categories**						
**Poorest**	61.3%	57.3%	65.2%	36.9%	33.1%	40.6%
**Second**	49.4%	45.4%	53.4%	27.3%	24.4%	30.2%
**Middle**	32.1%	28.3%	35.8%	22.6%	19.7%	25.6%
**Fourth**	22.3%	17.7%	26.9%	19.6%	16.4%	22.8%
**Richest**	17.0%	12.5%	21.5%	11.0%	8.1%	14.0%
**Mobile access**						
**No mobiles**	53.8%	49.0%	58.6%	30.1%	28.0%	32.2%
**Mobile access present**	36.3%	33.9%	38.8%	16.5%	14.4%	18.5%
**Education - head of household**						
**None/Pre-primary**	48.1%	44.7%	51.6%	31.1%	28.8%	33.4%
**Primary**	41.8%	37.6%	46.0%	21.5%	18.0%	25.0%
**Middle**	31.9%	25.4%	38.3%	18.3%	13.9%	22.8%
**Secondary**	28.9%	24.1%	33.6%	19.8%	16.3%	23.4%
**Higher/HigherSec**	25.7%	21.8%	29.6%	15.7%	12.7%	18.8%
**Maternal education**						
**None/Pre-primary**	52.4%	49.3%	55.5%	30.8%	28.7%	32.9%
**Primary**	31.1%	27.0%	35.1%	16.4%	13.0%	19.9%
**Middle**	21.0%	13.8%	28.3%	14.2%	9.3%	19.0%
**Secondary**	21.2%	16.0%	26.3%	16.1%	11.8%	20.4%
**Higher/HigherSec**	13.6%	9.2%	17.9%			
**Residence**						
**Urban**	25.3%	22.5%	28.0%	19.1%	17.1%	21.2%
**Rural**	51.0%	47.8%	54.2%	29.4%	27.1%	31.7%
**Sex of child**						
**Male**	38.0%	35.1%	40.8%	24.9%	22.9%	27.0%
**Female**	39.5%	36.4%	42.5%	24.4%	22.3%	26.6%

STUDY AIM 6 (IMPACT). To determine the incremental cost effectiveness of ZM-EIR as compared to status quo in 27 districts of Sindh province from a program perspective from October 1, 2017- December 31, 2018

Objective 6.1 Using an ingredients approach and drawing from budget and expense summary by category for the October 1, 2017- December 31, 2018 analytic time horizon, estimate the economic costs of ZM-EIR implementation, including program development, start-up and on-going implementation.

Overall program costs from October 1, 2017 to February 28, 2019 were $3.27 million USD. The Start-up phase – characterized by one-time activities required to initiate the program -- occurred from October 2017 to February 28, 2018 and cost an estimated $2.21 million USD. Nearly two-thirds of start-up costs were comprised of equipment and personnel. The former, included costs associated with two servers, 47 laptops, and 3,837 phones and accessories; items procured by donors directly and donated to the project. The lifespan is estimated at five years and plans are in place to begin replacement in November 2022.

By the end of February 2018, ZM-EIR had been rolled out across all 27 districts in Sindh. Implementation costs were assessed for a 12-month window from March 2018 to February 2019. Table 8 presents costs associated with this 12-month period of implementation, including annualized start-up phase costs. The leading cost drivers were personnel including technical and field staff (53%), refresher training and support travel (11%), equipment (10%), airtime (8%), and initial training costs (6%). Initial training spanned for 2-3 days per town and 3-6 towns per district, and included travel, personnel costs, and per diems for trainees. Airtime costs included mobile airtime used to support program monitoring and implementation. Replacement costs were estimated to account for equipment provided by donors and noted above. A 10% indirect cost was incorporated across all reported costs.

TABLE 8 SUMMARY OF ANNUALIZED START-UP AND ONE-YEAR IMPLEMENTATION SUPPORT COSTS FOR MARCH 2018 TO FEBRUARY 2019 IN 2019 USD

Cost category	Description	Total	%
**Annualized Capital costs**			
**Application development and optimization**		$11 548	1%
**Training guidelines development**	Per diem/ Hotel stay	$28	0%
	Printing/training Material	$2 308	0%
**Initial training & travel**	Travel	$19 370	1%
	Per diem/ Hotel stay	$51 103	3%
	Venue	$2 170	0%
	Printing/training Material	$9 210	1%
	Rental cars	$11 485	1%
**Equipment Procurement**	Mobile procurement (hardware)	$136 913	9%
	Server and related cost	$4 734	0%
	Laptops	$18 481	1%
	**Sub-total capital costs**	**$267 351**	**17%**
**Recurrent costs and activities**			
**Personnel, building, and other support costs**	Personnel	$822 886	53%
	Operational Costs	$21 998	1%
	Misc. Cost	$20 575	1%
**Refresher training & travel**	Travel	$18 386	1%
	Per diem/ Hotel stay	$111 682	7%
	Printing/training Material	$13 442	1%
	Rental cars	$37 818	2%
**Application optimization**		$38 586	3%
**QR code printing**		$64 343	4%
**SMS reminders**		$7 604	0%
**Airtime costs**		$118 740	8%
	**Sub-total recurrent costs and activities**	**$1 276 062**	**83%**
	**Grand Total**	**$1 543 413**	**100%**

TABLE 9 INCREMENTAL CHANGES IN COVERAGE BY ANTIGEN 2014 MICS-5[1] AND 2019 MICS-6[2]

Antigen	MICS-5	MICS-6	Change in Coverage	Coverage estimates inputted into LiST					
	2014	2019	2019-2014	2014	2015	2016	2017	2018	2019
**BCG – 1 dose**	77,4%	82,4%	5,0%	77,4%	78,4%	79,4%	80,4%	81,4%	82,4%
**Polio – 3 doses**	63,5%	57,9%	-5,6%	63,5%	62,4%	61,3%	60,1%	59,0%	57,9%
**Pentavalent**	55,3%	54,7%	-0,6%	55,3%	55,2%	55,1%	54,9%	54,8%	54,7%
**DPT - 3 doses**	55,3%	54,7%	-0,6%	55,3%	55,2%	55,1%	54,9%	54,8%	54,7%
**Hib – 3 doses**	55,3%	54,7%	-0,6%	55,3%	55,2%	55,1%	54,9%	54,8%	54,7%
**HepB – 3 doses**	55,3%	54,7%	-0,6%	55,3%	55,2%	55,1%	54,9%	54,8%	54,7%
**Pneumococcal - Three doses**	27,6%	52,7%	25,1%	27,6%	32,6%	37,6%	42,7%	47,7%	52,7%
**Measles – 1 dose**	58,6%	53,3%	-5,3%	58,6%	57,5%	56,5%	55,4%	54,4%	53,3%

Hib- H. influenzae type b; HepB-Hepatitis B

1 https://mics-surveys-prod.s3.amazonaws.com/MICS5/South%20Asia/Pakistan%20%28Sindh%29/2014/Final/Pakistan%20%28Sindh%29%20 2014%20MICS_English.pdf
2 https://mics-surveys-prod.s3.amazonaws.com/MICS6/South%20Asia/Pakistan%20%28Sindh%29/2018-2019/Survey%20findings/Pakistan%20 2018-19%20MICS%20%28Sindh%29_English.pdf

Objective 6.2 Draw from MICS analyses to determine incremental changes in coverage for individual vaccines (BCG, DTP3, PCV/OPV3, and Measles1)

Incremental changes in vaccination coverage by antigen were estimated from the 2014 and 2019 MICS surveys. The difference in point estimates of coverage from 2014 and 2019 MICS surveys were taken by antigen and then divided over a five-year window of time using the 2014 MICS coverage estimate as the baseline. For example, the coverage of a single dose of BCG increased 5% from 77.4% in 2014 to 82.4% in 2019. Assuming a 2014 base year coverage of 77.4% and a linear increase in coverage over time, coverage increased by 1% annually for 5-years through 2019. Table 9 summaries coverage estimates inputted into the Lives Saved Modelling tool and used to generated estimates of lives saved among children 12-23 months across Sindh by year and antigen.

Objective 6.3 Use the lives saved tool to model the incremental lives saved associated with changes in immunization outcomes for the calendar year 2019 as compared to 2014

Table 10 summarizes incremental lives saved for 2017-2019 following the rollout of ZM across Sindh. Despite the slight decline in immunization coverage for pentavalent, the population increase in the number of children immunized even at lower rates still corresponded to an incremental increase in lives saved for Hib-3 and DTP-3. The latter comprised 41% and 12% of total lives saved in 2018, respectively (Figure 9).

FIGURE 9. PROPORTION OF LIVES SAVED BY ANTIGEN IN 2018

Antigen	2017	2018	2019	TOTAL
**BCG - Single dose**	0	0	0	0
**Polio - Three doses**	0	0	0	0
**DTP - Three doses**	96	132	138	366
**Hib-Three doses**	386	463	482	1330
**HepB - Three doses**	0	0	0	0
**Pneumococcal - Three doses**	251	366	410	1027
**Measles - Single dose**	118	172	176	466
**TOTAL**	851	1 133	1 205	3 189

TABLE 10 INCREMENTAL LIVES SAVED FROM 2017-2019 AMONG CHILDREN 12-23 MONTHS IN SINDH

Antigen	2017	2018	2019	TOTAL
BCG - Single dose	0	0	0	0
Polio - Three doses	0	0	0	0
DTP - Three doses	96	132	138	366
Hib-Three doses	386	463	482	1330
HepB - Three doses	0	0	0	0
Pneumococcal - Three doses	251	366	410	1027
Measles - Single dose	118	172	176	466
TOTAL	851	1 133	1 205	3 189

6.1.2 Hib- H. influenzae type b; HepB-Hepatitis B

Objective 6.4 Estimate the incremental cost per life saved of ZM-EIR implementation in calendar years 2019-20 versus status quo of 2014 (pre-implementation)

Incremental cost effectiveness ratios rely on the assumption that incremental changes in lives saved were attributed to ZM-EIR and no other factors including health systems strengthening, or other immunization initiatives. In addition to this limitation, efforts to determine the incremental cost-effectiveness are hampered by the absence of a true 'comparator' or control area or population with data on both costs and consequences. These significant limitations aside, Table 11 aims to consider the total program costs for 12 months of implementation across Sindh including annualized Start-up costs and 12-month Implementation costs along with lives saved and DALYs averted. Program costs include annualized Start-up costs and Implementation costs for the March 1, 2018 to February 28, 2019. Start-up costs were annualized using an annualization factor [((1+r)^n) - 1] / [r(1+r)^n], where r = discount rate of 3% and n = useful life of the capital item. All capital items, including initial training, servers, and mobile phones were assumed to have a life expectancy of five years; a window of time consistent with the observed useful life of these items in the field.

Results suggests that the total annualized program cost for 12-months in 2019 USD was $1.54 million USD corresponding to a cost-per life saved of $1,362 USD, and a cost per DALY averted of $50.45.

TABLE 11 ANNUALIZED PROGRAM COSTS (2019 USD) AND INCREMENTAL LIVES SAVED FOR 12 MONTHS (MARCH 2018 TO FEBRUARY 2019) OF IMPLEMENTATION ACROSS SINDH

Program costs	Incremental lives saved	Cost per life saved	DALYs averted	Cost per DALY averted
$1,543,413.00	1,133	$1,362.24	30,591	$50.45

TABLE 12 LEAGUE TABLE COMPARING COST PER LIFE SAVED WITH ALTERNATIVE RESOURCE USES

Comparison with other interventions for children	Description	Cost per DALY averted	Source
**Treat severe malaria with artesunate versus quinine, Africa & Southeast Asia**	Use of parenteral artesunate to treat children with severe malaria in Africa and Southeast Asia	$5	Disease Control Priorities Project 3rd Edition (DCP-3)
**Kilkari**	Direct to beneficiary to beneficiary mobile health intervention in India	$12-$29 USD	LeFevre AE et al (2022)
**Zinc added to oral rehydration therapy**	Used zinc as adjunct therapy to standard treatment of acute childhood diarrhea	$10-50	DCP-3
**Community management severe-acute malnutrition**	Community-based therapeutic care: Diagnosis, RUTF (Ready-to-Use-Therapeutic Food), supplements, in-patient treatments, out-patient visits, weekly follow ups	$25–40	DCP-3
**Maternal and neonatal care at home**	Maternal and neonatal services delivered at home, with community mobilization and health system strengthening	$13–126	DCP-3
**ZM suite of digital interventions**		$51	
**Rotavirus immunization in India**		$66	Verguet S et al (2013)
**Original EPI-6 plus Hepatitis B**	Expanded Program of Immunization with six vaccines	$103	DCP-3
**Pneumococcus and rotavirus Low Income Countries**	Implementing pneumococcus and rotavirus vaccination program; low income countries are eligible to procure vaccines from Gavi at low prices	$103	DCP-3
**Handwashing BCC (behavior change communications)**	Increase hand-washing after handling child stool and disposal of stool in latrines	$90-225	DCP-3
**Access to modern contraceptives**	Universal access to modern contraceptives	$150–300	DCP-3
**Mother's groups to**			
**improve maternal/neonatal health**	Mother’s group to improve maternal and neonatal health in Bangladesh and India; converted from LYS	$150-1,000	DCP-3
**Comprehensive nutrition package (all interventions Lancet 2013)**	Interventions addressing undernutrition and micronutrient deficiencies in women and children in 34 countries	$353	DCP-3
**HiB and rubella added to EPI in LICs**	Introduction of HiB and rubella into DPT-Hepatitis B program	$368–768	DCP-3
**MAMA South Africa**	Maternal mobile health information messages	$200-$1,985	LeFevre AE et al (2018)
**HiB vaccine - India, Gujarat**		$612.00	Clark et al (2013)
**Breastfeeding**	Effect on diarrhea in South Asia (World Bank regions)	$1,736.00	Keusch et al (2006)
**C-section, all LMICs**	Treating obstructed labor with Caesarean delivery in 49 countries, across multiple regions, identified by the WHO	$1,600–2,600	DCP-3
**Pneumococcus and rotavirus (market price, upper-mid IC)**	Adding pneumococcus and rotavirus vaccination to standard vaccinations	$2,018	DCP-3
**Rural water supply/sanitation, LIC**	Piped water supply and sewer connection, 98% coverage	$2,200	DCP-3

7. Discussion

This external evaluation of the ZM-EIR package of electronic tools and applications for childhood immunization in Sindh, Pakistan highlights important outcomes, insights and recommendations across stakeholder groups in all six study aims. The implementation of ZM-EIR in Sindh Province from 2017 to 2019 is estimated to have saved over 3,000 lives among children 12 to 23 months through the more efficient delivery of timely and complete childhood vaccines. The estimated cost to implement this suite of tools is $1,362 USD per life saved and $50.45 per DALY averted.

The successful implementation of ZM-EIR in Sindh Province contributed to a 10-percentage point increase in the prevalence of fully-immunized children, from 5% before implementation to 15.1% of 12 to 23 month children fully-immunized after 3 years of implementation. The rate of on-time vaccination (timeliness) improved for most but not all antigens in the recommended childhood vaccination schedule. The prevalence of zero-dose children between 6 and 23 months of age decreases from 38.7% pre-implementation to 24.7% after implementation. These positive results for the ultimate beneficiaries, the children under 2 years in Sindh Province, demonstrate that increased access to immunization program data contributes to improved health outcomes.

7.1 OVERALL PERCEPTIONS & EXPERIENCES WITH ZM-EIR BY STAKEHOLDER GROUP

The qualitative components of the evaluation highlight some of the perceptions and experiences of the primary users of the ZM-EIR suite of digital health interventions, namely the caregivers, vaccinators, managers and supervisors who have access to the data and information through various components of the ZM-EIR package. Overall, all stakeholder groups interviewed had a favorable impression about the ZM-EIR suite of interventions. Provincial managers felt that the real-time access to vaccination data facilitated longitudinal monitoring of vaccination coverage over time and across geographic areas. District managers highlighted the importance of vaccinator tracking and attendance monitoring, the ability to track coverage, zero-dose and defaulters as well as the ability to access daily and monthly reports to optimize interactions with vaccinators and other EPI stakeholders. Vaccinators, who use the app daily beginning with marking their attendance as the first task of the day, expressed their satisfaction with ZM-EIR features, specifically for generating actionable defaulter and zero-dose lists. The robust and varied methods employed for this study allow for qualitative insights for each stakeholder group with user perceptions and experiences that provide a nuanced view of how the ZM-EIR suite of interventions can be improved for the future. For all immunization program stakeholders, the existing infrastructure of internet connectivity, electricity, and mobile network coverage in the field and at some health facilities limited the availability and sustained use of the ZM-EIR tools, reports and data. This is a common experience across most digital health interventions in LMICs.

7.1.1 CAREGIVER PERCEPTIONS & EXPERIENCES WITH ZM-EIR

Caregivers are the ZM-EIR stakeholders responsible for facilitating and allowing a child's direct access to vaccination services. The ZM-EIR suite of tools are designed to give caregivers greater access to information about their child's immunization schedule, reminders, and a direct line of communication for questions and concerns, as well as improved service delivery experiences as a result of the vaccinator's access and use to ZM-EIR tool and program data. The timeliness and overall efficiency of immunization coverage is perceived to be improved through SMS reminders targeted at caregivers. However, given the cultural norms of limited physical mobility for women and male-female segregation that increasingly persist in rural contexts of the districts under study, women's involvement in direct communication for their children's vaccination purposes is limited. They do not give their phone numbers to male vaccinators and male vaccinators do not request this type of personal information. Female caregivers expressed preference for female vaccinators.

From the lens of the caregivers, the number of vaccinations received has increased among the poorer, less educated, and rural social stratum. However, the reasons are not directly attributed to ZM-EIR. According to interviewees, in recent years more vaccination centers have been established and a positive change in public attitude towards vaccinations has led to more public awareness and recognition of the benefits of vaccination for the health of the community. This increase in awareness has also led to more receptivity to home visits by healthcare workers who were not always welcomed by the rural communities a few years ago.

7.1.2 VACCINATOR PERCEPTIONS & EXPERIENCES WITH ZM-EIR

Vaccinators use the ZM-EIR interface to directly enter data on the vaccines administered as well as access reports and tools designed to increase their accountability, access and use of data and decision-making aids. Some vaccinators perceived resistance from caregivers, experience increased record-keeping burden due to dual reporting and some report spending personal funds for mobile data usage (once they exceed the limit of the maximum data package provided to them). In addition, the timeframe for this study was prior to the setting of compliance targets by the Government of Sindh. These considerations may explain why some vaccinators do not use the app for data entry at the point of service delivery and why compliance and regular use of the system to enter data was below 60% overall. Since compliance targets were set, it has reportedly increased to 80% overall except for periods of time when vaccinators are on strike. However, vaccinators do report having greater comfort and digital literacy to interact with and use the various features of ZM-EIR and their compliance and engagement with the tools increased over the study period. Despite there being much fewer of them (average proportion of 15% female to 85% male), female vaccinators exhibited greater use of ZM-EIR tools with higher compliance than their male counterparts.

7.1.3 MANAGEMENT PERCEPTIONS & EXPERIENCES WITH ZM-EIR

District and provincial managers and supervisors have access to the data in the ZM-EIR dashboard and can monitor the compliance and achievements of vaccinators in their catchment areas. The supervisors' responsibilities include monitoring and providing support to vaccinators, checking vaccine stock, and planning and conducting field visits. Despite all these critical functions, their role in access and use of ZM-EIR data has been relatively limited compared to other users. Even with their limited use of ZM-EIR app to monitor the activities of vaccinators in their respective district areas, newer and younger supervisors were able to use the app comfortably, while older supervisors have found the app difficult and less engaging.

TABLE 13 COMPARISON OF ZM AND IDEAL EIR CHARACTERISTICS AND FUNCTIONALITIES

Features of Zindagi Mehfooz -EIR (ZM-EIR)	PATH (2021) Ideal requirements of EIR
ZM-EIR is a registry to **enroll all newborns** or never-vaccinated children with a **unique ID / QR code** assigned to each woman and child; a web-interface **allows data visualization, mobile-based data entry and access for vaccinators**	**Registration and search features:** Enrollment at birth, Client management, Unique and unequivocal ID
Registration includes all relevant **demographic and location data of patient** and facility with **geo-location; barcoded vaccine vials** linked with vaccine record; **Offline mode** for areas with low mobile/ data coverage **[not linked to other health areas]**	**Patient records:** Individual demographic data, Vaccine event data, Nonroutine (campaign and outreach) vaccine events, and linkages to other health areas
Immunization **decision support system** guides vaccinators on routine and catch-up immunizations; **Defaulter reports** for vaccinators; Interactive **SMS reminders** for parents/caregivers	**Vaccination monitoring and follow-up:** Clinical decision support, Identification of under-vaccinated children/missed vaccination, Reminder and recall messages
**Vaccinators are registered** within the system and assigned to a **health facility registered** to a district management unit.	**Health facility registration and management**
**Vaccine stock is tracked** through number and type of vaccinations given by vaccinator, facility, and district.	**Stock management**
**Geospatial analysis** to map pockets of under-vaccinated children with exact location to enable follow-up with refusal households; Call center for complaints and **AEFI reporting**	**Data and reporting:** Data aggregation at different geographic and administrative levels, Adverse event reporting
Follows industry standards for **data security and privacy** platform **interoperable with DHIS2 and Government's EPI-MIS** deployed at **full scale** in 29 districts of Sindh Province by over 3,096 vaccinators at 1,694 public and private sector immunization clinics to enroll 3.8 million children and 1.3 million women with 31 million immunization visits recorded (as of 2021).	**Other system requirements:** Data exchange and interoperability, Offline capability, Alignment with international standards, Data privacy and security, Scalability and capacity, Usability
**ZM-EIR Additional Features Beyond Ideal EIR**	
• AI-based predictive analytics to identify children more likely to drop out<br>• SMS, call center and AI chat bot for caregiver questions, concerns, and complaints<br>• Geo-spatial data for vaccination events - geospatial data to track each child's immunization progress<br>• Real-time tracking of vaccinators with GIS tracking to improve monitoring and accountability, simultaneous supervision of multiple teams, monitor attendance and mobility of field staff<br>• Gamified videos and training for vaccinators	

7.2 ZM-EIR AS MORE THAN AN EIR

Electronic immunization registries are designed to improve the collection, analysis, and use of immunization program data to improve efficiency, equity, and coverage of immunization programs (Pancholi et al., 2020). Improved access to better data can lead to better decisions, planning and operations, which in turn will lead to better health outcomes (Werner et al., 2019). These incremental changes depend on the user experience of all stakeholders involved in the childhood immunization program with the goal to improve the number of children receiving timely and complete vaccinations. ZM-EIR is comprised of complementary digital health interventions that satisfy and go beyond the conventional definition of an electronic immunization registry (EIR). The cross-functionality large-scale implementation in Sindh Province provide an excellent opportunity for this evaluation to examine the benefits, opportunities, and areas for digital health information to realize improvements in immunization service delivery, equity, and coverage.

7.2.1 STATE OF EIR EVALUATIONS

Objective and rigorous evaluations of digital health interventions are critical to understand the value and impact of digital systems and to document lessons and experiences that will contribute to the evidence base and growing landscape of digital health implementation knowledge (Mathews et al., 2019). Evaluations of digital health should encompass technical parameters of the intervention, outcomes, usability, and cost with direct assessment and input from stakeholders to understand their definitions of success and usability (Mathews et al., 2019). Because EIRs are intended to support immunization programs with improved data-driven decision-making, the evaluation of data use is also important (Carnahan et al., 2020). Drivers of EIR use include organizational factors (supervision, support, resources, facility type, location and volume of clients), technical factors (infrastructure, electricity and internet connectivity), and behavioral factors (training, capacity and motivation of system users) (Carnahan et al., 2020). Waves of data-use related to EIRs have been documented in other LMIC settings and provide insight into the incremental process of adopting a new data collection technology into daily routines, increasing comfort and utility that lead to improved data quality, and eventual awakening to the usefulness of the data for immunization program decision-making (Werner et al., 2019). A recent study using EIR data to systematically evaluate timeliness of vaccination found mixed results with the conclusion that the overall reliance of the study on system-level data was insufficient to evaluate its impact on immunization outcomes (Dolan et al., 2022).

The ZM-EIR evaluation reliance on digital health intervention system data alongside secondary analyses of immunization coverage and equity survey data provides a more robust approach to evaluation with much clearer effects and relationships between the use of ZM-EIR and immunization outcomes. This is complemented by the qualitative research to provide a more holistic assessment of the perceptions and experiences alongside outcomes. It is also hypothesized that the EIR along with the extended functions of ZM-EIR are contributing to coverage, timeliness, completion, and zero-dose related immunization outcomes rather than the EIR alone. However, without a counterfactual it is hard to confidently draw this conclusion. Digital health interventions and applications for system strengthening in LMICs are encouraged to adopt rigorous evaluations and to share the results widely to facilitate the responsible and informed growth and adoption of established digital health interventions.

8. Recommendations

This study contributes to the evidence base of the added value and recommendations for implementing EIRs in LMICs with a retrospective analysis of pre-existing data, observational study, external surveys, interviews and both qualitative and quantitative methods to make recommendations on the necessary changes of the ZM-EIR system in Pakistan and to help guide and inform replication of similar systems in other settings. Assessments include organization, technical, and behavioral factors contributing to the success or the intervention, impact on outcomes and cost of implementation to provide a holistic picture of the ZM-EIR system, challenges, and areas for improvement.

This external evaluation presents several suggestions and areas for improvements to address some of the challenges experienced by users of ZM-EIR. The following recommendations are based on the examination of quantitative and qualitative data and hold the most promise for future improvements in immunization outcomes and EPI program strengthening in Sindh Province in other provinces in Pakistan and in other LMICs.

- There is a gap in use of the ZM-EIR tools among supervisors who are older and less able to use the computer-based system. Supervisors should have a dedicated app that can expand their ability to directly monitor vaccinators.
  Recommendation to co-design supervisor app for mobile phone and/or tablet.
- The reliance on double record-keeping needs to end to reduce the data-entry burden (double requirement to complete both paper and mobile data entry) for field vaccinators and facility staff.
  Recommendation to move towards paperless system.
- Gender dynamics, gender segregation (male vaccinators / female caregivers), education, literacy and cultural norms impact access and use by vaccinators and the ability to make direct contact with caregivers. Power, trust and relationships present challenges between vaccinators and clients of the opposite gender. Although their numbers are lower, female vaccinators have higher compliance with the ZM-EIR system than their male counterparts.
  Recommendation to engage more female vaccinators and address gender more systematically through gender analyses and planning at the start of all future ZM-EIR intervention planning.
- Limited internet connectivity, electricity and mobile network coverage at health facilities and outreach locations place constraints on the intended use of the ZM tools.
  Recommendation to engage with Ministry of ICT and MNOs to improve connectivity in poor performing districts with low ZM-EIR compliance due to poor connectivity.

9. Conclusion

Zindagi Mehfooz Electronic Immunization Registry is a suite of digital health interventions well beyond the standard definition of EIR. Key functions that extend the functionality of ZM-EIR are the mobile phone-based case management and vaccinator tracking features, SMS reminders to caregivers for upcoming and missed vaccinations, and predictive analytics to identify potential dropouts. This evaluation of ZM-EIR provides a good demonstration of how digital data collection and sharing through a unified platform can identify and reach zero-dose and under-immunized children. The integrated digital health intervention approach to immunization combines demand generation, geo-spatial data, stock management, effective sub-national data use by Provincial and District Managers with decision support and case management for service delivery by vaccinators. This evaluation suggests that a deeper understanding and background assessment of the challenges and barriers faced by caregivers and vaccinators could lead to the development of outreach, tools and expanded functionalities of ZM-EIR that address the immediate needs of the community. The vaccinators face challenges related to trust and relationships with caregivers of the opposite gender.

The start-up investments in ZM-EIR have contributed to a learning curve and experimentation that will lead to more cost-effective implementation of ZM-EIR in Pakistan as well as similar immunization data platforms in other countries. Part of the important learnings from the ZM-EIR implementation experiences stress the need to prioritize digital health enablers such as capacity, connectivity, and electricity to maximize potential and scale-up of digital health interventions. It is recommended that gender-related cultural norms related to challenges with female caregiver engagement should be addressed more systematically with gender analyses and planning at the start of any digital health intervention planning in Pakistan and elsewhere.

The implementation of ZM in Sindh Province has helped advance coverage of immunization service for children. Continued improvements and increased transparency of immunization program data can continue to have positive impacts on immunization coverage, timeliness, equity, and service delivery.

10 References

Dolan et al. (2022) Changes in on-time vaccination following the introduction of an electronic immunization registry, Tanzania 2016-2018: interrupted time-series analysis. BMC Health Services Research 22:1175 https://doi.org/10.1186/s12913-022-08504-2

Carnahan, E., Ferriss, E., Beylerian, E., Mwansa, F.D., Bulula, N., Lyimo, D., Kalbarczyk, A., Labrique, A.B., Werner, L. and Shearer, J.C. 2020. Determinants of facility-level use of electronic immunization registries in Tanzania and Zambia: an observational analysis. Global Health: Science and Practice. 8(3):488-504. [link]

Mathews, S.C., McShea, M.J., Hanley, C.L., Ravitz, A., Labrique, A.B. and Cohen, A.B., 2019. Digital health: a path to validation. NPJ digital medicine. 2(1):1-9. [link]

PAHO. 2017. Electronic Immunization Registry: Practical Considerations for Planning, Development, Implementation and Evaluation. Washington, DC: PAHO. [link]

Pakistan EPI Program. 2022. Case Study: Integrated Services Delivery (ISD) Model, indeed, proves to be a success in Pakistan! May, 2022. Government of Pakistan Ministry of National Health Service Regulations and Coordination & Expanded Program on Immunization. [link]

Pancholi J, Birdie R, Guerette J, Chritz S, Sampath V, Crawford J. 2020. Landscape Analysis of Electronic Immunization Registries: Lessons Learned from a Landscape Analysis of EIR Implementations in Low and Middle Income Countries. Seattle, WA: VillageReach. [link]

PATH. 2021. Digital Square Electronic Immunization Registries in Low- and Middle-Income Countries. Seattle: PATH. [link]

Werner, L., Seymour, D., Puta, C. and Gilbert, S. 2019. Three waves of data use among health workers: the experience of the Better Immunization Data Initiative in Tanzania and Zambia. Global Health: Science and Practice. 7(3):447-456. [link]

11. Annexes

11.1 ANNEX A: STUDY AIM AND OBJECTIVES

STUDY AIM 1 (REACH). DETERMINE BARRIERS AND FACILITATORS TO PROGRAM INITIATION

- Objective 1.1 (Program reach) Draw from ZM-EIR system data and program records on vaccinator registration and training and Government records on numbers of vaccinators to determine proportion of eligible vaccinators trained by the program and registered within ZM-EIR from 2017 to 2019 in alignment with MICS 6 from 2018-2019 and MICS 5 in 2014
- Objective 1.2 (Provider use) Conduct secondary analysis of system generated data to determine what proportion of vaccinators trained to use ZM-EIR upload details for at least 1 client in Sindh
- Objective 1.3 (Stakeholder perceptions) Conduct in-depth interviews with vaccinators, clients, and supervisors and other key stakeholders) to understand perceptions about the benefits of ZM-EIR and effects on immunization service delivery (decision-support algorithm, registry, SMS alerts and reminders)

STUDY AIM 2 (REGISTRY COVERAGE). DETERMINE PROPORTION OF CHILDREN AMONGST THOSE ELIGIBLE AT A POPULATION LEVEL CONTAINED WITHIN THE ZM-EIR REGISTRY

- Objective 2.1 Model estimated number of children eligible for vaccination in study area over time using census data by district based on the ZM-EIR implementation period per district from 2017 to 2019 in alignment with MICS 6 from 2018-2019 and MICS 5 in 2014
- Objective 2.2 Draw from ZM-EIR system generated data to determine the number of unique children contained within ZM-EIR vaccination registry
- Objective 2.3 Conduct in-depth interviews and observations with vaccinators, clients, and supervisors (and other key stakeholders) to understand their views on differences in registry coverage observed and effects on uptake of immunization services

STUDY AIM 3 (DATA USE). DETERMINE THE AVAILABILITY AND USE OF ZM-EIR DATA ACROSS KEY STAKEHOLDER GROUPS

- Objective 3.1 Conduct secondary analyses of system generated data, reports and dashboards to determine frequency of ZM-EIR data use by supervisors, managers, and Government staff at all levels.
- Objective 3.2 Conduct in-depth interviews with key stakeholders at the district and provincial levels as well as ZM-EIR staff to understand differences in newly available data (disaggregated gender data, geographical data, GIS analysis, vaccinator monitoring data) and data use patterns, perceptions on link between data use and immunization coverage, completion, and timeliness.
- Objective 3.3 Conduct direct observations of immunization services to understand the broader context within which implementation is occurring and to identify barriers to data entry and use.

STUDY AIM 4 (IMPACT): TO DETERMINE WHETHER ZM-EIR USAGE (DOSE RESPONSE HIGH USE, MODERATE USE, AND LOW USE) IN 27 DISTRICTS OF SINDH PROVINCE IS ASSOCIATED WITH SIGNIFICANT DIFFERENCES IN THE PROPORTION AND TIMELINESS OF CHILDREN 12-23 MONTHS FULLY IMMUNIZED (BCG, DTP3, PCV/OPV3, MEASLES1) FROM 2014 TO 2019.

- Objective 4.1 Compare the proportion of children 12-23 months whose caregivers report receiving (both reported and from card) BCG, DTP3, PCV/OPV3, and Measles1 immunization in MICS-6 2018-19 to those who reportedly received BCG, DTP3, PCV/OPV3, Measles1 in MICS-5 2014 (full immunization).
- Objective 4.2 Compare the proportion of children 12-23 months who did not receive DPT 1 between MICS-6 2018-19 and MICS-5 2014 (zero dose vaccination).
- Objective 4.3 Compare the proportion of children 12-23 months whose caregivers report receiving on time (from vaccination card) BCG, DTP3, PCV/OPV3, and Measles1 immunization in MICS-6 2018-2019 to those who reportedly received BCG, DTP3, PCV/OPV3, Measles1 in MICS-5 2014 (timeliness).

STUDY AIM 5 (IMPACT). TO DETERMINE WHETHER ZM-EIR IMPLEMENTATION IN 27 DISTRICTS OF SINDH PROVINCE IS ASSOCIATED WITH SIGNIFICANT DIFFERENCES IN THE PROPORTION OF CHILDREN 12-23 MONTHS IN THE POOREST AND POORER SOCIOECONOMIC STRATA (AND OTHER DIMENSIONS OF EQUITY) FULLY IMMUNIZED (BCG, DTP3,PCV/OPV3, MEASLES1) FROM 2014 TO 2019.

- Objective 5.1 Compare the estimates of zero dose immunization, full immunization, and timeliness of immunizations MICS- 6 2018-2019 and MICS 5 2014 across the wealth quintiles, by the child’s gender, levels of mother’s / father’s education, and other sociodemographic characteristics.

STUDY AIM 6 (IMPACT). TO DETERMINE THE INCREMENTAL COST EFFECTIVENESS OF ZM-EIR AS COMPARED TO STATUS QUO IN 27 DISTRICTS OF SINDH PROVINCE FROM A PROGRAM PERSPECTIVE FROM OCTOBER 1, 2017- DECEMBER 31, 2018

- Objective 6.1 Using an ingredients approach and drawing from budget and expense summary by category for the October 1, 2017- December 31, 2018, analytic time horizon, estimate the economic costs of ZM-EIR implementation, including program development, start-up, and on-going implementation.
- Objective 6.2 Draw from MICS analyses to determine incremental changes in coverage for individual vaccines (BCG, DTP3, PCV/OPV3, and Measles1)
- Objective 6.3 Use the lives saved tool to model the incremental lives saved associated with changes in immunization outcomes for the calendar year 2019 as compared to 2014
- Objective 6.4 Estimate the incremental cost per life saved of ZM-EIR implementation in calendar years 2019-20 versus status quo of 2014 (pre-implementation)
- Objective 6.5 Conduct probabilistic sensitivity analyses to identify key cost drivers and assess uncertainty.

11.2 ANNEX B. IMMUNIZATION SCHEDULE IN PAKISTAN AND DEFINITION OF FULL BASIC IMMUNIZATION

Immunization schedule		Definition of full basic immunization, by age group	
**Age**	Immunizations	Age group	Requirements for full basic immunization
**At birth**	BCG+ Polio 0	0–5 weeks	BCG, Polio 0
**6 weeks**	Penta 1 + Polio 1	6–9 weeks	BCG, Polio 0, Polio 1, Penta 1
**10 weeks**	Penta 2 + Polio 2	10–13 weeks	BCG, Polio 0, Polio 1, Polio 2, Penta 1, Penta 2
**14 weeks**	Penta 3 + Polio 3	14 weeks– 8 months	BCG, Polio 0, Polio 1, Polio 2, Polio 3, Penta 1, Penta 2, Penta 3
**9 months**	Measles 1	9–11 months	BCG, Polio 0, Polio 1, Polio 2, Polio 3, Penta 1, Penta 2, Penta 3, Measles 1
**12–15 months**	Measles 2	12 months– 23 months	BCG, Polio 0, Polio 1, Polio 2, Polio 3, Penta 1, Penta 2, Penta 3, Measles 1, Measles 2

11.3 ANNEX C: DEFINITIONS FOR EXAMINING ROUTINE IMMUNIZATION TIMELINESS

Vaccine	Validity as per EPI-Pakistan schedule (age)	Timeliness (days)	Coverage (n)	Drop-out	Lost to follow-up
**BCG**	0-365 days	0-28 days	Validity(n)/Children > 28days	No drop out as there is no second dose	Children who have not received BCG till 28 days after the scheduled date has passed and not returned for any vaccine past the scheduled date
**OPV -0**	0-28 days	0-28 days	Validity(n)/Children > 28days	N/A	Children who have not received OPV-0 till 28 days after the scheduled date has passed and not returned for any vaccine past the scheduled date
**OPV-1**	>=42 days	39-70 days	Validity(n)/Children >42days	OPV-1(n%) -OPV-0(n%) for children over 70 days	Children who have not received OPV-1 till 28 days after the scheduled date has passed and not returned for any vaccine past the scheduled date
**OPV-2**	>=4 weeks after OPV-1 & age >= 67 days	67-98 days	Validity(n)/Children>67 days and those having received OPV-1	OPV-2(n%)-OPV-1(n%) for children over 98 days	Children who have not received OPV-2 till 28 days after the scheduled date has passed and not returned for any vaccine past the scheduled date
**OPV-3**	>= 4 weeks after OPV-2 & age>= 95 days	95-126 days	Validity(n)/ Children>95days and those having received OPV-2	OPV-2(n%)-OPV-3(n%) for children over 126 days	Children who have not received OPV-3 till 28 days after the scheduled date has passed and not returned for any vaccine past the scheduled date
**Penta-1**	>=42 days	39-70 days	Validity(n)/Children >42days	Penta-1(n%) -Penta-0(n%) for children over 70 days	Children who have not received Penta-1 till 28 days after the scheduled date has passed and not returned for any vaccine past the scheduled date
**Penta-2**	>= 4 weeks after Penta-1 & >= 67 days	67-98 days	Validity(n)/Children>67 days and those having received Penta-1	Penta -2(n%)-Penta -1(n%) for children over 98 days	Children who have not received Penta-2 till 28 days after the scheduled date has passed and not returned for any vaccine past the scheduled date
**Penta-3**	>= 4 weeks after Penta-2 & >= 95 days	95-126 days	Validity(n)/ Children>95days and those having received Penta-2	Penta -2(n%)-Penta -3(n%) for children over 126 days	Children who have not received Penta-3 till 28 days after the scheduled date has passed and not returned for any vaccine past the scheduled date
**PCV-1**	>=42 days	39-70 days	Validity(n)/Children >42days	PCV-1(n%) -PCV-0(n%) for children over 70 days	Children who have not received PCV-1 till 28 days after the scheduled date has passed and not returned for any vaccine past the scheduled date
**PCV-2**	>=4 weeks after PCV-1& >= 67 days	67-98 days	Validity(n)/Children>67 days and those having received PCV-1	PCV -2(n%)-PCV -1(n%) for children over 98 days	Children who have not received PCV-2 till 28 days after the scheduled date has passed and not returned for any vaccine past the scheduled date
**PCV-3**	>=4 weeks after PCV-2& >= 95 days	95-126 days	Validity(n)/ Children>95days and those having received PCV-2	PCV -2(n%)-PCV -3(n%) for children over 126 days	Children who have not received PCV-3 till 28 days after the scheduled date has passed and not returned for any vaccine past the scheduled date
**ROTA-1**	>=42 days =<6months	39-70 days	Validity(n)/Children >42days	ROTA-1(n%) -ROTA-0(n%) for children over 70 days	Children who have not received ROTA-1 till 28 days after the scheduled date has passed and not returned for any vaccine past the scheduled date

Continues on next page

Continued from previous page

Vaccine	Validity as per EPI-Pakistan schedule (age)	Timeliness (days)	Coverage (n)	Drop-out	Lost to follow-up
**ROTA-2**	>4 weeks after ROTA-1 and age =<6months	67-98 days	Validity(n)/Children>67 days and those having received Rota-1	ROTA -2(n%)- ROTA -1(n%) for children over 98 days	Children who have not received ROTA-2 till 28 days after the scheduled date has passed and not returned for any vaccine past the scheduled date
**PCV**	>95days & age	95-126 days	Validity(n)/ Children>95days	N/A	Children who have not received PCV till 28 days after the scheduled date has passed and not returned for any vaccine past the scheduled date
**Measles1**	>39weeks	270-301days	Validity(n)/ Children>279days	N/A	Children who have not received Measles1 till 28 days after the scheduled date has passed and not returned for any vaccine past the scheduled date
**Measles2**	>52weeks &>4 weeks from Measles 1	453-484 days	Validity(n)/ Children453>days and those having received Measles-1	Measles -2(n%)- Measles -1(n%) for children over 453days	Children who have not received Measles2 till 28 days after the scheduled date has passed and not returned for any vaccine past the scheduled date
**Typhoid (TCV)**	>39weeks	270-301 days	Validity(n)/ Children>279days	N/A	Children who have not received TCV till 28 days after the scheduled date has passed and not returned for any vaccine past the scheduled date

11.4 ANNEX D. DISTRICT SELECTION FOR QUALITATIVE STUDY

TIMELINESS MEASLES			RANKING COVERAGE MEASLES 1			RANKING COMPLIANCE			
**Ranking**	**HH7**	**Measles**	**Ranking**	**Overall Coverage**	**Measles1**	**ZM Compliance Ranking (2018)**			
1	Karachi Central	24.4%	1	Karachi Central	77%	**Ranking**	**District**	**Overall Compliance Ranking**	
2	Karachi South	20.9%	2	Tharparkar	73%	1	Shaheed Benazirabad	67.07	Top 1/3rd segment
3	Karachi East	18.0%	3	Thatta	70%	2	Naushero Feroz	66.11	
4	Hyderabad	15.7%	4	District 29	70%	3	Hyderabad	59.93	Mid 1/3rd segment
5	Karachi West	15.4%	5	Sanghar	68%	4	Sukkur	54.65	
6	Karachi Malir	13.5%	6	Karachi East	67%	5	Karachi Central	51.44	Lower 1/3rd segment
7	District 29	10.4%	7	Hyderabad	66%	6	Thatta	48.19	
8	Thatta	9.5%	8	Naushahro Feroze	65%	7	Karachi West	46.93	
9	Naushahro Feroze	8.6%	9	Karachi South	65%	8	Karachi East	44.17	
10	Dadu	8.1%	10	Tando Allahyar	64%	9	Sanghar	42.88	
11	Khairpur	8.0%	11	Matiari	64%	10	Tando Allahyar	41.65	
12	Shaheed Benazirabad	7.8%	12	Jamshoro	63%	11	Ghotki	41.41	
13	Tharparkar	7.0%	13	Umer Kot	63%	12	Mirpurkhas	41.19	
14	Larkana	6.7%	14	Mirpur Khas	61%	13	Larkana	40.64	
15	Mirpur Khas	6.7%	15	Karachi Malir	59%	14	Kambar	40.61	
16	Tando Allahyar	6.1%	16	Tando Muhammad Khan	58%	15	Tando Muhammad Khan	40.42	
17	Sukkur	6.1%	17	Badin	58%	16	Umerkot	40.29	
18	Jamshoro	5.5%	18	Shikarpur	58%	17	Kashmore	39.99	
19	Shikarpur	5.4%	19	Shaheed Benazirabad	55%	18	Karachi South	38.46	
20	Matiari	5.4%	20	Dadu	54%	19	Kemari	38.23	
21	Ghotki	4.8%	21	Karachi West	54%	20	Sujawal	36.54	
22	Sujawal	4.8%	22	Ghotki	54%	21	Korangi	35.68	
23	Sanghar	4.2%	23	Khairpur	53%	22	Malir	34.87	
24	Tando Muhammad Khan	4.2%	24	Larkana	50%	23	Jamshoro	32.66	
25	Umer Kot	3.1%	25	Sujawal	49%	24	Badin	28.08	
26	Shahdad Kot	2.8%	26	Sukkur	47%	25	Jacobabad	27.73	
27	Badin	1.5%	27	Jacobabad	47%	26	Shikarpur	24.20	
28	Jacobabad	1.5%	28	Shahdad Kot	42%	27	Matiari	13.24	
29	Kashmore	1.0%	29	Kashmore	27%	28	Tharparkar	7.23	
						29	Khairpur	41.2%	

11.5 ANNEX E. TRAINING OF VACCINATION STAFF ON ZM-EIR APPLICATION – SEPT-DEC 2017

S. No	Dates	Division	District(s)	Town(s)	No. of EPI Staff
**1**	September 6-8, 2017	Karachi	KHI Korangi	Shah Faisal & Malir (Model)	28
**2**	September 7-9, 2017	Karachi	Central	Liaqatabad	40
**3**	September 7-9, 2017	Karachi	Central	North Karachi	40
**4**	September 7-9, 2017	Karachi	East	Gulshan-e-Iqbal	25
**5**	September 7-9, 2017	Karachi	Malir	Malir	24
**6**	September 7-9, 2017	Karachi	West	Baldia	13
**7**	September 7-9, 2017	Karachi	West	Kemari	28
**8**	September 11-13, 2017	Karachi	Central	Gulberg	22
**9**	September 11-13, 2017	Karachi	East	Jamshed	35
**10**	September 11-13, 2017	Karachi	Korangi	Landhi	18
**11**	September 11-13, 2017	Karachi	Malir	Bin Qasim	29
**12**	September 11-13, 2017	Karachi	West	Orangi	37
**13**	September 14-16, 2017	Karachi	Korangi	Korangi	26
**14**	October 2-4, 2017	Karachi	Malir	Gadap	12
**15**	October 2-4, 2017	Karachi	East and West	Gadap	17
**16**	October 3-5, 2017	Karachi	South	Saddar	38
**17**	October 3-5, 2017	Karachi	South	Lyari	36
**18**	October 5-7, 2017	Karachi	West	SITE	31
**19**	October 5-7, 2017	Karachi	Central	North Nazimbad	17
**20**	October 2-4, 2017	Hyderabad	Hyderabad	Hyderabad (Rural)	23
**21**	October 5-7, 2017	Hyderabad	Hyderabad	Qasimabad	23
**22**	October 5-7, 2017	Hyderabad	Jamshoro	Jamshoro and Kotri	21
**23**	October 5-7, 2017	Hyderabad	Matiari	Matiari	21
**24**	October 5-7, 2017	Hyderabad	Tando Allah Yar	Jhando Murree & Chamber	18
**25**	October 9-11, 2017	Hyderabad	Hyderabad	Hyderabad City	22
**26**	October 9-11, 2017	Hyderabad	Jamshoro	Sehwan	27
**27**	October 9-11, 2017	Hyderabad	Matiari	Hala & Saeedabad	23
**28**	October 9-11, 2017	Hyderabad	Tando Allah Yar	Tando Allah Yar	23
**29**	October 9-11, 2017	Hyderabad	Tando M Khan	Tando M Khan & Tando Ghulam Hyder	23
**30**	October 9-11, 2017	Hyderabad	Tando M Khan	Bulri Shah Karim	17
**31**	October 12-15, 2017	Hyderabad	Jamshoro	Manhjhand	10
**32**	October 16-18, 2017	Hyderabad	Hyderabad	Latiafabad	32
**33**	October 12-14, 2017	Hyderabad	Hyderabad	Hyderabad City	29
**34**	October 16-18, 2017	Hyderabad	Jamshoro	Thano Bola Khan	8
**35**	October 19-21, 2017	Hyderabad	Hyderabad	Latiafabad	30
**36**	November 9-11, 2017	Sukkur	Ghotki	Ghotki/khanghar	33
**37**	November 13-15, 2017	Sukkur	Ghotki	Mirpur / Ghotki	21

Continues on next page

Continued from previous page

S. No	Dates	Division	District(s)	Town(s)	No. of EPI Staff
**38**	November 16-18, 2017	Sukkur	Ghotki	Daharki/ubaro	29
**39**	November 9-11, 2017	Sukkur	Sukkur	November 20-22, 2017	30
**40**	November 13-15, 2017	Sukkur	Sukkur	New Sukkur	23
**41**	November 16-18, 2017	Sukkur	Sukkur	Rohri & Salehpat	33
**42**	November 23-25, 2017	Sukkur	Sukkur	Pano Aqil	28
**43**	November 9-11, 2017	Bhambhore	Badin	Badin	23
**44**	November 13-15, 2017	Bhambhore	Badin	Badin & Talhar	23
**45**	November 16-18, 2017	Bhambhore	Badin	Matli	26
**46**	November 16-18, 2017	Bhambhore	Badin	Tando Bago	21
**47**	November 23-25, 2017	Bhambhore	Badin	Golarchi	13
**48**	November 9-11, 2017	Bhambhore	Sajawal	Mirpur Bathoro	27
**49**	November 13-15, 2017	Bhambhore	Sajawal	Sujawal , Jati & Shah Bander	36
**50**	November 9-11, 2017	Bhambhore	Thatta	Thatta	39
**51**	November 13-15, 2017	Bhambhore	Thatta	Mirpur Sakro	23
**52**	November 16-18, 2017	Bhambhore	Thatta	Ghora Bari, Keti Bunder & Kharo Chaan	13

11.6 ANNEX E: DISTRICT LEVEL ANALYSES

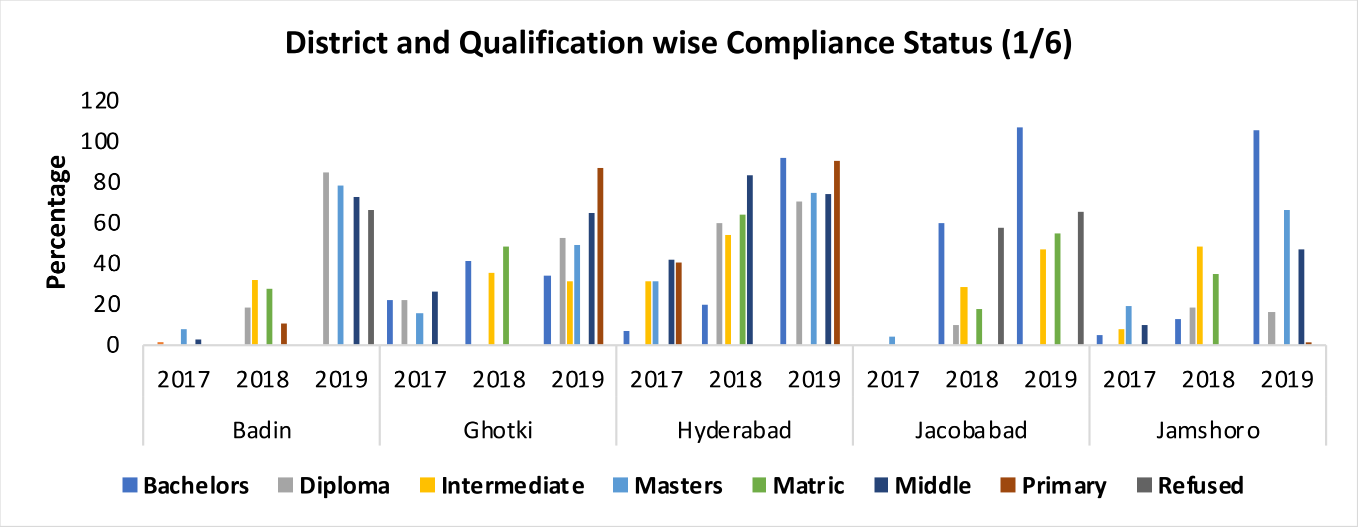

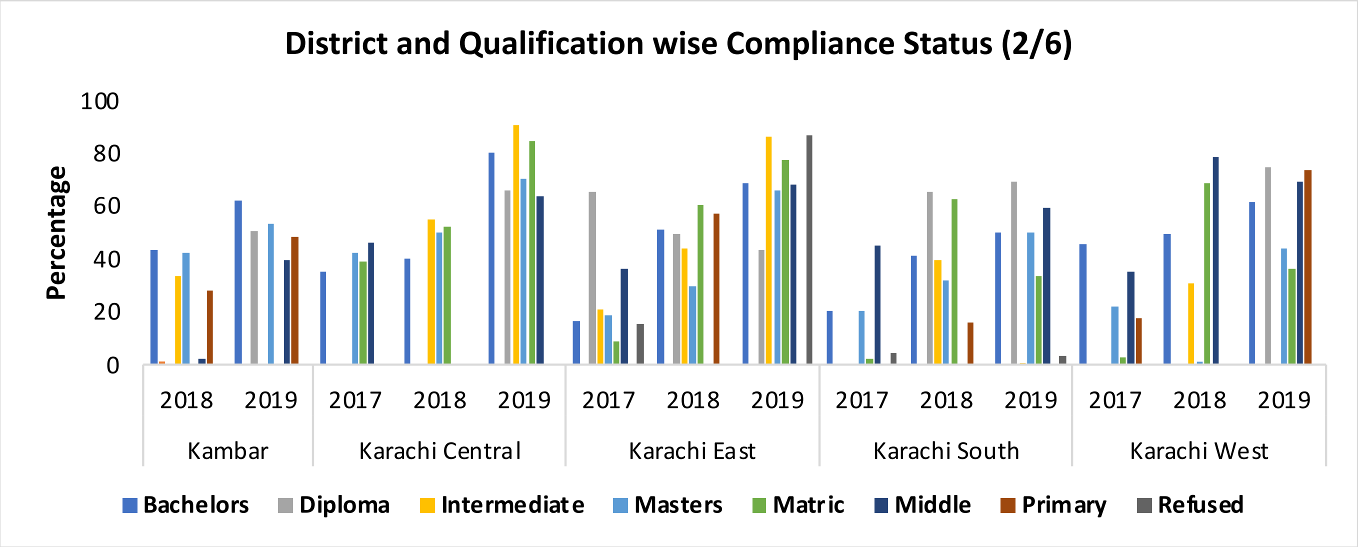

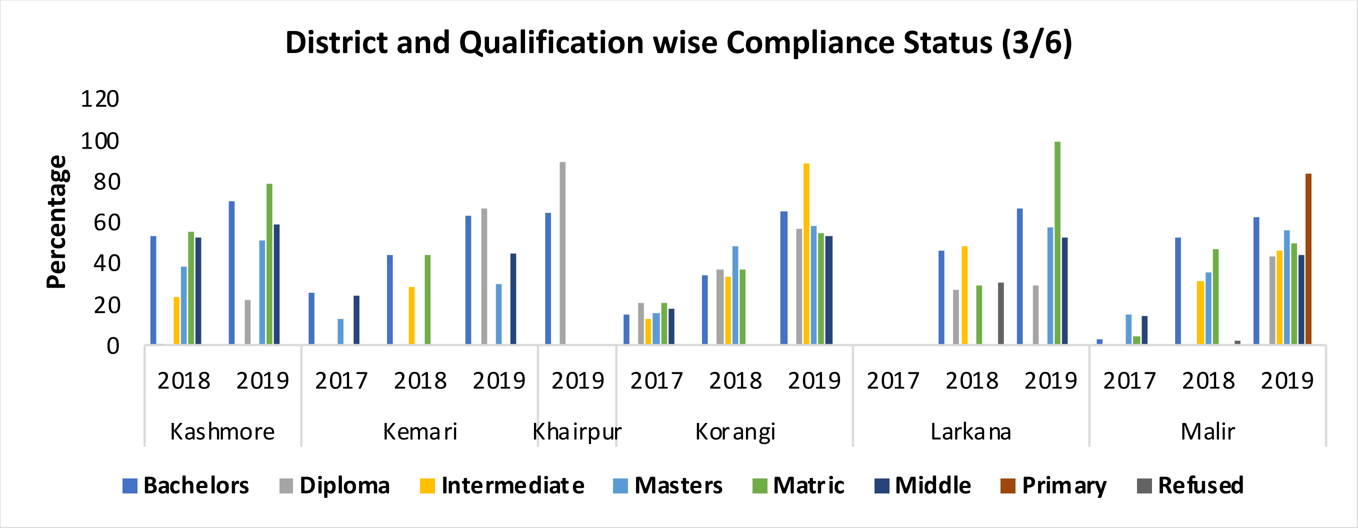

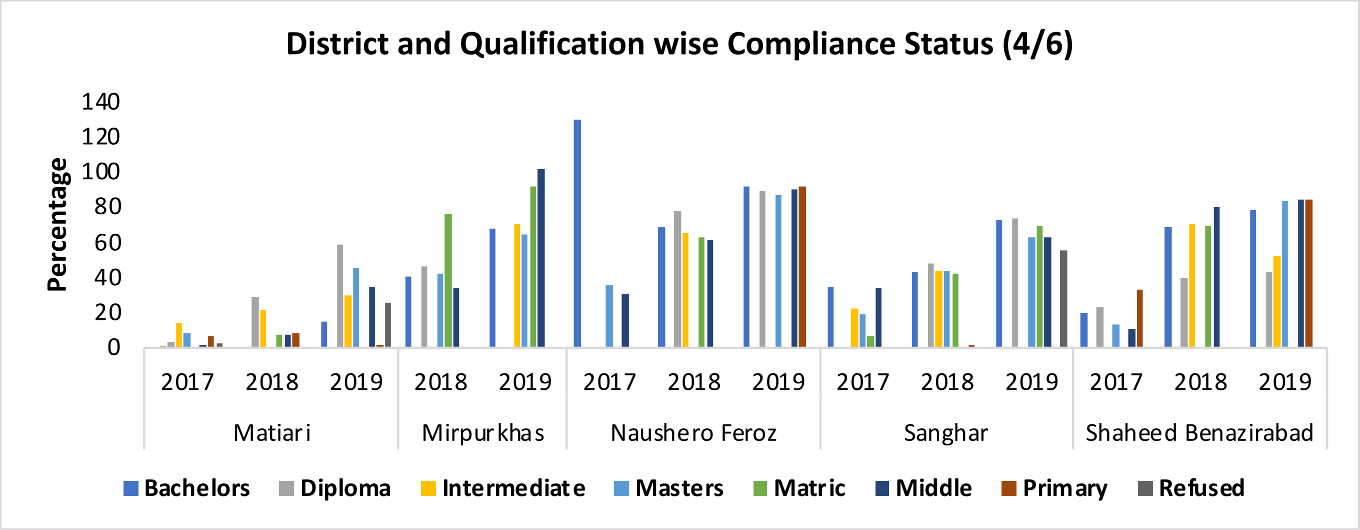

District and Qualification wise Compliance Status (4/6)

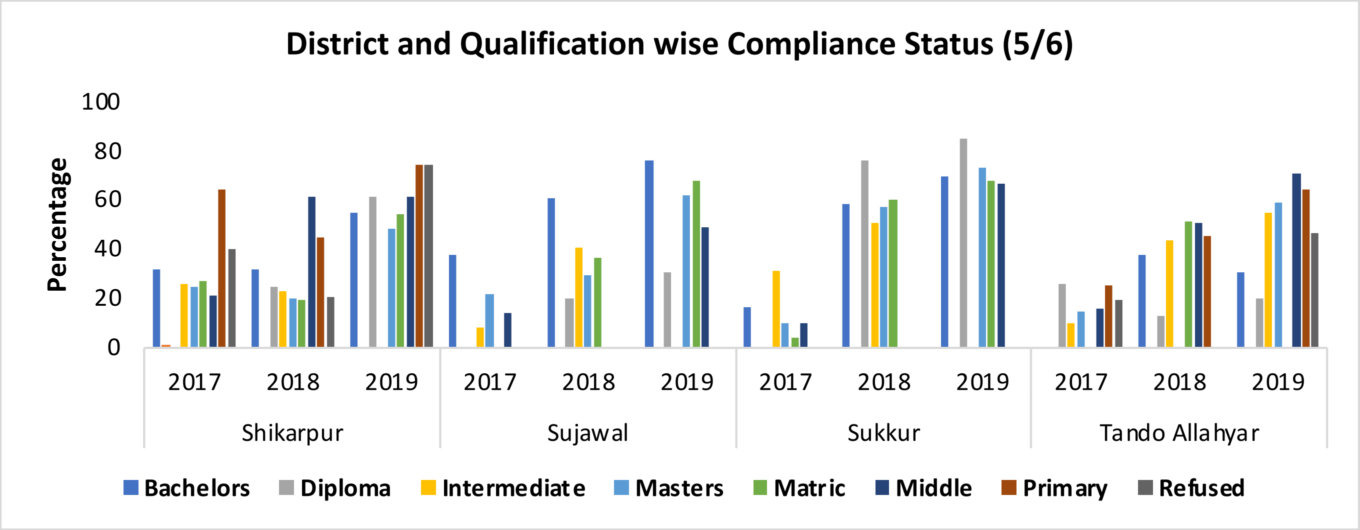

District and Qualification wise Compliance Status (5/6)

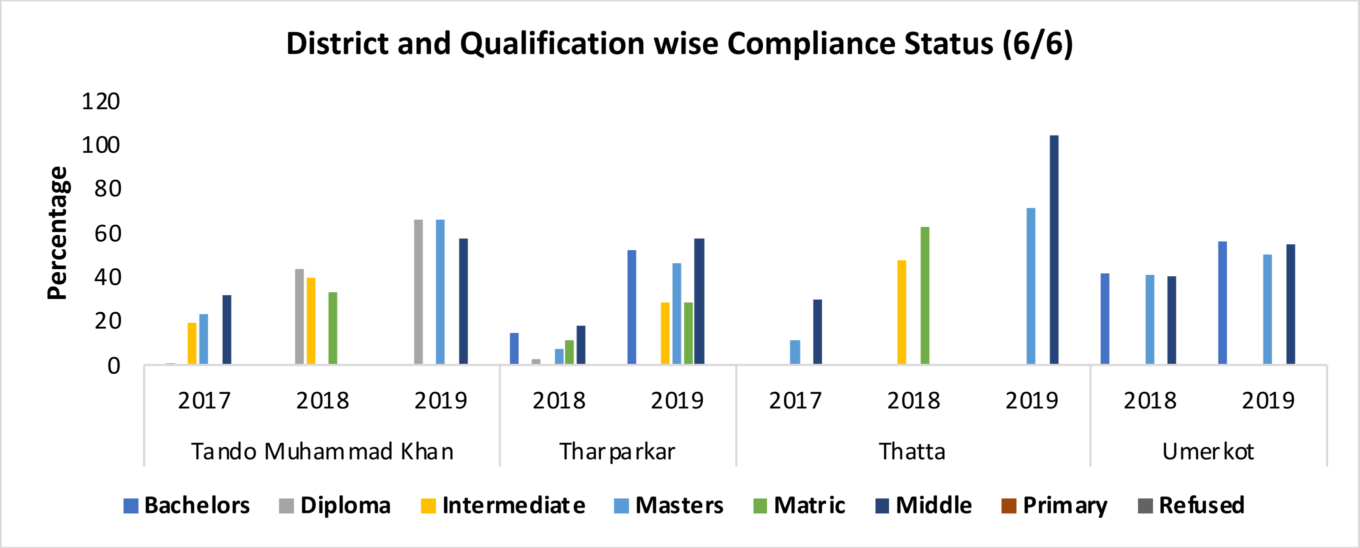

District and Qualification wise Compliance Status (6/6)

B: DISTRICT LEVEL COMPLIANCE OF USAGE BY VACCINATION STAFF ACCORDING TO URBAN VS RURAL STATUS

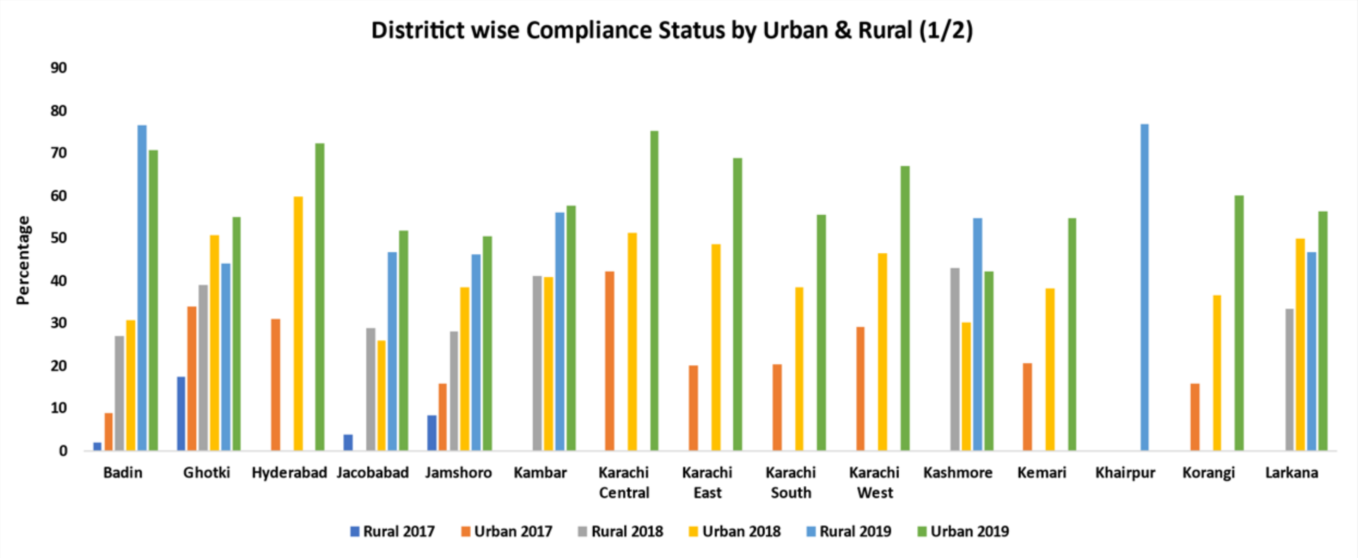

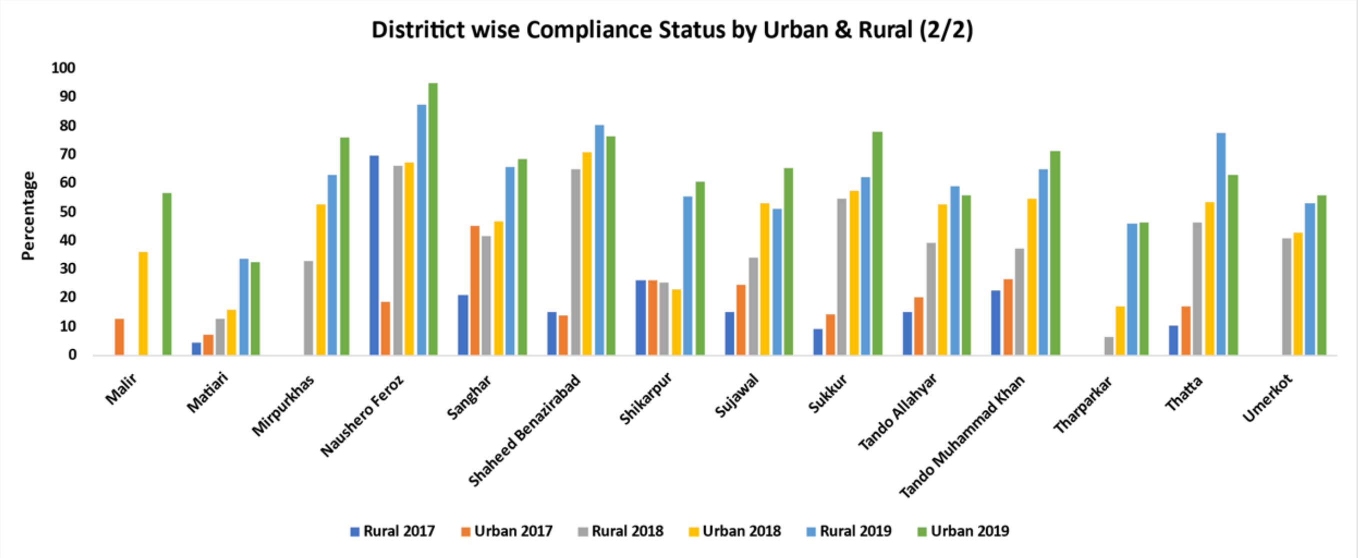

C: DISTRICT LEVEL COMPLIANCE OF USAGE BY VACCINATION STAFF ACCORDING TO AGE GROUP OF VACCINATION STAFF

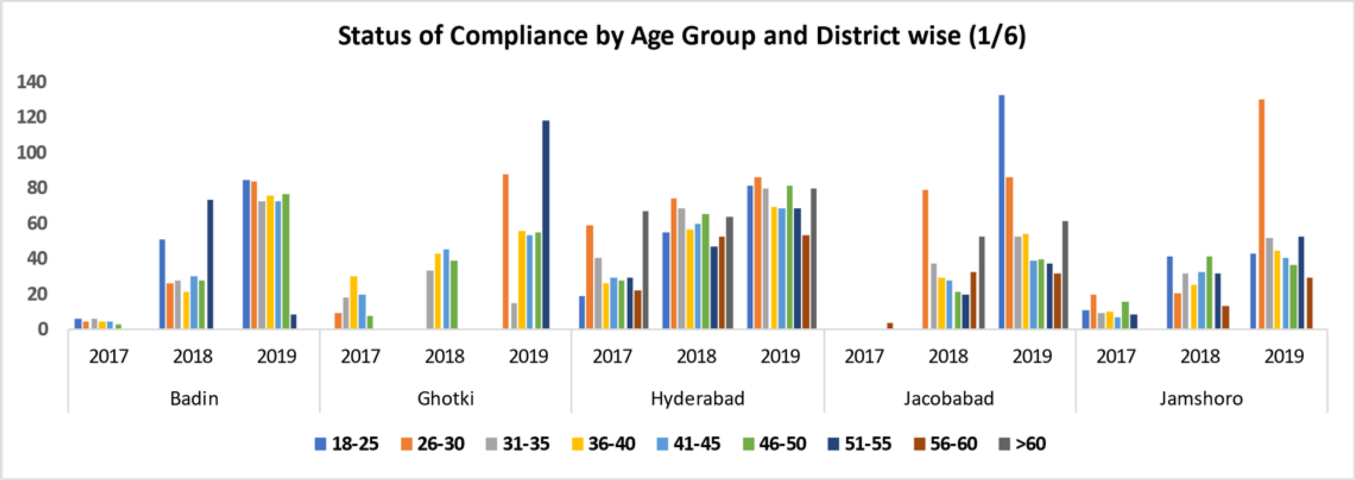

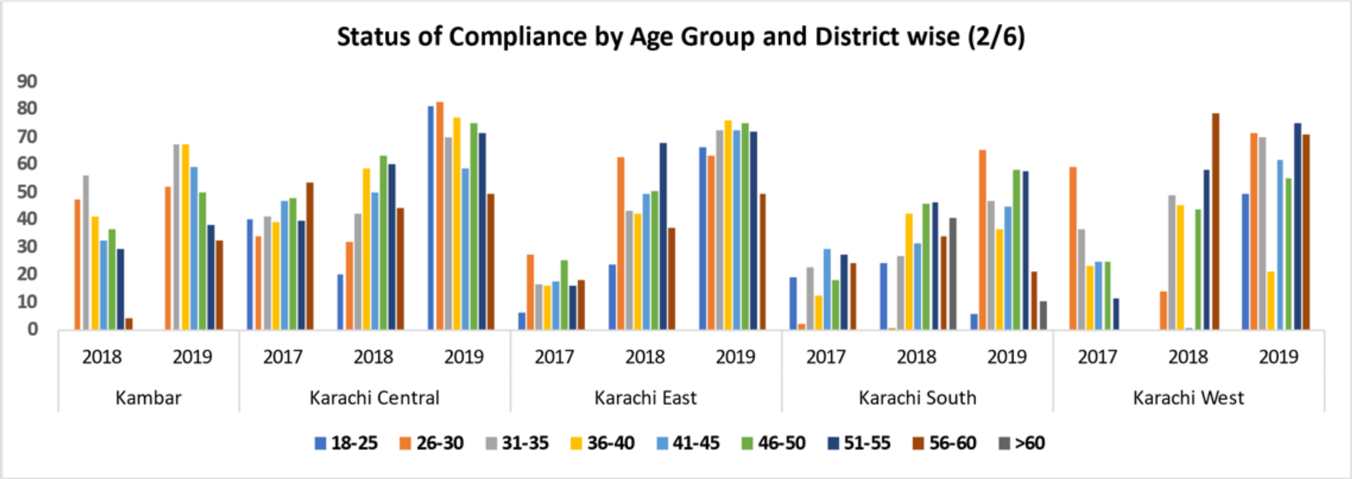

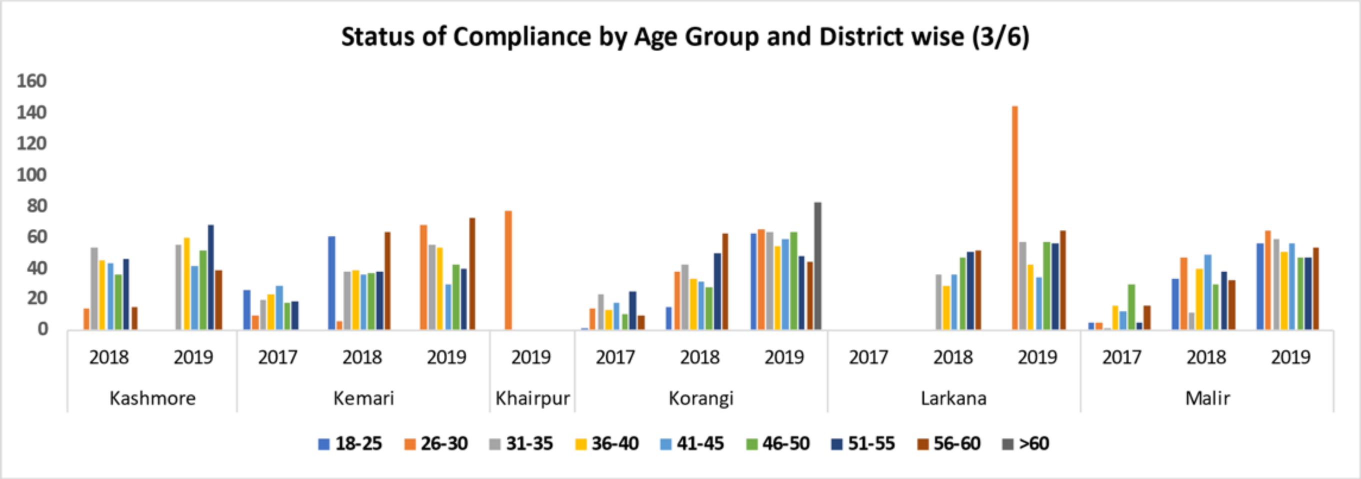

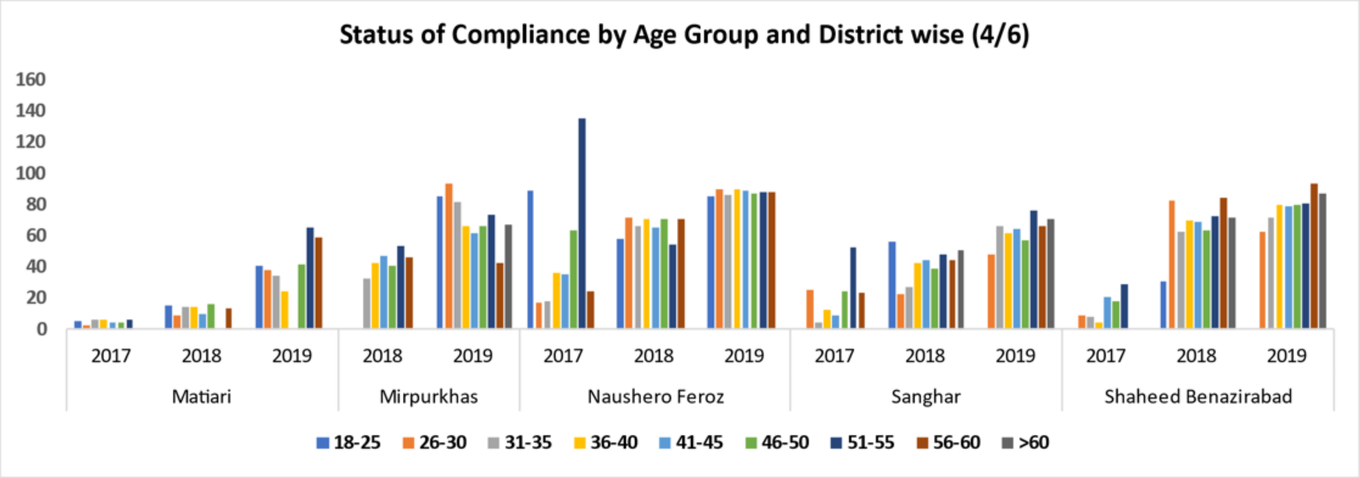
Status of Compliance by Age Group and District wise (4/6)
160
140
120
100
80
60
40
20
0
2017
2018
2019
2018
2019
2017
2018
2019
2017
2018
2019
2017
2018
2019
Matiari
Mirpurkhas
Naushero Feroz
Sanghar
Shaheed Benazirabad
18-25
26-30
31-35
36-40
41-45
46-50
51-55
56-60
>60

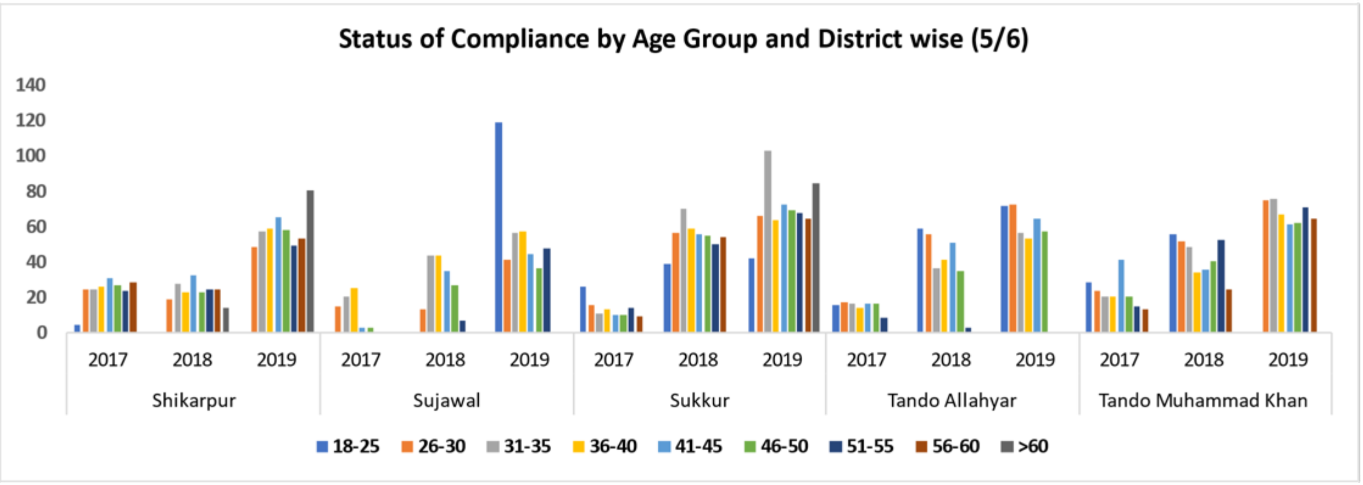
Status of Compliance by Age Group and District wise (5/6)
140
120
100
80
60
40
20
0
2017
2018
2019
2017
2018
2019
2017
2018
2019
2017
2018
2019
2017
2018
2019
Shikarpur
Sujawal
Sukkur
Tando Allahyar
Tando Muhammad Khan
18-25
26-30
31-35
36-40
41-45
46-50
51-55
56-60
>60

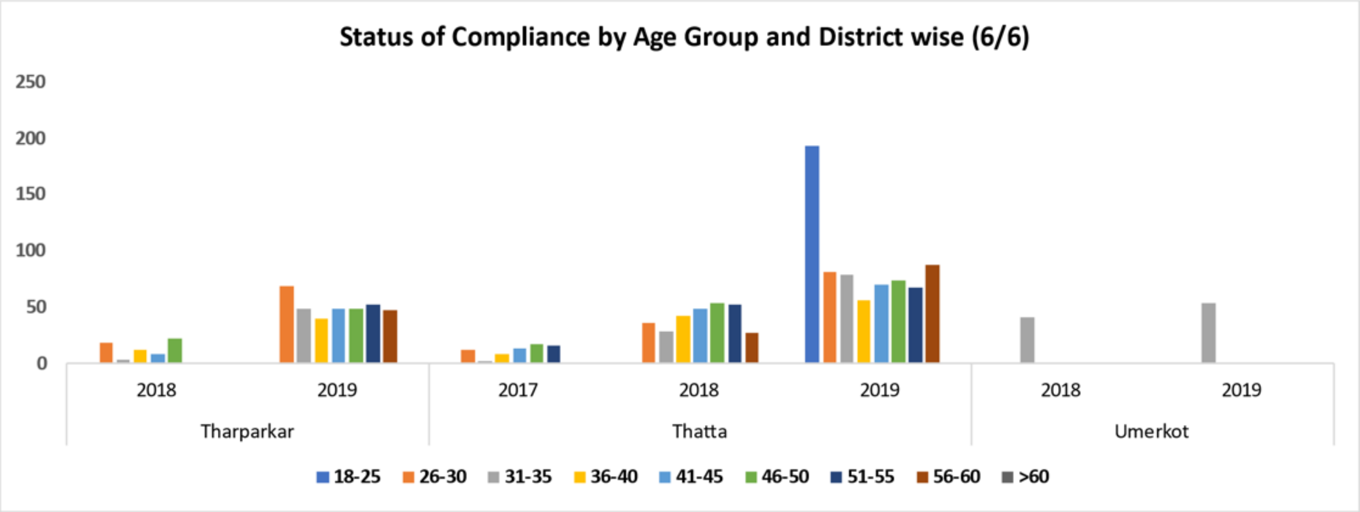
Status of Compliance by Age Group and District wise (6/6)
250
200
150
100
50
0
2018
2019
2017
2018
2019
2018
2019
Tharparkar
Thatta
Umerkot
18-25
26-30
31-35
36-40
41-45
46-50
51-55
56-60
>60

D: DISTRICT LEVEL LOG-INS BASED USAGE BY SUPERVISORY AND MANAGERIAL STAFF CADRES

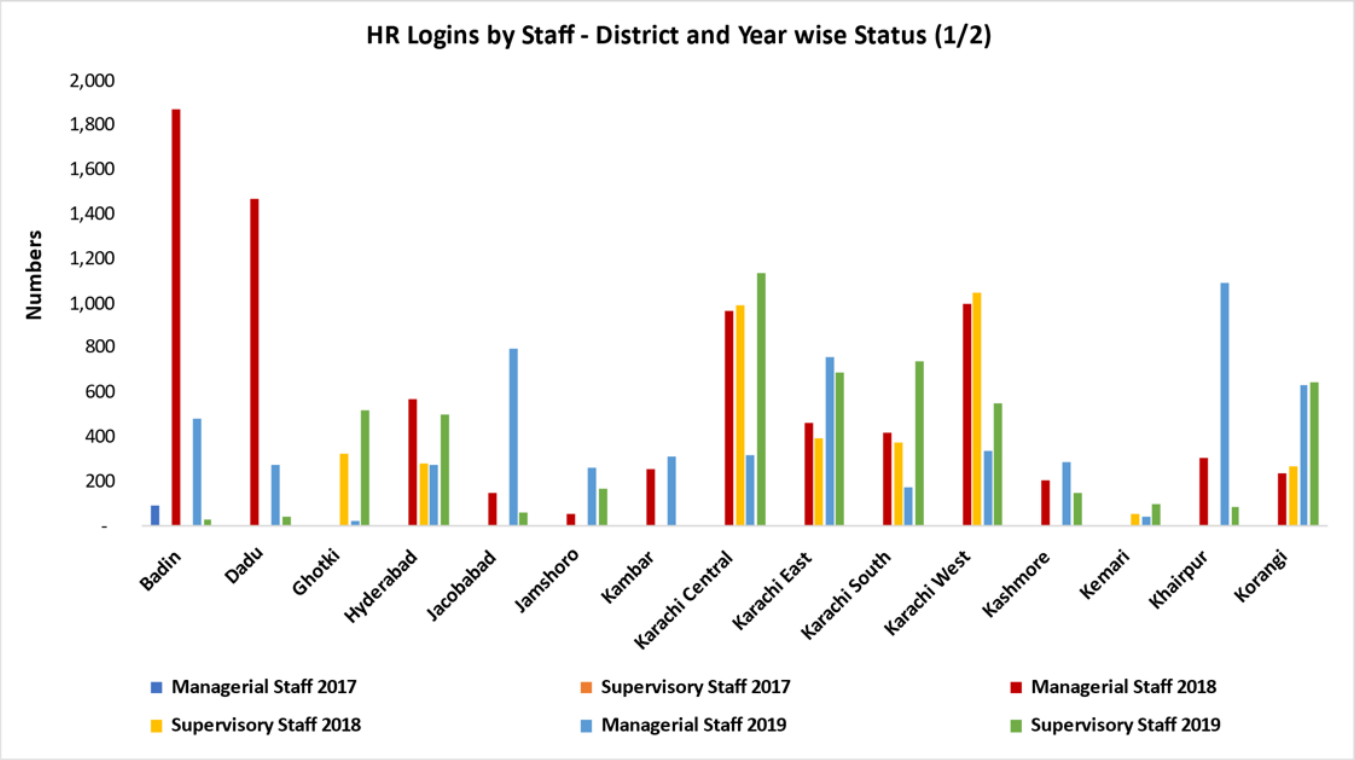

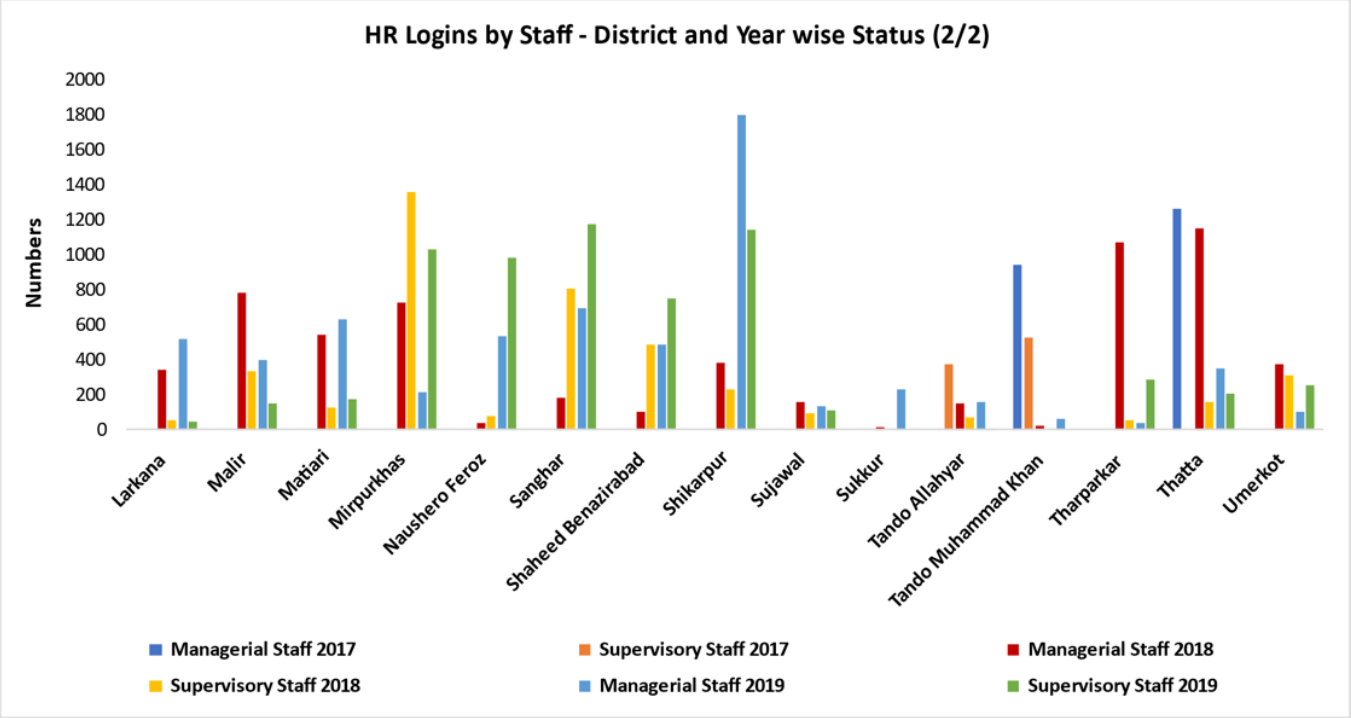

Supplement: Multimedia Appendix 1 [file jmir_v26i1e52792_app1.pdf]
